# Angiogenesis-Related Genes Predict Outcomes and Immune Traits in Skin Melanoma

**DOI:** 10.3390/ijms26178254

**Published:** 2025-08-26

**Authors:** Latchezara Vladova, Ilias Georgakopoulos-Soares, Apostolos Zaravinos

**Affiliations:** 1 Department of Life Sciences, School of Sciences, European University Cyprus, Nicosia 1516, Cyprus; latchivladova@gmail.com (L.V.); izg5139@psu.edu (I.G.-S.); 2 Cancer Genetics, Genomics and Systems Biology Laboratory, Basic and Translational Cancer Research Center (BTCRC), Nicosia 1516, Cyprus; 3 Department of Biochemistry and Molecular Biology, Institute for Personalized Medicine, Penn State University College of Medicine, Hershey, PA 17033, USA

**Keywords:** angiogenesis-related genes, skin melanoma, tumor microenvironment, angiogenesis, immunotherapy, biomarkers

## Abstract

The interplay between angiogenesis and the immune system is intricate, with the potential to either enhance or repress the immune response. Angiogenesis-related genes (ARGs) are significant for the development, growth, and immune response of tumors. Understanding their prognostic significance and molecular characteristics in skin melanoma can guide and refine therapeutic strategies. Here, we analyzed the TCGA-SKCM dataset and explored the ARG expression between skin melanoma and normal skin, as well as between primary and metastatic tumors. Kaplan–Meier analyses were conducted to assess the overall, disease-specific, and progression-free survival. Additionally, comprehensive immune profiling was carried out utilizing advanced bioinformatics tools to evaluate immune checkpoint gene expression and immune cell infiltration. Our findings highlighted strong prognostic associations for *S100A4*, *ITGAV*, and *COL3A1*. Molecular characterization showed a significant upregulation of *PTK2*, *CXCL6*, *COL3A1*, *COL5A2*, *PF4*, *TNFRSF21*, *LRPAP1*, *VTN*, *TIMP1*, *SPP1*, and *OLR1* in SKCM compared to that in normal skin. Immune analyses, including Immune Checkpoint Gene Analysis, Immune Infiltration Analysis, Immune Cell Analysis, and Immune Cell Profiling, demonstrated both positive and negative correlations between ARGs expression and immune cell infiltration, emphasizing the multifaceted role of these genes in immune modulation. The study underscores the prognostic relevance of ARGs in skin melanoma and their contribution to tumor immunity. Overall, our findings expand our understanding of melanoma immunogenetics, suggesting the use of angiogenesis-related genes not merely as vascular regulators, but also as immune modulators.

## 1. Introduction

Malignant melanomas develop from the malignant transformation of melanocytes [1,2,3]. Skin melanoma is characterized by high metastatic potential and significant morbidity and mortality [2,4]. The melanogenic activity and behavior of melanocytes are regulated by various factors, such as ultraviolet radiation (UVR) and a range of chemical and biological mediators, which consist of both hormonal and non-hormonal regulators, in addition to genetic and molecular ones [5,6,7]. The most prevalent types of melanomas include cutaneous malignant melanomas which significantly impact large segments of the population with high incidence and mortality rates relative to those of other cancers [8].

Skin melanoma is the 17th most prevalent cancer globally. Although Europe accounts for the highest absolute number of cases and death, the highest incidence and mortality rates per capita are reported in Australia and New Zealand [8,9]. Four major types of melanoma skin cancer exist. Superficial spreading melanoma, accounting for 60–70% of cases, typically grows outward on the skin and can occur anywhere on the body, while nodular melanoma (15–30%) tends to grow vertically into the skin; lentigo maligna melanoma, representing about 8% of cases, is often amelanotic and appears red or skin-colored, whereas acral lentiginous melanoma, the rarest subtype, presents in advanced stages as a palpable pigmented macule with variable coloration [4,8,10,11,12].

Angiogenesis—the formation of new blood vessels from pre-existing vasculature—is a key process in tumor development and progression, supplying oxygen and other nutrients vital for tumor growth and survival [13,14,15]. The prognostic significance of angiogenesis in melanoma has garnered increasing interest, with several studies linking enhanced angiogenic activity to tumor aggressiveness and poor clinical outcomes [16,17]. Recent advances in genomic and molecular biology have brought into the light the importance of angiogenesis-related genes (ARGs) in the growth and development of tumors, as well as potential therapeutic targeting [15,18]. Melanoma, recognized for its aggressive nature and rapid metastatic potential, remains a significant clinical challenge in terms of management and treatment [4,5]. Furthermore, ARGs play a role in regulating the tumor microenvironment (TME), modulating immune responses, and contributing to immune evasion mechanisms—factors that can profoundly influence the efficacy of immunotherapeutic approaches [19,20,21,22,23]. Therefore, a comprehensive understanding of the molecular and functional characteristics of ARGs could provide valuable insights for developing novel targeted therapies and improving clinical outcomes for melanoma patients [16,24,25,26,27,28].

This study aims to comprehensively analyze the role of ARGs in skin melanoma, focusing on their prognostic significance, molecular characteristics, and interactions with tumor immunity.

## 2. Results

### 2.1. Gene Expression Prognositc Analysis of the Angiogenesis-Related Genes

**Figure 1:**
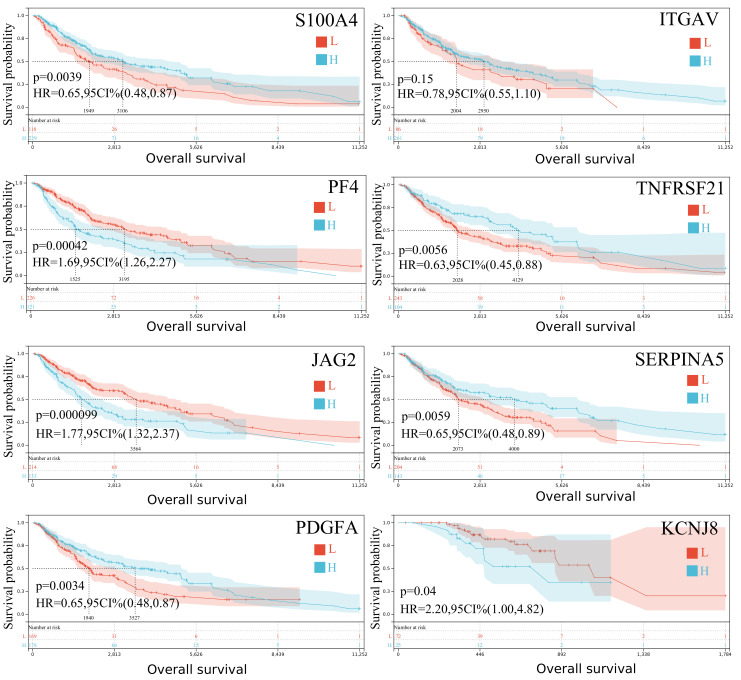
The Kaplan–Meier curves depict the overall survival probability for both primary and metastatic skin melanoma patients with high (H) and low (L) expression of *S100A4, ITGAV, PF4, TNFRSF21, JAG2, SERPINA5, PDGFA* and *KCNJ8*, respectively. HR, hazard ratio; CI, confidence interval.

We first examined the expression of ARGs in primary (SKCM-P) and metastatic (SKCM-M) skin melanoma samples, and found that several genes are strongly associated with survival outcomes. For instance, in SKCM-M, *S100A4*, *ITGAV*, *PF4*, *TNFRSF21*, *JAG2*, *SERPINA5*, *PDGFA* and *KCNJ8* emerged as significant prognostic markers (Figure 1). These genes—already linked to cell growth and angiogenesis—appear to influence disease progression and metastatic potential; therefore, their expression may help predict patient outcomes and guide personalized treatment strategies for advanced melanoma.

In contrast, *COL3A1* and *FSTL1* were significantly overexpressed in SKCM-P, pointing to a potential role in early-stage tumor development and offering targets for early detection and intervention.

**Figure 2:**
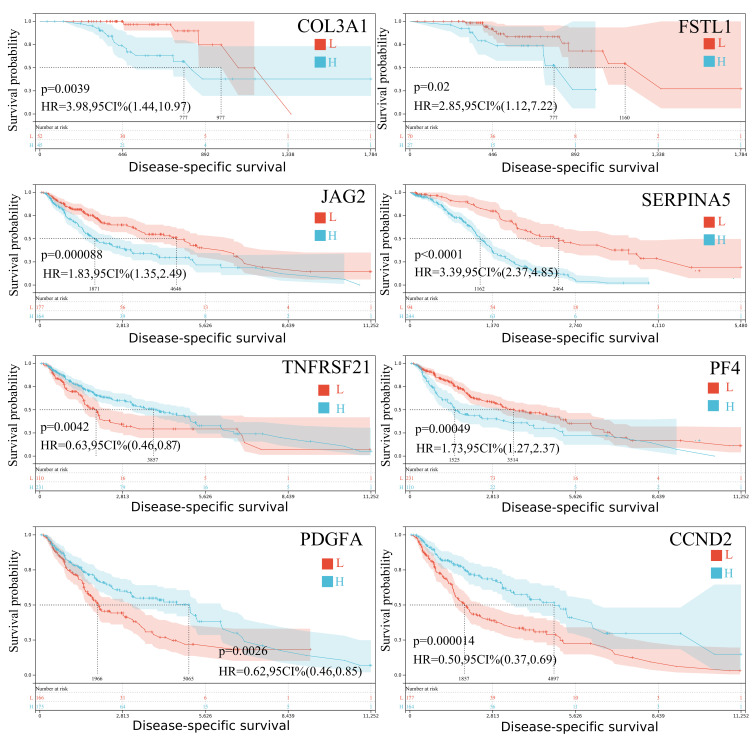
The Kaplan–Meier curves depict the disease-specific survival probability of primary and metastatic skin melanoma patients with high (H) or low (L) expression of *COL3A1*, *FSTL1*, *PF4*, *TNFRSF21*, *JAG2*, *SERPINA5*, *CCND2*, and *PDGFA*, respectively. HR, hazard ratio; CI, confidence interval.

*COL3A1* and *FSTL1* showed prognostic significance for disease-specific survival, in the SKCM-P dataset. Known for regulating extracellular matrix dynamics and tissue remodeling, these genes are closely tied to early tumor development and may serve as markers for early detection and intervention. In SKCM-M, *PF4*, *TNFRSF21*, *JAG2*, *SERPINA5*, *CCND2*, and *PDGFA* were associated with survival outcomes (Figure 2). These genes influence inflammation, cell proliferation, and angiogenesis—key processes in melanoma progression—and could inform targeted treatment strategies for metastatic disease. Notably, *TIMP1* emerged as a strong prognostic marker in both primary and metastatic melanoma. Its consistent expression across disease stages suggests it may be a valuable biomarker for tracking the tumor microenvironment (TME), monitoring disease progression, and evaluating treatment response throughout the course of the disease.

**Figure 3:**
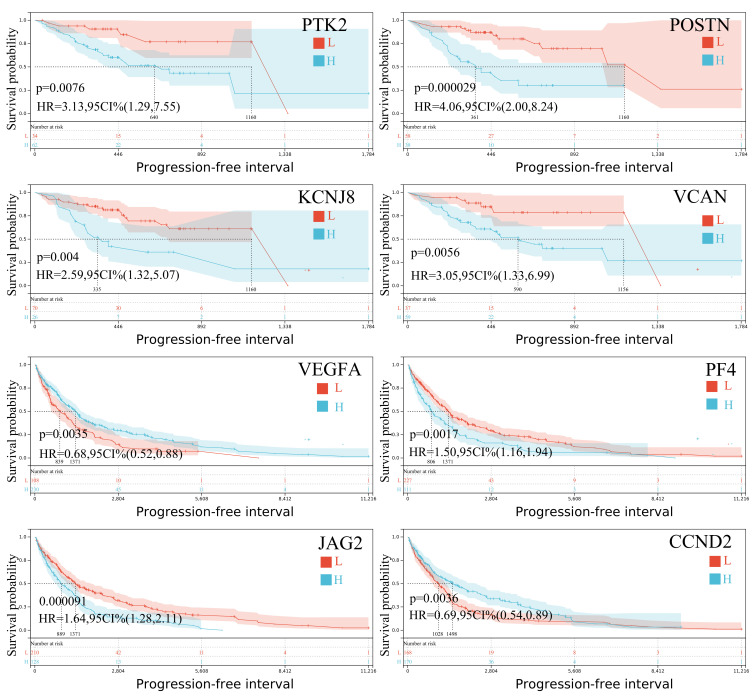
The Kaplan–Meier curves depict the progression-free survival probability of metastatic skin melanoma patients with high (H) or low (L) expression of *PTK2, KCNJ8, POSTN, VCAN, VEGFA, PF4, JAG2*, and *CCND2*, respectively. HR, hazard ratio; CI, confidence interval.

Progression-free interval analysis also revealed several prognostic genes. In SKCM-P, *PTK2*, *VEGFA*, *KCNJ8*, *POSTN*, and *VCAN* were significantly associated with patient outcomes, while in SKCM-M, *VEGFA*, *PF4*, *JAG2*, *CCND2*, and *TIMP1* showed strong prognostic value (Figure 3). Notably, *VEGFA* and *SPP1* were significant prognosticators both in primary and metastatic tumors, underscoring their broader role in melanoma progression. These distinct expression patterns between primary and metastatic skin melanomas highlight the molecular complexity and heterogeneity of the disease, offering important clues for developing more precise, stage-specific therapeutic strategies.

### 2.2. Differential Gene Expression Analysis of the Angiogenesis-Related Genes

**Figure 4:**
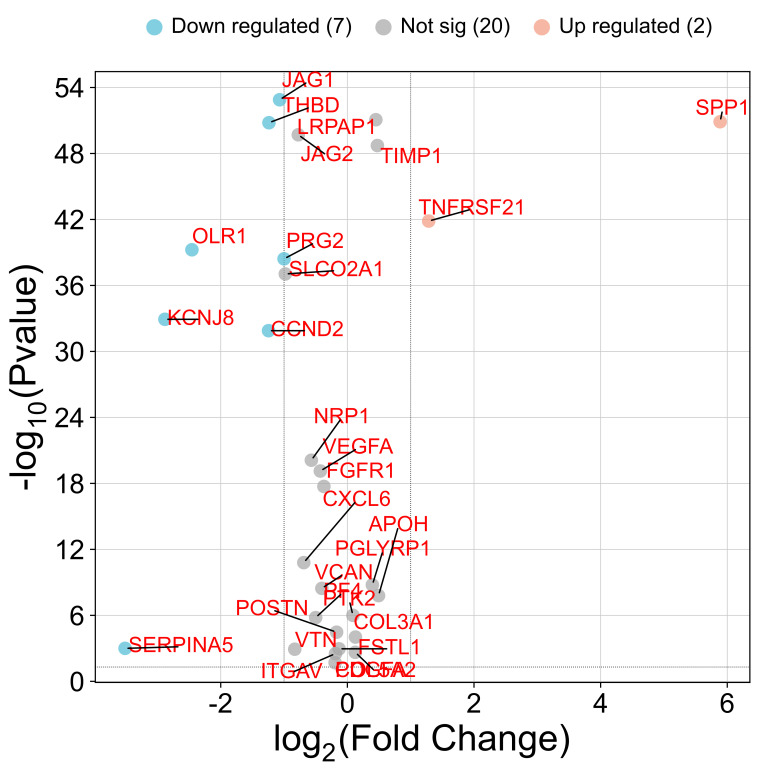
Volcano plot depicting the significantly upregulated and downregulated angiogenesis-related genes in skin melanoma compared to those in normal skin.

We observed significant differences in ARG expression between melanoma and normal tissue. Eleven genes—including *TNFRSF21* and *SPP1*—were markedly upregulated in tumors, suggesting active angiogenic signaling that may drive melanoma progression and metastasis (Figure 4). Conversely, eighteen genes, including *SERPINA5*, *CCND2*, *KCNJ8*, *PRG2*, *OLR1*, *THBD*, and *JAG1*, were downregulated (Figure 4), pointing to the involvement of alternative, non-VEGFA-driven angiogenic pathways. These contrasting patterns underscore the complexity of angiogenesis regulation in melanoma and reveal potential therapeutic targets within these disrupted networks.

### 2.3. Immune Checkpoint Gene Analysis

**Figure 5:**
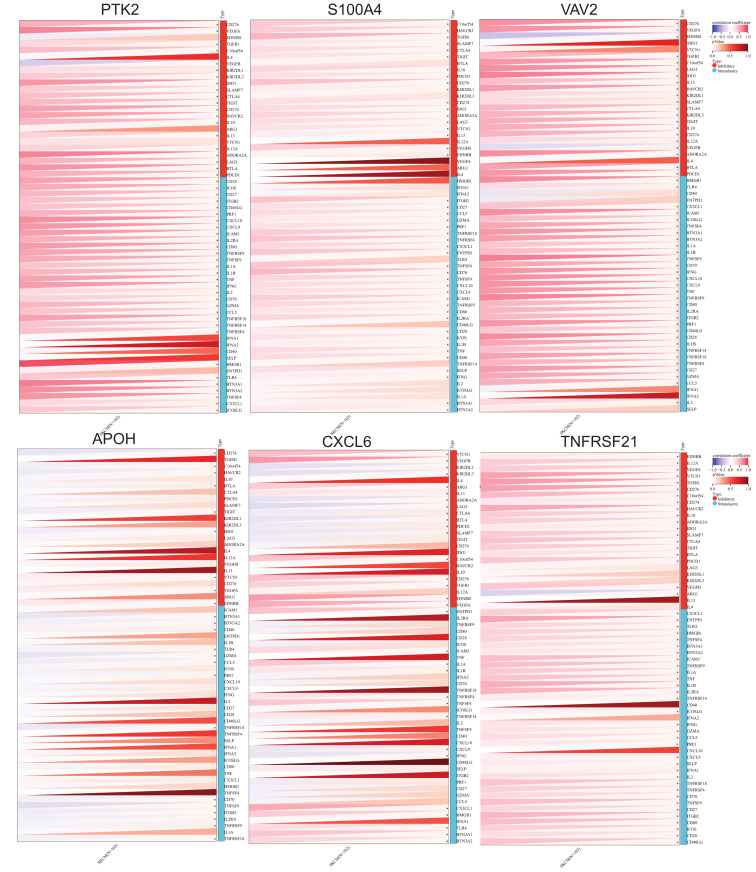
Pearson’s correlations between the expression of *PTK2*, *S100A4*, *VAV2*, *APOH*, *CXCL6*, and *TNFRSF21* and inhibitory (or stimulatory) immune checkpoints in skin melanoma. An asterisk (*) denotes *p* < 0.05.

Analysis of immune checkpoint gene expression revealed significant correlations between ARGs and immune regulation in SKCM. Several ARGs—including *PTK2*, *S100A4*, *CXCL6*, *VAV2, APOH*, and *TNFRSF21*—showed strong positive correlations with inhibitory immune checkpoints such as *CD276, VEGFA, VEGFB, TGFB1, IDO1, TIGIT, CTLA4, CD274, SLAMF7, HAVCR2, IL10, IL13, IL12A, ADORA2A, LAG3, BTLA*, and *PDCD1*. This suggests these ARGs may contribute to enhanced immune checkpoint activity, potentially aiding tumor immune evasion. Interestingly, *CXCL6* and other ARGs also showed negative correlations with checkpoints like *VEGFB*, *KIR2DL1*, *LAG3*, *CTLA4*, *PDCD1*, and *IFNG*, indicating they may promote immune surveillance and anti-tumor responses (Figure 5). These results highlight the intricate link between angiogenesis and immune modulation in melanoma and underscore the potential for ARGs to serve as dual biomarkers for angiogenic and immune-related therapeutic targeting.

### 2.4. Immune Infiltration Analysis (ESTIMATE)

**Figure 6:**
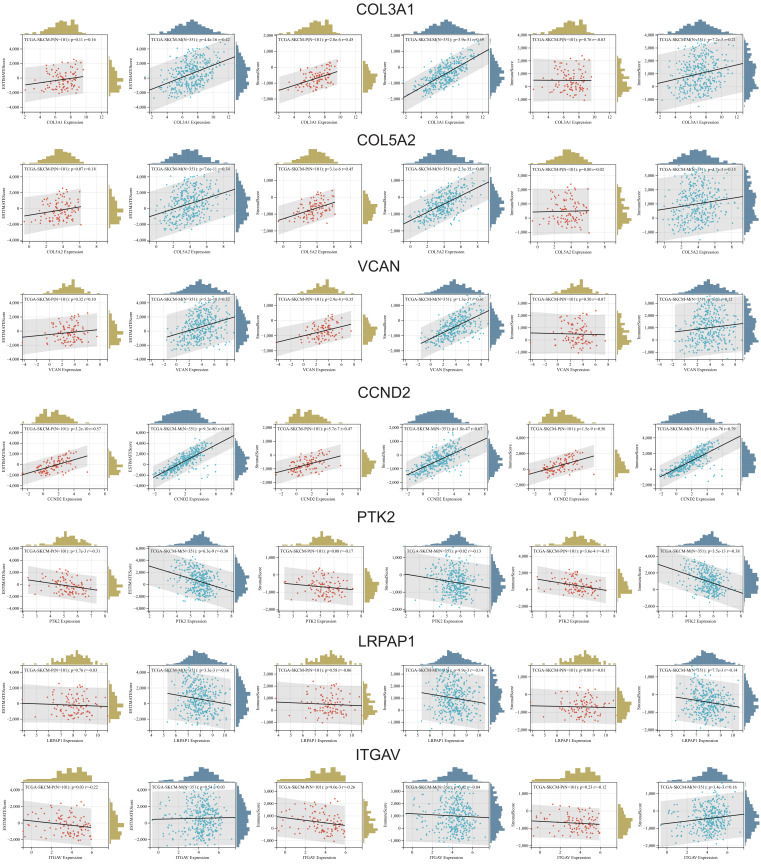
Spearman’s correlations between the expression of *COL3A1, CCND2, COL5A2*, *VCAN*, *PTK2, LRPAP1*, and *ITGAV* and the ESTIMATE, Stromal, and Immune scores in primary (red scatter plots) and metastatic (green scatter plots) skin melanoma.

Immune infiltration analysis using ESTIMATE (Estimation of STromal and Immune cells in MAlignant Tumor tissues using Expression data), immune, and stromal scores revealed key associations between ARGs and the tumor microenvironment (TME) in skin melanoma. *COL3A1*, *COL5A2*, *VCAN*, *CCND2*, *JAG2*, and *VTN* showed consistent positive correlations with all three scores in both primary and metastatic tumors, suggesting their involvement in enhancing stromal support and recruiting immune cells. These genes were particularly linked to stromal components and the overall ESTIMATE score, indicating a strong association with the tumor stroma and its role in promoting tumor progression. Interestingly, some gene-TME interactions varied between primary and metastatic samples, especially regarding stromal and immune scores. In contrast, *PTK2* and *LRPAP1* showed negative correlations with immune-related scores, pointing to a role in dampening immune infiltration and enabling immune evasion. *ITGAV* also exhibited negative correlations, particularly with the ESTIMATE and immune scores, hinting at a broader immunosuppressive effect that could hinder treatment response (Figure 6).

Overall, these findings highlight the complex role of ARGs in shaping the immune landscape of SKCM. Genes positively correlated with stromal and immune scores may serve as biomarkers to identify patients more likely to benefit from immunotherapy. In contrast, genes negatively associated with immune scores could be promising targets for therapies aimed at boosting immune cell infiltration and enhancing anti-tumor immune responses.

### 2.5. Immune Cell Analysis (CIBERSORT)

We also used CIBERSORT to investigate the relationship between ARG expression and immune cell infiltration in the TME of both primary and metastatic skin melanomas. *PTK2* showed strong positive correlations with activated T cells and M2 macrophages, and negative correlations with naïve and memory B cells, activated dendritic cells, eosinophils, and neutrophils across both tumor types (Figure S1). *PRG2* displayed distinct patterns—weakly positive in primary tumors and negative in metastatic ones—suggesting stage-specific immune interactions. In addition, *S100A4* was positively associated with M0 macrophages and negatively correlated with resting NK cells. *APOH* showed notable associations with CD8+ T cells and Tregs, indicating a broader role in immune modulation. *VAV2* correlated positively with M0 macrophages, pointing to a role in macrophage-driven responses. *ITGAV* and *SLCO2A1* were linked to multiple immune cell types, supporting their involvement in immune infiltration.

*CXCL6* was positively correlated with macrophages and negatively correlated with T cells, while *PGLYRP1* was associated with M1 macrophages, suggesting a role in pro-inflammatory responses. *COL3A1* and *VEGFA* were both positively associated with M0 macrophages and negatively with CD8+ T cells, implying a contribution to an immunosuppressive microenvironment. Together, these findings reveal the diverse and context-dependent roles of ARGs in modulating immune cell infiltration in melanoma.

We also identified complex and gene-specific associations between ARGs and immune cell populations in both primary and metastatic tumors. *FGFR1* showed a strong positive correlation with neutrophils, but negative correlations with dendritic cells and select macrophage subsets, suggesting it may influence immune suppression through targeted immune cell modulation. *FSTL1* was positively associated with T follicular helper cells and negatively with γδ T cells, pointing to a potential role in regulating T cell-mediated immunity. In contrast, *SERPINA5* correlated positively with activated dendritic cells and negatively with regulatory T cells, implying it may shape the immune microenvironment by enhancing antigen presentation while limiting immune suppression.

In fact, several genes displayed dual or context-dependent roles in immune modulation. *JAG1* was positively associated with macrophages and negatively with T cells, indicating a complex role in orchestrating immune infiltration. Similarly, *LRPAP1* and *KCNJ8* showed negative correlations with CD8+ T cells and positive correlations with macrophages and certain memory B cell subsets, suggesting that these genes may shift immune cell composition in ways that impact tumor immunity. *SPP1* and *TIMP1* followed a similar pattern—positively associated with macrophages and negatively with CD8+ T cells—supporting their potential role in promoting immune evasion via macrophage activation and T cell suppression.

Additional ARGs, including *PDGFA*, *VTN*, and *POSTN*, revealed intricate and subtype-specific correlations with both macrophages and T cells, reinforcing their involvement in shaping the tumor immune landscape. Notably, *APP*, *VCAN*, and *STC1* exhibited strong positive correlations with macrophages and activated NK cells, but negative associations with memory B and resting T cells. These inverse patterns may signal immunosuppressive effects that hinder effective antitumor responses in melanoma.

Collectively, these findings underscore the diverse immunomodulatory roles of ARGs and their influence on immune cell dynamics in the TME.

### 2.6. Immune Cell Analysis (xCELL)

To further explore ARG–immune cell interactions, we used xCELL to analyze correlations between ARG expression and immune cell populations in both primary and metastatic melanoma. *PTK2* expression showed a positive correlation with mast cells, but was negatively associated with CD8+ T cells, macrophages, and NK cells, suggesting a potential role in immune suppression across both tumor stages. *PRG2* displayed a similar pattern—positively correlated with CD4+ central memory T cells and negatively with immature dendritic cells. *S100A4* was positively linked to macrophages and regulatory T cells, while negatively associated with NK cells and eosinophils, highlighting its potential involvement in promoting immune evasion.

*VAV2* and *ITGAV* were associated with increased levels of activated T cells and macrophages, but showed negative correlations with B cells and CD8+ T cells, suggesting they may promote selective immune activation while suppressing cytotoxic responses. *SLCO2A1* was positively correlated with regulatory T cells and macrophages, and negatively with CD8+ T cells, reinforcing its possible immunosuppressive role (Figure S2).

*CXCL6* showed a strong positive correlation with immature dendritic cells and macrophages, but a negative correlation with CD8+ T cells, pointing to a dampening effect on cytotoxic immune activity. *PGLYRP1* revealed an intriguing profile—positively associated with innate immune cells like macrophages, but negatively with adaptive immune cells, indicating a potential role in balancing immune responses. *COL3A1* displayed diverse associations with various immune cell types, suggesting broad immunomodulatory effects. *VEGFA* correlated positively with M2 macrophages—a subtype linked to immunosuppressive activity—and negatively with T cells, further supporting its role in blunting anti-tumor immunity (Figure S3).

Together, these results underscore the intricate interplay between ARGs and immune effector cells in melanoma and they offer valuable insights into the immunological complexity of the tumor microenvironment.

In the metastatic tumors, *VEGFA* showed positive correlations with CD4+ T cells and M1 macrophages, but negative correlations with Tregs and B cells. In contrast, in the primary tumors, *VEGFA* was positively associated with endothelial cells and neutrophils, and negatively with NK cells, highlighting tumor stage-specific immune interactions. *PF4* positively correlated with dendritic cells and negatively with CD4+ T cells and B cells, indicating a potential role in skewing immune activation. *TNFRSF21* demonstrated complex associations: positive with Tregs and macrophages, but negative with NK cells and eosinophils, particularly in primary melanomas.

In the metastatic cohort, *JAG2* correlated positively with NKT cells and CD4+ memory T cells, while negatively with M2 macrophages, suggesting it may modulate both adaptive and immunosuppressive populations. *LRPAP1* showed weak positive correlations with macrophages and CD4+ T cells but was negatively associated with adipocytes. *MSX1* correlated with CD8+ T cells and M1 macrophages, whereas *NRP1* showed selective associations with M2 macrophages and other T cell subsets. *CCND2* consistently correlated positively with CD4+ central memory T cells across datasets, but showed inverse associations with other immune cell types (Figure S3).

Further analyses revealed distinct gene-specific roles in immune modulation. *NRP1* was positively correlated with macrophages and negatively with CD8+ T cells and NK cells, showing differing roles between metastatic and primary tumors. *VTN* showed positive associations with CD4+ T cells and dendritic cells in metastatic tumors, but negative correlations with NK cells and eosinophils. *PDGFA* correlated positively with macrophages and endothelial cells, but negatively with CD8+ T cells, suggesting a role in immune evasion. *LUM* expression was positively linked to smooth muscle cells and fibroblasts, and negatively to basophils in one dataset.

*THBD* showed positive correlations with M1 macrophages and negative with B cells. *TIMP1* was strongly associated with M2 macrophages and negatively with multiple T cell populations, indicating an immunosuppressive function. *SPP1* was positively linked to activated dendritic cells, and negatively to CD8+ T cells. OLR1 was positively correlated with macrophages, but negatively with both CD4+ and CD8+ T cells. *APP* showed positive associations with M2 macrophages, but inverse correlations with CD8+ T cells and T helper cells.

Arginine-regulated genes also showed significant relevance. *LPL* was positively correlated with macrophages, although its associations with other immune cells varied across datasets. *VCAN* consistently correlated positively with M2 macrophages and negatively with cytotoxic T cells, suggesting an immunosuppressive phenotype. *STC1* generally exhibited positive immune cell correlations, except for a strong negative correlation with neutrophils in metastatic samples.

Collectively, these results emphasize the intricate and multifaceted relationships between *ARG* expression and immune cell infiltration in melanoma.

### 2.7. Immune Cell Analysis (TIMER)

Comprehensive correlation analyses between immune cell populations and gene expression across both datasets revealed intricate and subtype-specific interactions. In the metastatic dataset, *PTK2* consistently showed significant negative correlations with multiple immune cell types, including B cells, CD4+ T cells, CD8+ T cells, neutrophils, and dendritic cells. In contrast, in the primary dataset, *PTK2* generally exhibited positive correlations, particularly with macrophages, suggesting divergent roles in immune regulation between primary and metastatic melanoma (Figure S4).

Similarly, *PRG2* demonstrated strong negative correlations with B cells in TCGA-SKCM-P, while showing positive associations with macrophages in both datasets. These patterns point to a potential role for *PRG2* in modulating innate immunity and suppressing adaptive immune responses (Figure S4).

Further analysis revealed gene-specific immune interactions that varied between primary and metastatic melanomas. In the TCGA-SKCM-M dataset, *S100A4* was positively correlated with CD8+ T cells and neutrophils, whereas in the primary dataset it was primarily associated with macrophages. *APOH* showed strong negative correlations with B cells and CD4+ T cells in the primary tumors, while correlating positively with macrophages in the metastatic ones. In addition, *VAV2* demonstrated significant positive associations with T cells and dendritic cells in the metastatic dataset but lacked notable positive correlations in the primary samples. *ITGAV* also displayed a mix of positive and negative associations, particularly involving T cells and macrophages in metastatic tumors. Lastly, *SLCO2A1* showed consistent positive correlations with most immune cell types in both datasets—except dendritic cells, where no correlation was observed.

Notably, *CXCL6* exhibited strong negative correlations with B and T cells in primary melanomas, while *PGLYRP1* was positively associated with neutrophils and macrophages in metastatic ones. *COL3A1* showed moderate positive correlations with a variety of immune cell types, with patterns that varied between datasets (Figure S5).

Deeper analysis of additional genes provided further insights. *COL3A1* displayed strong positive correlations with multiple immune cells in the TCGA-SKCM-M dataset, suggesting an immunoregulatory role. *COL5A2* showed variable associations, particularly with macrophages. Furthermore, *VEGFA* was consistently correlated with neutrophils in both datasets, while *PF4* had negative associations with several T cell subsets. In addition, *TNFRSF21* showed specific relevance to T cells and neutrophils, whereas *JAG2* had predominantly negative correlations, especially with T cells in the metastatic cohort. *FGFR1* was positively correlated with macrophages, and *FSTL1* showed associations with both T cells and macrophages in the TCGA-SKCM-P dataset. *JAG1* had strong positive correlations with macrophages and dendritic cells, underscoring its immunomodulatory potential. *SERPINA5* demonstrated strong positive associations with CD4+ T cells, supporting its relevance in immune-targeted therapeutic strategies (Figure S5).

Fixed correlation analysis in the TCGA-SKCM-M dataset revealed moderate but statistically significant positive correlations between *ARG* expression and several immune cell types, including B cells, CD4+ and CD8+ T cells, neutrophils, macrophages, and dendritic cells. In contrast, the TCGA-SKCM-P dataset showed more selective trends, primarily involving CD4+ and CD8+ T cells, while neutrophil associations were less prominent compared to the metastatic dataset (Figure S5).

### 2.8. Immune Cell Analysis (MCPcounter)

We used the MCPcounter algorithm as an additional way to determine the absolute abundance of various immune and stromal cell types in primary and metastatic melanomas, based on their ARG expression levels. We found that *PTK2* exhibited contrasting correlation patterns. In the TCGA-SKCM-M dataset, it was negatively associated with CD8+ T cells and other T cell subtypes, while in the TCGA-SKCM-P dataset, it showed positive correlations with dendritic cells, endothelial cells, and stronger associations with neutrophils. In addition, *PRG2* showed weak negative correlations with dendritic and endothelial cells in the primary tumors, but had no significant associations in the metastatic ones (Figure S6).

Furthermore, *S100A4* was negatively correlated with multiple immune cell types in the TCGA-SKCM-M dataset, but positively associated with macrophages in the TCGA-SKCM-P dataset. *APOH* also showed negative correlations with T cells in primary tumors and positive correlations with fibroblasts in metastatic ones. Genes such as *VAV2*, *ITGAV* and *SLCO2A1* demonstrated consistent positive associations with endothelial cells, macrophages and fibroblasts, while *CXCL6* and *PGLYRP1* showed strong positive correlations with neutrophils across both cohorts. *COL3A1* displayed robust positive correlations with a broad range of immune cells, reinforcing its role in the TME. Similar patterns were observed for *COL5A2* and *PF4*, though *PF4* showed contrasting correlations, with negative correlation with monocytes in primary tumors and positive ones with fibroblasts in metastatic ones. *JAG2* and *TNFRSF21* were correlated with dendritic cells and T cell subsets, while *FGFR1* showed strong positive correlations with cytotoxic T cells. *FSTL1*, *SERPINA5*, and *JAG1* also revealed significant correlations with immune cells including endothelial cells, fibroblasts and macrophages suggesting immunoregulatory potential.

Pearson correlation analysis confirmed these findings, with *JAG1* strongly correlating with endothelial and fibroblast cells, while *LRPAP1* showed positive correlations with endothelial cells and negative ones with CD8 T cells. *MSX1* and *CCND2* displayed opposing trends between datasets, underlining their context-specific roles. *NRP1* and *THBD* both demonstrated broad positive associations with immune subsets, suggesting a role in immune regulation. *KCNJ8* showed consistent positive correlations with T cells, NK cells and cytotoxic lymphocytes across datasets. *LPL* and *VCAN* correlated with neutrophils, fibroblasts and dendritic cells, highlighting their influence on the immune landscape. *STC1* and *VCAN* further showed strong correlations with NK cells, CD8 T cells and monocytic lineages, while T cells exhibited inverse correlations in both datasets (Figure S6).

Gene-specific correlations suggest diverse roles in immune modulation and tumor progression, offering potential targets for immunotherapy.

### 2.9. Immune Cell Profiling (EPIC)

We further used EPIC, which includes RNA-seq-based gene expression reference profiles from immune cells and other nonmalignant cell types found in tumors. During the assessment of *PTK2* expression in the TCGA-SKCM-M dataset, significant positive correlations with CD8 T cells and negative correlations with B cells, CAFs, and endothelial cells were found; whereas no significant correlations were observed in the TCGA-SKCM-P dataset. In addition, *PRG2* showed significant negative and positive correlations with CAFs and macrophages, respectively in primary tumors; while no significant correlations were seen in metastatic ones (Figure S7).

*S100A4* correlated positively with B cells, CD8 T cells and macrophages, but negatively with NK cells. In the case of *APOH* expression in CAFs, a significant negative correlation was seen only in the TCGA-SKCM-P dataset. *VAV2*, *ITGAV*, and *SLCO2A1* demonstrated positive correlations with multiple immune populations, including B cells, CAFs and endothelial cells. *CXCL6* was positively correlated with CAFs and CD4 T cells, while showing a negative correlation with CD8 T cells in the SKCM-M dataset. *PGLYRP1* correlated positively with B cells in the metastatic melanomas, but not in the primary ones (Figure S8).

Across both datasets, *COL3A1* and *COL5A2* were strongly associated with CAFs; while *COL3A1* showed negative correlations with CD8 T cells. *VEGFA* positively correlated with CD4 T cells in SKCM-P, but negatively with B cells in SKCM-M. *PF4*, *TNFRSF21*, and *JAG2* exhibited diverse correlations, notably with macrophages, T cells and endothelial cells. *FGFR1*, *FSTL1*, and *SERPINA5* displayed consistent positive correlations with CAFs, CD4 T cells and macrophages, respectively (Figure S8).

*JAG1*, *LRPAP1*, and *MSX1* correlated with CAFs and T cells particularly in metastatic cases. *CCND2* was broadly associated with immune populations, reinforcing its potential role in shaping the immune microenvironment. *NRP1*, *VTN*, and *PDGFA* revealed significant correlations with fibroblasts, macrophages and CD4 T cells, respectively. Furthermore, *LUM*, *THBD*, *OLR1*, and *KCNJ8* displayed distinctive immune signatures. Notably, *KCNJ8* showed strong positive correlations with CAFs and B cells, and weak negative ones with NK cells. *POSTN*, *APP* and *LPL* were also positively correlated with CAFs and CD4 T cells, while *VCAN* and *STC1* displayed consistent associations with CAFs, B cells and CD8 T cells, but were negatively correlated with NK cells (Figure S8). These findings underscore the intricate immune dynamics driven by ARG expression, highlighting subtype-specific immune modulation across melanoma stages.

### 2.10. Immune Cell Profiling (QUANTISEQ)

We then used quanTIseq, a deconvolution pipeline that quantifies the fractions and densities of 10 different immune cell types relevant for cancer immunology, as well as the proportion of uncharacterized cells (e.g., malignant cells in bulk tumors). We found that *PTK2* displayed consistent positive correlations with B cells, NK cells and monocytes, and negative correlations with CAFs and endothelial cells in metastatic melanomas. In addition, *PRG2* was positively correlated with T cells and monocytes, and negatively with M2 macrophages (Figure S9).

We also found that *S100A4* exhibited positive correlations with T cells and macrophages, but negative with monocytes. *APOH* was negatively correlated with regulatory T cells in primary melanomas, whereas *VAV2* was positively correlated with CD8 T cells, but negatively with dendritic cells. *ITGAV* was broadly associated with macrophages and inversely with T cells. *CXCL6*, *SLCO2A1* and *PGLYRP1* showed varied correlations: *CXCL6* positively associated with neutrophils, *SLCO2A1* with B cells and monocytes, and *PGLYRP1* with multiple immune types, suggesting broad immune involvement. *COL3A1*, *COL5A2* and *VEGFA* showed dataset-specific correlations; *COL3A1* had strong associations with M1 macrophages, while *VEGFA* was positively correlated with CD4 T cells in metastatic melanomas. Interestingly, *PF4* and *TNFRSF21* displayed contrasting roles, with *PF4* weakly correlating across immune types and *TNFRSF21* linking positively to B cells and M1 macrophages, but negatively to CD8 T cells. *FGFR1* had weak general correlations but a notable link with CD8 T cells. Furthermore, *FSTL1*, *SERPINA5* and *JAG1* correlated positively with macrophages, B cells and dendritic cells, and negatively with monocytes and CD4 T cells. Likewise, *LRPAP1, MSX1, CCND2*, and *NRP1* displayed strong associations with monocytes, Tregs and NK cells, especially in metastatic tumors. *VTN, PDGFA* and *LUM* also showed subtype-specific patterns with *PDGFA* strongly linked to macrophages and LUM to B cells. Other ARGs such as *TIMP1, SPP1, OLR1, KCNJ8* and *POSTN* demonstrated significant immune cell correlations. Notably *POSTN* showed opposing roles, with positive correlations with M1/M2 macrophages and negative correlations with NK cells and Tregs. *VCAN, APP, LPL* and *STC1* exhibited diverse associations, particularly in the TCGA-SKCM-M dataset, with *STC1* showing strong negative correlations with CD8 T cells and Tregs, but positive with NK cells and macrophages (Figure S10).

These findings underline the gene-specific and dataset-dependent immune interactions within the melanoma TME.

### 2.11. Immune Cell Profiling (IPS)

We then re-estimated key components of the tumor immune microenvironment, including the MHC (antigen presentation capacity), EC (effector cells), SC (immune suppressor cells), CP (immune checkpoints), AZ (antigen zones or areas of immune activation) and the IPS (Immunophenoscore) infiltration scores per patient and tumor using gene expression data, summarizing the tumor’s overall immunogenic profile.

We found that *PTK2* showed differing associations: in metastatic tumors, it correlated positively with MHC, SC and CP, and negatively with EC and AZ, while in primary tumors, it was positively correlated with MHC and SC, but its correlations with AZ were mixed. On the other hand, *PRG2* consistently showed negative correlations with multiple immune components, suggesting a suppressive role (Figure S11).

*S100A4* and *ITGAV* displayed dataset-specific patterns: negatively associated with SC and CP in metastatic melanomas, but positively with MHC and EC in primary ones. *APOH* was weakly negatively correlated overall but had a stronger positive correlation with SC in primary tumors. *VAV2* was broadly negatively correlated in metastatic tumors, but displayed limited significance in primary ones. *SLCO2A1* was positively correlated with EC, but negatively with SC and IPS in metastatic tumors. *CXCL6* showed strong negative associations with immune cells in the SKCM-M dataset, with negligible correlations in SKCM-P. *PGLYRP1* exhibited only weak associations across datasets. *COL3A1* was negatively associated with EC and CP, indicating possible roles in immune modulation. Several ARGs, such as *COL5A2* and *VEGFA* also demonstrated complex, context-specific correlations with immune cells. Notably, *VEGFA* was negatively associated with several immune types in SKCM-M, indicating a possible immunosuppressive effect (Figure S12).

*JAG2* displayed strong negative correlations with MHC and EC in both datasets, suggesting a consistent inhibitory role. *FSTL1* had negative correlations in SKCM-M, but variable trends on SKCM-P. *SERPINA5* exhibited inconsistent profiles between datasets. *JAG1, LRPAP1, MSX1, CCND2, NRP1, VTN, PDGFA, LUM, THBD*, and *TIMP1* each displayed unique patterns of association, often differing between metastatic and primary melanomas, underscoring the context-dependent nature of immune-gene interactions. Other ARGs, including *OLR1, KCNJ8, APP, POSTN*, and *STC1* showed significant yet heterogenous relationships with immune cells subsets, reinforcing the complexity of immune regulation in melanoma (Figure S12).

## 3. Discussion

Our comprehensive analysis of ARGs in skin cutaneous melanoma highlights their prognostic significance, molecular diversity, and immunological impact. By integrating gene expression profiles with clinical outcomes from both primary and metastatic tumor datasets, we identified distinct ARG expression patterns associated with overall survival, disease progression, and immune cell infiltration.

Several genes, including *S100A4*, *ITGAV*, and *COL3A1*, previously linked to tumor progression and poor prognosis [29,30,31,32,33], emerged in our study as robust prognostic indicators. These genes exhibited heterogeneous expression patterns and immune correlations across patient subgroups. Kaplan–Meier analyses confirmed significant associations between ARG expression and survival metrics, supporting their utility in patient stratification and prognostic modeling. Furthermore, differential expression analysis revealed upregulation of genes such as *PTK2*, *PRG2*, and *VEGFA* in tumor samples compared to normal skin, alongside downregulation of other ARGs—reinforcing their involvement in melanoma pathogenesis.

*VEGFA*, a well-characterized pro-angiogenic driver, is known to promote tumor vascularization and immune evasion [34,35,36,37,38,39]. Our findings confirm its central role in melanoma biology and suggest it remains a viable therapeutic target. Importantly, our results also underscore the immunomodulatory functions of ARGs. For example, *JAG1* and *SERPINA5*, which have been implicated in immune regulation and tumor-immune escape [33,40,41], showed complex associations with immune cell subsets—positively correlating with macrophages and negatively with cytotoxic T cells—indicating their potential involvement in immune evasion mechanisms. The divergent infiltration patterns observed between primary and metastatic tumors further emphasize the importance of tailoring immunotherapeutic approaches to tumor stage and immune context [42].

Leveraging advanced bioinformatics tools such as xCELL and TIMER enabled precise estimation of immune infiltration and detailed mapping of gene-immune interactions from bulk RNA-seq data [43,44]. These analyses revealed nuanced relationships between ARG expression and immune components. For instance, *CXCL6*, known for its role in neutrophil and macrophage recruitment [45,46], showed consistent immunomodulatory activity in our datasets. Similarly, *TNFRSF21* correlated positively with B cells and M1 macrophages in metastatic samples, while *JAG2* showed strong negative correlations with CD8+ T cells and dendritic cells. Previous studies suggest that *JAG2*, a Notch ligand, may contribute to immune evasion by impairing dendritic cell function and T cell priming [47,48,49], which aligns with our findings.

Together, these insights reveal the multifaceted roles of ARGs in melanoma progression and immune modulation. They provide a valuable foundation for future studies and therapeutic strategies aimed at targeting angiogenesis and immune pathways in a personalized and stage-specific manner.

Several additional genes—including *FSTL1*, *LRPAP1*, *MSX1*, and *APP*—exhibited distinct immune interaction profiles, suggesting diverse roles in modulating tumor–immune dynamics. Notably, *FSTL1* showed positive correlations with M2 macrophages in primary melanoma, aligning with prior studies that link it to M2 polarization in other cancers [50,51]. *SERPINA5* and *JAG1* were consistently negatively correlated with monocytes, indicating a potential role in immune suppression and myeloid cell regulation.

Other ARGs also demonstrated cell type-specific associations. For example, *POSTN* was positively correlated with neutrophils, whereas *LPL* and *VEGFA* were linked to distinct immune subsets. Importantly, *CAF-1* showed a negative correlation with CD8+ T cells, a pattern observed in earlier studies and confirmed in our data [52]. These findings underscore the multifaceted immunological effects of ARGs, reinforcing their dual role in regulating both angiogenesis and immune function.

Collectively, these immunogenomic insights highlight the potential of ARGs as actionable targets. Their expression patterns not only influence tumor vascularization, but also shape the immune microenvironment—affecting infiltration, immune suppression, and responsiveness to therapy. Modulating ARGs may enhance immune cell infiltration, boost responses to immune checkpoint inhibitors, and disrupt tumor-driven immune evasion mechanisms [27,53,54,55,56].

Despite the significance of these findings, certain limitations must be acknowledged. Our analyses rely heavily on TCGA data, which includes a limited number of normal samples and may introduce sampling bias. Furthermore, the study is based on transcriptomic data, and experimental validation is essential to confirm the functional roles of the identified ARGs. In addition, although our study is correlative, the patterns observed suggest mechanistic links such as STAT3-mediated PD-L1 expression driven by ARGs. Experimental validation using knockdown and overexpression models is needed to confirm these causal pathways.

To minimize tool-specific bias, we used seven immune deconvolution algorithms. While discrepancies (e.g., TIMER vs. MCPcounter for PTK2) were observed in our findings, consistent trends across multiple tools strengthen the robustness of our key findings.

Future research should use spatial transcriptomics (e.g., Visium) or co-culture assays with PBMCs to confirm the immunosuppressive roles of TIMP1, S100A4 and other ARGs. We believe that our findings support a rationale for clinical trials testing anti-angiogenic agents (e.g., Bevacizumab) in combination with anti-PD1 therapy (e.g., Pembrolizumab) in VEGFA-high melanoma patients.

Importantly, while our study is based on correlative analyses, emerging evidence suggests that ARGs may influence immune responses via signaling pathways such as STAT3, TGF-β, and NF-κB. For example, COL3A1 and S100A4 may modulate PD-L1 expression through stromal remodeling and inflammatory cytokine activation. Gene-editing techniques and spatial transcriptomics will be able to delineate causal mechanisms in the future. Additionally, single-cell RNA-seq or multiplex immunohistochemistry (mIHC) would be more appropriate for mapping ARG expression in tumor niches and cell-specific interactions. Future studies may incorporate melanoma cell lines or mouse models (e.g., B16-F10) with ARG modulation and immune profiling to evaluate functional roles. In addition, although we did not include a direct analysis of immunotherapy response cohorts the identified ARGs (e.g., TIMP1 and VEGFA) have been independently implicated in resistance to immune checkpoint blockade. Further validation using independent datasets would enhance translational potential.

Our findings support a dual functional model where ARGs such as S100A4, TIMP1, and VEGFA modulate not only angiogenesis, but also immune cell infiltration and checkpoint gene expression. This duality reinforces their relevance as both prognostic and potentially therapeutic biomarkers in melanoma.

In addition, survival analyses were based on gene expression alone, without stratification for mutational profiles, Tumor Mutational Burden (TMB), or treatment history, due to limited metadata availability in TCGA.

In particular, evaluating ARGs within the context of combination immunotherapy and anti-angiogenic strategies could uncover new therapeutic avenues [56,57,58,59,60]. Ultimately, integrating ARG expression profiles into clinical decision-making may support more precise, immune-informed treatment strategies for melanoma patients.

## 4. Materials and Methods

### 4.1. Data Collection and Processing

Thirty-six angiogenesis-related genes (ARGs) were obtained from the Hallmark gene sets of the MSigDB database, including: *PTK2*, *PRG2*, *S100A4*, *APOH*, *VAV2*, *ITGAV*, *SLCO2A1*, *CXCL6*, *PGLYRP1*, *COL3A1*, *COL5A2*, *VEGFA*, *PF4*, *TNFRSF21*, *JAG2*, *FGFR1*, *FSTL1*, *SERPINA5*, *JAG1*, *LRPAP1*, *MSX1*, *CCND2*, *NRP1*, *VTN*, *PDGFA*, *LUM*, *THBD*, *TIMP1*, *SPP1*, *OLR1*, *KCNJ8*, *POSTN*, *APP*, *LPL*, *VCAN*, and *STC1*. We determined the prognostic significance of these genes in skin melanoma and investigated their relationship with tumor immunity. We further extracted the expression data of 60 marker genes belonging to either inhibitory (24) or stimulatory (36) immune checkpoints, as described in Malta et al. [60].

### 4.2. Gene Expression Prognostic Analysis

We downloaded a standardized pan-cancer dataset from the UCSC Xena Browser (https://xenabrowser.net/; accessed on 15 February 2025): TCGA TARGET GTEx (PANCAN, N = 19,131, G = 60,499). Additionally, we collected the expression data of each of the 36 ARGs in each sample. We further screened samples from primary (TCGA-SKCM-P) and metastatic skin melanomas (TCGA-SKCM-M). In addition, we selected samples from ICGC/TCGA [61]. We also obtained a high-quality TCGA prognostic dataset, supplemented by TARGET follow-up data from the UCSC Cancer Browser (https://xenabrowser.net/datapages/; accessed on 15 February 2025) and excluded samples with a follow-up time of less than 30 days. We performed a log_2_(x + 0.001) transformation on each expression value and obtained expression data and the overall survival, disease-specific survival and progression-free interval data for samples from the TCGA-SKCM-P and TCGA-SKCM-M datasets. The disease-free interval data of samples from TCGA-SKCM-P and TCGA-SKCM-M was eliminated since there were less than 10 samples in each cancer type.

We also screened samples from normal solid tissue, primary solid tumor, primary tumor, normal tissue, primary blood derived cancer—bone marrow, and primary blood derived cancer—peripheral blood, and performed a log_2_(x + 0.001) transformation on each expression value. The tumor group consisted of 102 samples and the normal group consisted of 558 samples.

### 4.3. Tumor Immunity Analysis

#### 4.3.1. Immune Infiltration Analysis (ESTIMATE)

ESTIMATE (version 2.0.1.0) was used to analyze the expression data of each of the 36 ARGs in each sample [12]. We calculated stromal, immune and ESTIMATES score ratings for each patient in each tumor, based on gene expression. We used the corr.test function to calculate the Pearson and Spearman correlation coefficients between the genes’ expression and the immune infiltration score in each tumor. Finally, we observed whether gene expression was significantly correlated with immune infiltration in the two skin melanoma datasets (TCGA-SKCM-P and TCGA-SKCM-M).

#### 4.3.2. Tumor Immunity Analysis—Immune Cell Analysis (CIBERSORT)

We used the package IOBR (version 1.0.0.001) [13], deconvo_CIBERSORT method [62], to calculate the infiltration scores of B_cells_naive, B_cells_memory, Plasma_cells, T_cells_CD8, T_cells_CD4_naive, T_cells_CD4_memory_resting, T_cells_CD4_memory_activated, T_cells_follicular_helper, T_cells_regulatory_(Tregs), T_cells_gamma_delta, NK_cells_resting, NK_cells_activated, Monocytes, Macrophages_M0, Macrophages_M1, Macrophages_M2, Dendritic_cells_resting, Dendritic_cells_activated, Mast_cells_resting, Mast_cells_activated, Eosinophils, and Neutrophils for each patient in each tumor based on gene expression.

We obtained 22 types of immune cell infiltration scores of the tumor samples in the TCGA-SKCM-P and TCGA-SKCM-M datasets. We used the corr.test function of the R package psych (version 2.1.6) to calculate the Pearson and Spearman correlation coefficients between genes and immune cell infiltration scores in each tumor to determine the significantly correlated immune infiltration scores in TCGA-SKCM-P and TCGA-SKCM-M.

#### 4.3.3. Tumor Immunity Analysis—Immune Cell Analysis (xCELL)

The deconvo_xCell method [63] was used to re-evaluate the aDCs, Adipocytes, Astrocytes, B-cells, Basophils, CD4+_memory_T-cells, CD4+_naive_T-cells, CD4+_T-cells, CD4+_Tcm, CD4+_Tem, CD8+_naive_T-cells, CD8+_T-cells in each patient in each tumor based on gene expression. s, CD8+_Tcm, CD8+_Tem, cDC, Chondrocytes, Class-switched_memory_B-cells, CLP, CMP, DC, Endothelial_cells, Eosinophils, Epithelial_cells, Erythrocytes, Fibroblasts, GMP, Hepatocytes, HSC, iDC, Keratinocytes, ly_Endothelial_cel ls, Macrophages, Macrophages_M1, Macrophages_M2, Mast_cells, Megakaryocytes, Melanocytes, Memory_B-cells, MEP, Mesangial_cells, Monocytes, MPP, MSC, mv_Endothelial_cells, Myocytes, naive_B-cells, Neurons, Neutrophils, NK_cel ls, NKT, Osteoblast, pDC, Pericytes, Plasma_cells, Platelets, Preadipocytes, pro_B-cells, Sebocytes, Skeletal_muscle, Smooth_muscle, Tgd_cells, Th1_cells, Th2_cells, Tregs, Immune score, Stroma score, and Microenvironment score.

We obtained 67 types of immune cell infiltration scores of the tumor samples in the TCGA-SKCM-P and TCGA-SKCM-M datasets. We used the corr.test function of the R package psych (version 2.1.6) to calculate the Pearson and Spearman correlation coefficients between genes and immune cell infiltration scores in each tumor to determine the significantly correlated immune infiltration scores in each dataset.

#### 4.3.4. Tumor Immunity Analysis—Immune Cell Analysis (TIMER)

The Timer method [64] based on gene expression was used to re-evaluate the B cell, T cell CD4, T cell CD8, neutrophil, macrophage, and DC infiltration scores in each patient in each tumor. We obtained six types of immune cell infiltration scores of the tumor samples in the two datasets (TCGA-SKCM-P and TCGA-SKCM-M). We used the corr.test function of the R package psych (version 2.1.6) to calculate the Pearson and Spearman correlation coefficients between genes and immune cell infiltration scores in each tumor to determine the significantly correlated immune infiltration scores in the two datasets.

#### 4.3.5. Tumor Immunity Analysis—Immune Cell Analysis (MCPcounter)

The deconvo_mcpcounter method [65] was used to re-evaluate the infiltration scores of T_cells, CD8_T_cells, Cytotoxic_lymphocytes, B_lineage, NK_cells, Monocytic_lineage, Myeloid_dendritic_cells, Neutrophils, Endothelial_cells, and Fibroblasts in each patient in each tumor based on gene expression. We obtained 10 types of immune cell infiltration scores of the tumor samples in both datasets. The corr.test function of the R package psych (version 2.1.6) was used to calculate the correlation between genes and immune cell infiltration scores in each tumor as previously mentioned.

#### 4.3.6. Tumor Immunity Analysis—Immune Cell Profiling (EPIC)

The deconvo_epic method [66] was used to re-evaluate the infiltration scores of Bcells, CAFs, CD4_Tcells, CD8_Tcells, Endothelial, Macrophages, NKcells, and other cells in each patient in each tumor based on gene expression. We obtained 8 types of immune cell infiltration scores of the tumor samples in both datasets. The corr.test function of the R package psych (version 2.1.6) was used to calculate the correlation between genes and immune cell infiltration scores in each tumor as previously mentioned.

#### 4.3.7. Tumor Immunity Analysis—Immune Cell Profiling (QUANTISEQ)

The deconvo_quantiseq method [67] was used to re-evaluate the infiltration scores of B_cells, macrophages_M1, macrophages_M2, monocytes, neutrophils, NK_cells, T_cells_CD4, T_cells_CD8, Tregs, Dendritic_cells, and other in each patient in each tumor based on gene expression. We obtained 11 types of immune cell infiltration scores of the tumor samples in the two datasets. We used the corr.test function of the R package psych (version 2.1.6) to calculate the Pearson and Spearman correlation coefficients between genes and immune cell infiltration scores in each tumor to determine the significantly correlated immune infiltration scores in TCGA-SKCM-P and TCGA-SKCM-M.

#### 4.3.8. Tumor Immunity Analysis—Immune Cell Profiling (IPS)

The deconvo_ips method [67,68,69], re-estimated the MHC, EC, SC, CP, AZ, and IPS infiltration scores of each patient in each tumor based on gene expression.

We obtained 6 types of immune cell infiltration scores of the tumor samples in TCGA-SKCM-P and TCGA-SKCM-M. The corr.test function of the R package psych (version 2.1.6) was used to calculate the correlation between genes and immune cell infiltration scores in each tumor as previously mentioned.

### 4.4. Statistical Analysis

We used R software (version 3.6.4) to calculate the expression difference between normal skin samples and skin melanomas and used unpaired Wilcoxon Rank Sum and Signed Rank Tests for differential significance analysis. The expression difference was considered statistically significant if the *p* ≤ 0.05. We also used the coxph function to establish the Cox proportional hazards regression model [70] to analyze the relationship between gene expression and prognosis in each tumor. We used the Logrank statistical test to obtain the prognostic significance in the two datasets.

Furthermore, we used maxstat (maximally selected rank statistics with several *p*-value approximations version: 0.7–25) to calculate the optimal cutoff value of each of the 36 ARGs, setting the minimum number of grouped samples to be >25% and the maximum number of grouped samples to be <75%. Based on the optimal cutoff value, the patients were divided into two groups, high and low. The survfit function of the R package survival was further used to analyze the prognostic difference between the two groups. The logrank test was used to evaluate the significance of the prognostic differences between the samples of different groups. Finally, we observed whether the difference was significant or not. The difference was considered statistically significant if the *p* ≤ 0.05.

## 5. Conclusions

Our study suggests the use of angiogenesis-related genes not merely as vascular regulators, but as immune modulators as well. Among 36 such genes, the strongest prognostic and immunological associations were observed for *S100A4*, *TIMP1*, *ITGAV, VEGFA*, and *COL3A1*, which we highlight as primary candidates for future functional validation. Overall, our study sheds lights on the strong impact of the angiogenesis-related genes in the prognosis, molecular features, and immune responses of skin melanoma.

## Data Availability

Genomic data were extracted from TCGA (https://portal.gdc.cancer.gov/, accessed on 1 May 2024). The R scripts used for immune deconvolution are all available here: ESTIMATE: https://bioinformatics.mdanderson.org/estimate/rpackage.html (accessed on 15 February 2025); CIBERSORTx: https://cibersortx.stanford.edu/ (accessed on 10 January 2025); Immune cell analysis (xCELL): https://github.com/dviraran/xCell (accessed on 15 February 2025); TIMER2.0: http://timer.cistrome.org (accessed on 15 February 2025).

## Supplementary Materials

The supporting information can be downloaded at: https://www.mdpi.com/article/10.3390/ijms26178254/s1.

## Abbreviations

ARGs	Angiogenesis-related genes
UVR	Ultraviolet radiation
TME	Tumor microenvironment
SKCM-P	Primary skin cutaneous melanoma
SKCM-M	Metastatic skin cutaneous melanoma
SKCM	Skin cutaneous melanoma
MHC	Major histocompatibility complex
EC	Effector cells
SC	Immune suppressor cells
CP	Immune checkpoints
AZ	Antigen zones or areas of immune activation
IPS	Immunophenoscore
Tregs	Regulatory T cells
Tcm	CD4+ central memory T cells
mIHC	Multiplex immunohistochemistry
TMB	Tumor mutational burden

## References

[1] Shain A.H., Bastian B.C. (2016). From melanocytes to melanomas. Nat. Rev. Cancer.

[2] Centeno P.P., Pavet V., Marais R. (2023). The journey from melanocytes to melanoma. Nat. Rev. Cancer.

[3] Slominski A., Tobin D.J., Shibahara S., Wortsman J. (2004). Melanin pigmentation in mammalian skin and its hormonal regulation. Physiol. Rev..

[4] Slominski R.M., Sarna T., Płonka P.M., Raman C., Brożyna A.A., Slominski A.T. (2022). Melanoma, Melanin, and Melanogenesis: The Yin and Yang Relationship. Front. Oncol..

[5] Slominski R.M., Kim T.-K., Janjetovic Z., Brożyna A.A., Podgorska E., Dixon K.M., Mason R.S., Tuckey R.C., Sharma R., Crossman D.K. (2024). Malignant Melanoma: An Overview, New Perspectives, and Vitamin D Signaling. Cancers.

[6] Yang K., Oak A.S.W., Slominski R.M., Brożyna A.A., Slominski A.T. (2020). Current molecular markers of melanoma and treatment targets. Int. J. Mol. Sci..

[7] Teixido C., Castillo P., Martinez-Vila C., Arance A., Alos L. (2021). Molecular markers and targets in melanoma. Cells.

[8] Leonardi G.C., Falzone L., Salemi R., Zanghì A., Spandidos D.A., Mccubrey J.A., Candido S., Libra M. (2018). Cutaneous melanoma: From pathogenesis to therapy (Review). Int. J. Oncol..

[9] Roky A.H., Islam M.M., Ahasan A.M.F., Mostaq M.S., Mahmud M.Z., Amin M.N., Mahmud M.A. (2024). Overview of skin cancer types and prevalence rates across continents. Cancer Pathog. Ther..

[10] Wang M., Gao X., Zhang L. (2025). Recent global patterns in skin cancer incidence, mortality, and prevalence. Chin. Med. J..

[11] Lipsker D., Engel F., Cribier B., Velten M., Hedelin G. (2007). Trends in melanoma epidemiology suggest three different types of melanoma. Br. J. Dermatol..

[12] Long G.V., Swetter S.M., Menzies A.M., Gershenwald J.E., Scolyer R.A. (2023). Cutaneous melanoma. Lancet.

[13] Schadendorf D., van Akkooi A.C.J., Berking C., Griewank K.G., Gutzmer R., Hauschild A., Stang A., Roesch A., Ugurel S. (2015). Melanoma. Nat. Rev. Dis. Primers.

[14] Hanahan D., Weinberg R.A. (2011). Hallmarks of cancer: The next generation. Cell.

[15] Baeriswyl V., Christofori G. (2009). The angiogenic switch in carcinogenesis. Semin. Cancer Biol..

[16] Cazzato G., Ingravallo G., Ribatti D. (2024). Angiogenesis Still Plays a Crucial Role in Human Melanoma Progression. Cancers.

[17] Srivastava A., Ralhan R., Kaur J. (2003). Angiogenesis in cutaneous melanoma: Pathogenesis and clinical implications. Microsc. Res. Tech..

[18] Baluk P., Hashizume H., McDonald D.M. (2005). Cellular abnormalities of blood vessels as targets in cancer. Curr. Opin. Genet. Dev..

[19] Mahabeleshwar G.H., Byzova T.V. (2007). Angiogenesis in Melanoma. Semin. Oncol..

[20] Rahma O.E., Hodi F.S. (2019). The intersection between tumor angiogenesis and immune suppression. Clin. Cancer Res..

[21] McKeage M.J., Baguley B.C. (2010). Disrupting established tumor blood vessels: An emerging therapeutic strategy for cancer. Cancer.

[22] Simiczyjew A., Dratkiewicz E., Mazurkiewicz J., Ziętek M., Matkowski R., Nowak D. (2020). The influence of tumor microenvironment on immune escape of melanoma. Int. J. Mol. Sci..

[23] Falcone I., Conciatori F., Bazzichetto C., Ferretti G., Cognetti F., Ciuffreda L., Milella M. (2020). Tumor microenvironment: Implications in melanoma resistance to targeted therapy and immunotherapy. Cancers.

[24] Li C., Kuai L., Cui R., Miao X. (2022). Melanogenesis and the Targeted Therapy of Melanoma. Biomolecules.

[25] Wang W., He W., Guo L. (2021). Signal pathways of melanoma and targeted therapy. Signal Transduct. Target. Ther..

[26] Jour G., Ivan D., Aung P.P. (2016). Angiogenesis in melanoma: An update with a focus on current targeted therapies. J. Clin. Pathol..

[27] Zhou X., Ni Y., Liang X., Lin Y., An B., He X., Zhao X. (2022). Mechanisms of Tumor Resistance to Immune Checkpoint Blockade and Combination Strategies to Overcome Resistance. Front. Immunol..

[28] Loges S., Schmidt T., Carmeliet P. (2010). Mechanisms of Resistance to Anti-Angiogenic Therapy and Development of Third-Generation Anti-Angiogenic Drug Candidates. Genes Cancer.

[29] Miskolczi Z., Smith M.P., Rowling E.J., Ferguson J., Barriuso J., Wellbrock C. (2018). Collagen Abundance Controls Melanoma Phenotypes through Lineage-Specific Microenvironment Sensing. Oncogene.

[30] Nurzat Y., Su W., Min P., Li K., Xu H., Zhang Y. (2021). Identification of Therapeutic Targets and Prognostic Biomarkers Among Integrin Subunits in the Skin Cutaneous Melanoma Microenvironment. Front. Oncol..

[31] Xiong T., Pan F., Li D. (2019). Expression and Clinical Significance of S100 Family Genes in Patients with Melanoma. Melanoma Res..

[32] Maelandsmo G.M., Flørenes V.A., Mellingsaeter T., Hovig E., Kerbel R.S., Fodstad Ø. (1997). Differential Expression Patterns of S100a2, S100a4 and S100a6 During Progression of Human Malignant Melanoma. Int. J. Cancer.

[33] Ilkovitch D., Lopez D.M. (2008). Immune Modulation by Melanoma-Derived Factors. Exp. Dermatol..

[34] Ferrara N. (2002). VEGF and the Quest for Tumour Angiogenesis Factors. Nat. Rev. Cancer.

[35] Claesson-Welsh L., Welsh M. (2013). VEGFA and Tumour Angiogenesis. J. Intern. Med..

[36] Malekan M., Haass N.K., Rokni G.R., Gholizadeh N., Ebrahimzadeh M.A., Kazeminejad A. (2024). VEGF/VEGFR Axis and Its Signaling in Melanoma: Current Knowledge toward Therapeutic Targeting Agents and Future Perspectives. Life Sci..

[37] Rajabi P., Neshat A., Mokhtari M., Rajabi M.A., Eftekhari M., Tavakoli P. (2012). The role of VEGF in melanoma progression. J. Res. Med. Sci..

[38] Ferrara N. (2009). Vascular Endothelial Growth Factor. Arterioscler. Thromb. Vasc. Biol..

[39] Ziyad S., Iruela-Arispe M.L. (2011). Molecular Mechanisms of Tumor Angiogenesis. Genes Cancer.

[40] Meng L., Zhang C., Yu P. (2024). Treating Cancer through Modulating Exosomal Protein Loading and Function: The Prospects of Natural Products and Traditional Chinese Medicine. Pharmacol. Res..

[41] Meng J., Jiang Y.-Z., Zhao S., Tao Y., Zhang T., Wang X., Zhang Y., Sun K., Yuan M., Chen J. (2022). Tumor-Derived Jagged1 Promotes Cancer Progression through Immune Evasion. Cell Rep..

[42] Gajewski T.F. (2011). Molecular Profiling of Melanoma and the Evolution of Patient-Specific Therapy. Semin. Oncol..

[43] Liu Y., Wang L., Ngan H.-L., Lui V.W.Y. (2020). Bioinformatics Tools and Validation Methods for Immune Infiltrates in Tumor Microenvironments and Immunogenomics. Recent Trends in Biotechnology.

[44] Desta G.M., Birhanu A.G. (2025). Advancements in Single-Cell RNA Sequencing and Spatial Transcriptomics: Transforming Biomedical Research. Acta Biochim. Pol..

[45] Dai C.-L., Yang H.-X., Liu Q.-P., Rahman K., Zhang H. (2023). CXCL6: A Potential Therapeutic Target for Inflammation and Cancer. Clin. Exp. Med..

[46] SenGupta S., Hein L.E., Parent C.A. (2021). The Recruitment of Neutrophils to the Tumor Microenvironment Is Regulated by Multiple Mediators. Front. Immunol..

[47] Monticone G., Miele L. (2021). Notch Pathway: A Journey from Notching Phenotypes to Cancer Immunotherapy. Notch Signaling in Embryology and Cancer.

[48] Wang M., Yu F., Zhang Y., Li P. (2024). Novel Insights into Notch Signaling in Tumor Immunity: Potential Targets for Cancer Immunotherapy. Front. Immunol..

[49] Li D., Masiero M., Banham A.H., Harris A.L. (2014). The Notch Ligand Jagged1 as a Target for Anti-Tumour Therapy. Front. Oncol..

[50] Li L., Huang S., Yao Y., Chen J., Li J., Xiang X., Deng J., Xiong J. (2020). Follistatin-like 1 (FSTL1) is a prognostic biomarker and correlated with immune cell infiltration in gastric cancer. World J. Surg. Oncol..

[51] Zhou F., Chen Q., Yang M., Wang W., Huang Y., Zeng Z. (2025). FSTL1 Sustains Glioma Stem Cell Stemness and Promotes Immunosuppressive Macrophage Polarization in Glioblastoma. Cancer Lett..

[52] Wasik A., Ratajczak-Wielgomas K., Badzinski A., Dziegiel P., Podhorska-Okolow M. (2022). The Role of Periostin in Angiogenesis and Lymphangiogenesis in Tumors. Cancers.

[53] Fujiwara Y., Mittra A., Naqash A.R., Takebe N. (2020). A Review of Mechanisms of Resistance to Immune Checkpoint Inhibitors and Potential Strategies for Therapy. Cancer Drug Resist..

[54] Wu Z., Liu X., Yan Z., Sun Y., Zhang M., Lin Y. (2022). The Role of Angiogenesis in Melanoma: Clinical Treatments and Future Expectations. Front. Pharmacol..

[55] Lugano R., Ramachandran M., Dimberg A. (2020). Tumor Angiogenesis: Causes, Consequences, Challenges and Opportunities. Cell. Mol. Life Sci..

[56] Comunanza V., Bussolino F. (2017). Therapy for Cancer: Strategy of Combining Anti-Angiogenic and Target Therapies. Front. Cell Dev. Biol..

[57] Flaherty K.T. (2023). A Twenty Year Perspective on Melanoma Therapy. Pigment Cell Melanoma Res..

[58] Algazi A.P., Soon C.W., Daud A.I. (2010). Treatment of Cutaneous Melanoma: Current Approaches and Future Prospects. Cancer Manag. Res..

[59] Dhanyamraju P.K., Patel T.N. (2022). Melanoma Therapeutics: A Literature Review. J. Biomed. Res..

[60] Thorsson V., Gibbs D.L., Brown S.D., Wolf D., Bortone D.S., Ou Yang T.-H., Porta-Pardo E., Gao G.F., Plaisier C.L., Eddy J.A. (2018). The immune landscape of cancer. Immunity.

[61] ICGC/TCGA Pan-Cancer Analysis of Whole Genomes Consortium (2020). Pan-cancer analysis of whole genomes. Nature.

[62] Newman A.M., Liu C.L., Green M.R., Gentles A.J., Feng W., Xu Y., Hoang C.D., Diehn M., Alizadeh A.A. (2015). Robust enumeration of cell subsets from tissue expression profiles. Nat. Methods.

[63] Aran D., Hu Z., Butte A.J. (2017). xCell: Digitally portraying the tissue cellular heterogeneity landscape. Genome Biol..

[64] Li T., Fan J., Wang B., Traugh N., Chen Q., Liu J.S., Li B., Liu X.S. (2017). TIMER: A web server for comprehensive analysis of tumor-infiltrating immune cells. Cancer Res..

[65] Becht E., Giraldo N.A., Lacroix L., Buttard B., Elarouci N., Petitprez F., Selves J., Laurent-Puig P., Sautès-Fridman C., Fridman W.H. (2016). Estimating the population abundance of tissue-infiltrating immune and stromal cell populations using gene expression. Genome Biol..

[66] Racle J., de Jonge K., Baumgaertner P., Speiser D.E., Gfeller D. (2017). Simultaneous enumeration of cancer and immune cell types from bulk tumor gene expression data. eLife.

[67] Finotello F., Mayer C., Plattner C., Laschober G., Rieder D., Hackl H., Krogsdam A., Loncova Z., Posch W., Wilflingseder D. (2019). Molecular and pharmacological modulators of the tumor immune contexture revealed by deconvolution of RNA-seq data. Genome Med..

[68] Charoentong P., Finotello F., Angelova M., Mayer C., Efremova M., Rieder D., Hackl H., Trajanoski Z. (2017). Pan-cancer immunogenomic analyses reveal genotype-immunophenotype relationships and predictors of response to checkpoint blockade. Cell Rep..

[69] Zeng D., Ye Z., Shen R., Yu G., Wu J., Xiong Y., Zhou R., Qiu W., Huang N., Sun L. (2021). IOBR: Multi-omics immuno-oncology biological research to decode tumor microenvironment and signatures. Front. Immunol..

[70] Andersen P.K., Gill R.D. (1982). Cox’s regression model for counting processes: A large sample study. Ann. Stat..

