# Supplementary material for: Angiogenesis-Related Genes Predict Outcomes and Immune Traits in Skin Melanoma
Source: Int J Mol Sci. 2025 Aug 26;26(17):8254. doi: 10.3390/ijms26178254 (PMC12428775; doi:10.3390/ijms26178254)
Supplement: Supplementary file 1 [file ijms-26-08254-s001.zip › ijms-3684338-supplementary.pdf]

Supplementary Figures.

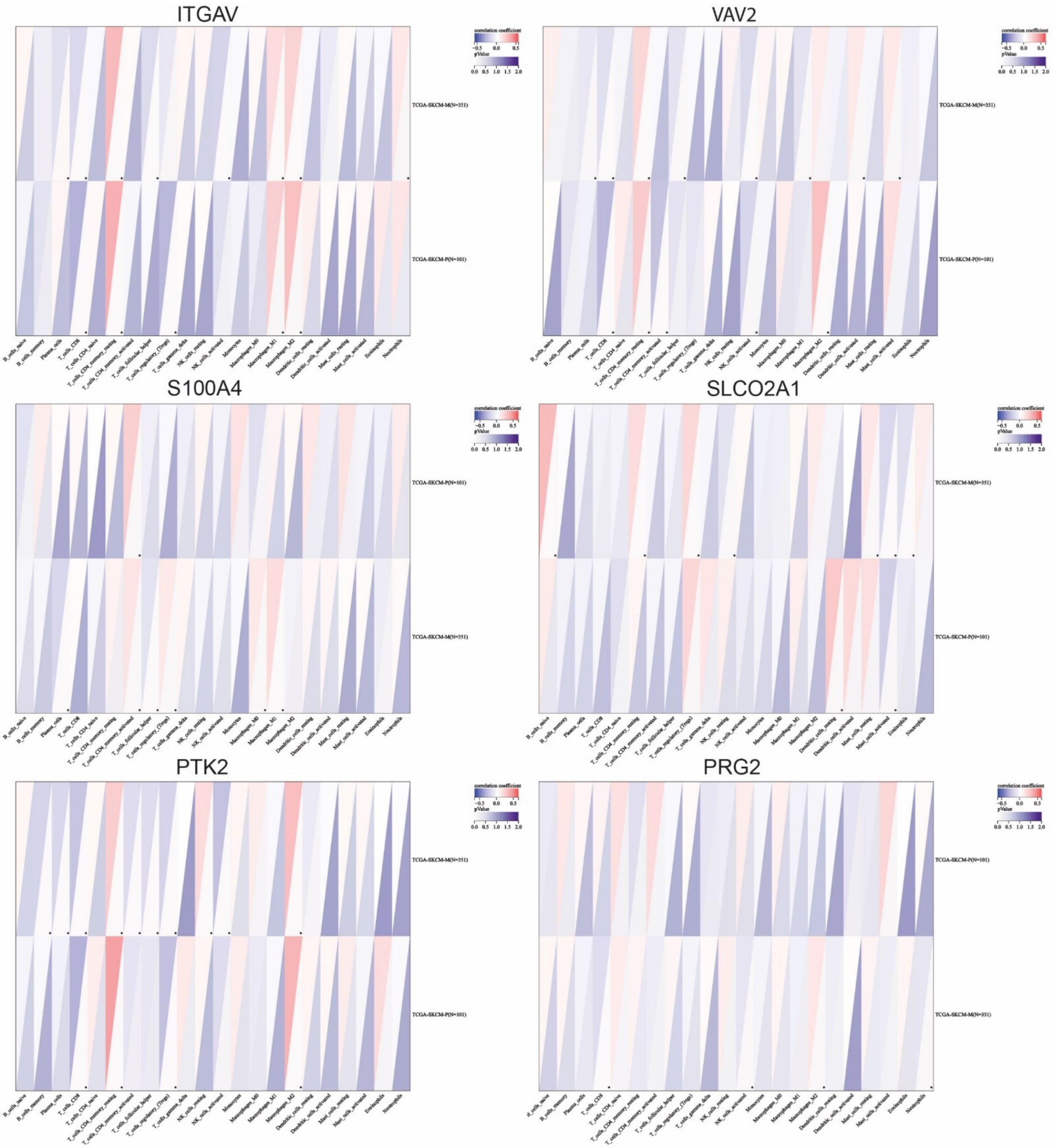

Figure S1 Spearman's correlations between the expression of *ITGAV, VAV2, S100A4, SLCO2A1, PTK2,* and *PRG2,* and the infiltration in different immune cells in primary and metastatic skin melanoma, using CIBERSORT. Asterisks denote statistical significance: *, p<0.05; ****, p<0.001.

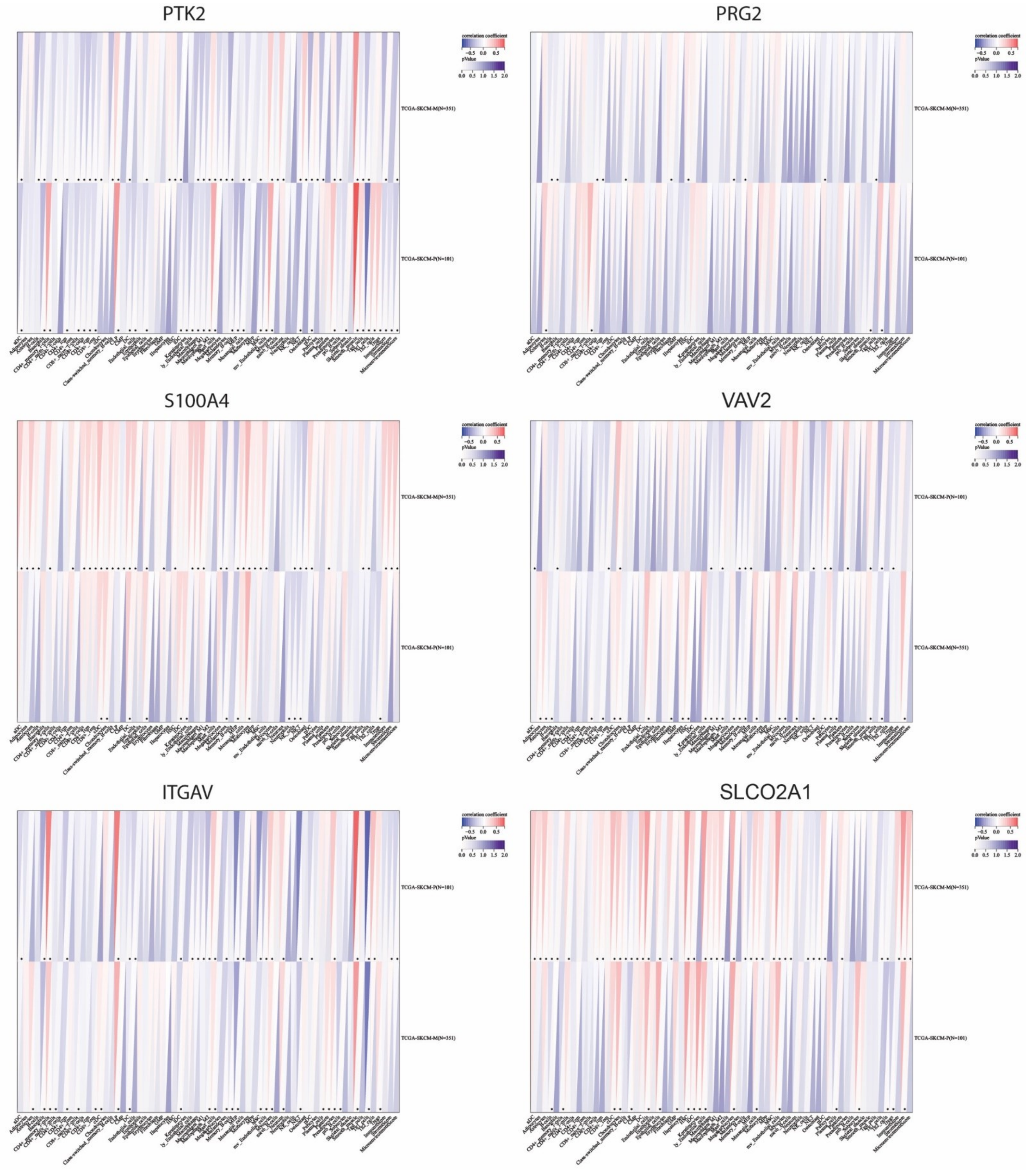

Figure S2. Spearman's correlations between the expression of *PTK2, PRG2, S100A4, VAV2, ITGAV* and *SLCO2A1,* and the infiltration in different immune cells in primary and metastatic skin melanoma, using xCELL. Asterisks denote statistical significance: *, p<0.05; ****, p<0.001.

APOH

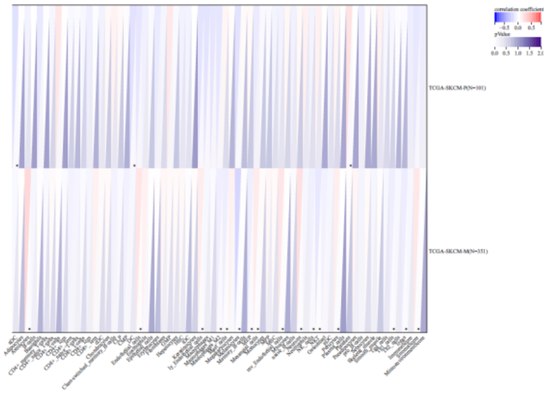

CCND2

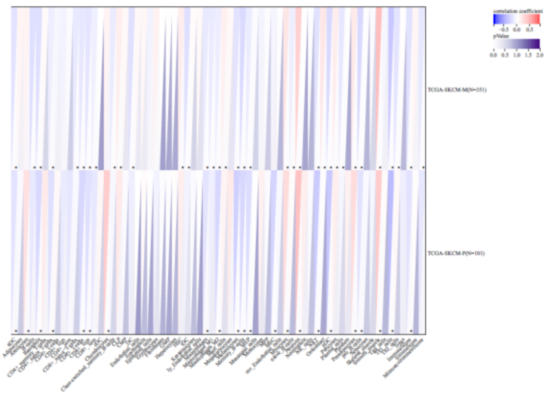

APP

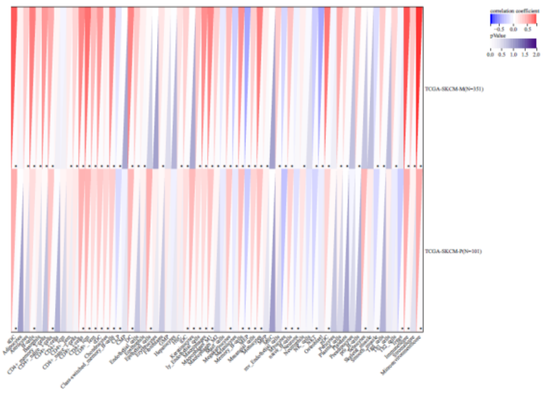

COL3A1

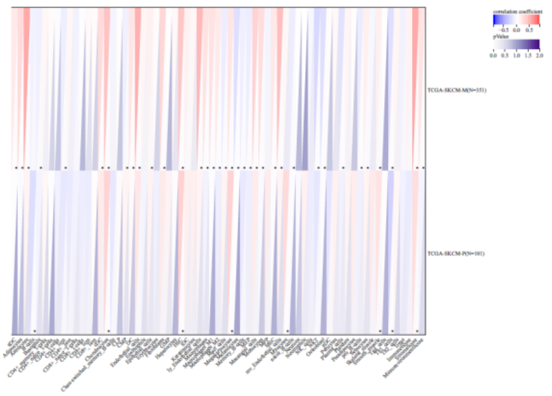

COL5A2

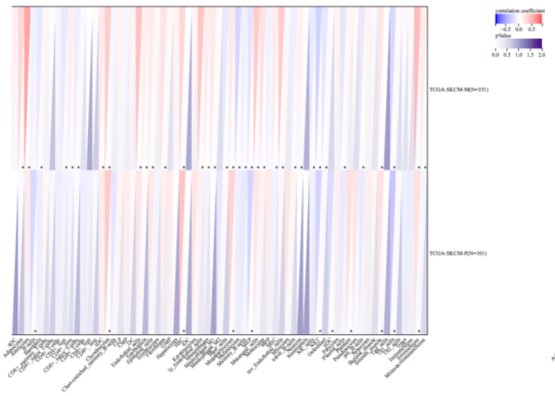

CXCL6

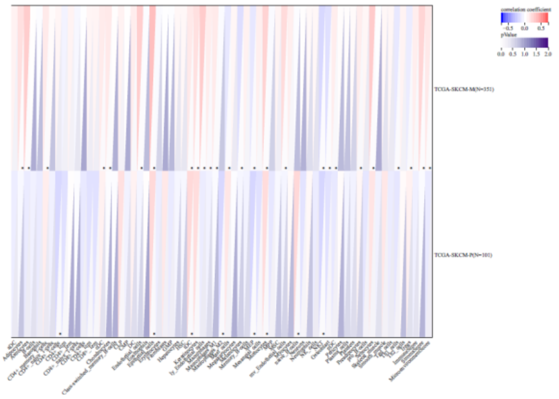

FGFR1

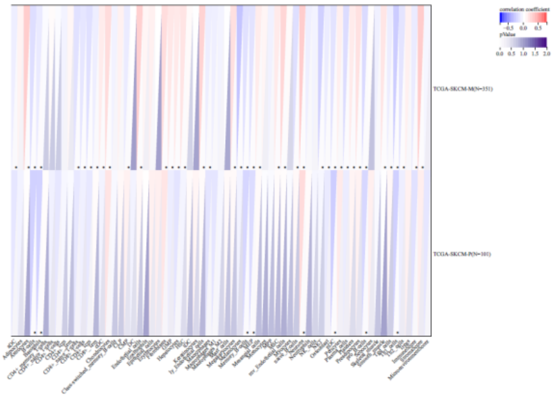

FSTL1

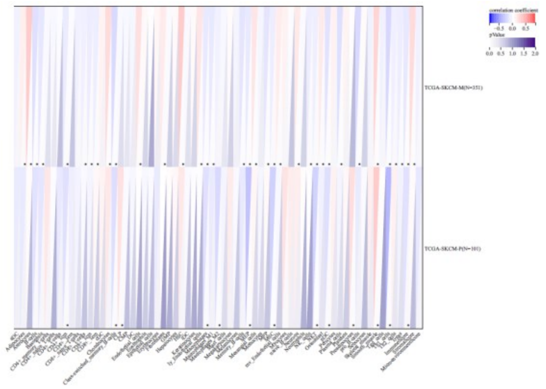

ITGAV

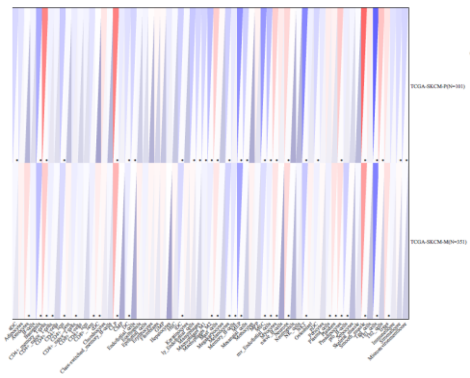

JAG1

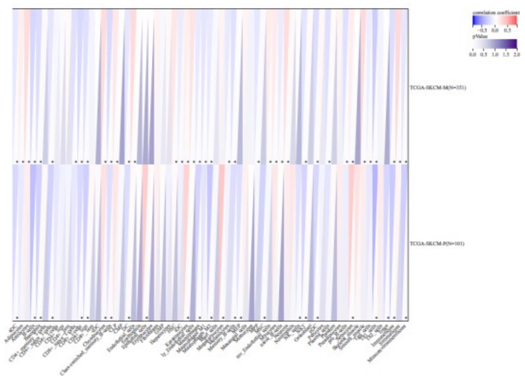

JAG2

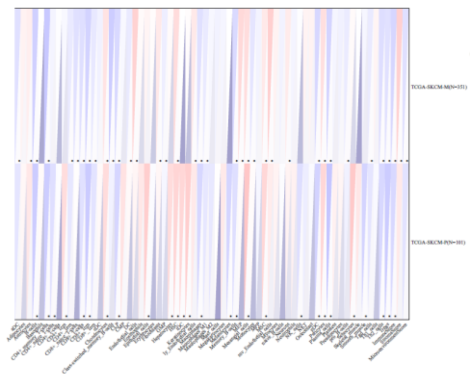

KCNJ8

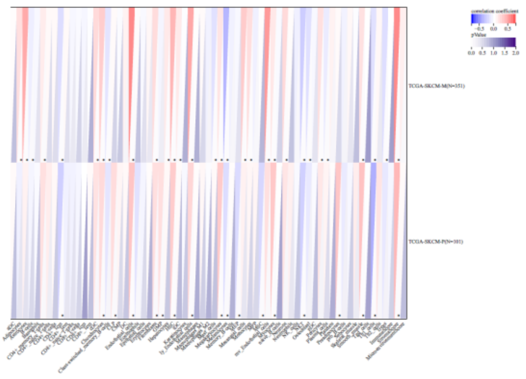

LPL

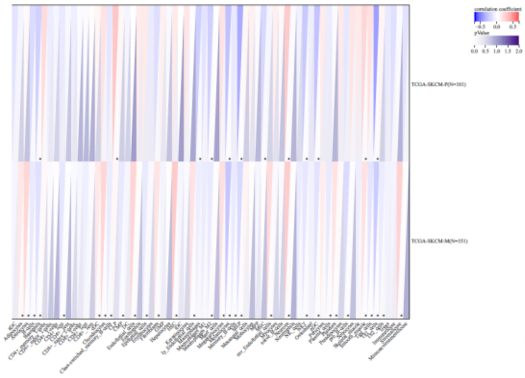

LRPAP1

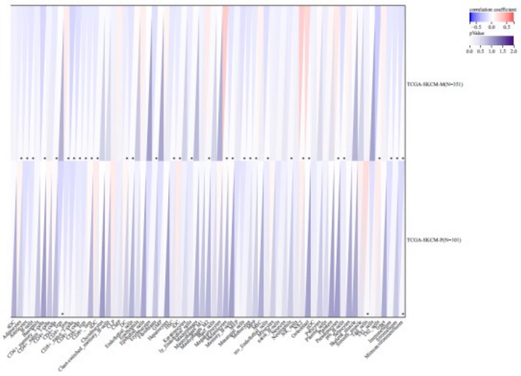

LUM

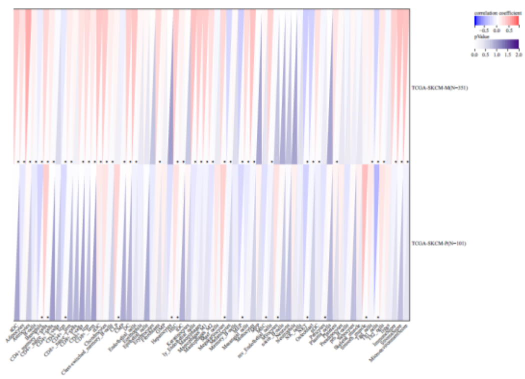

MSX1

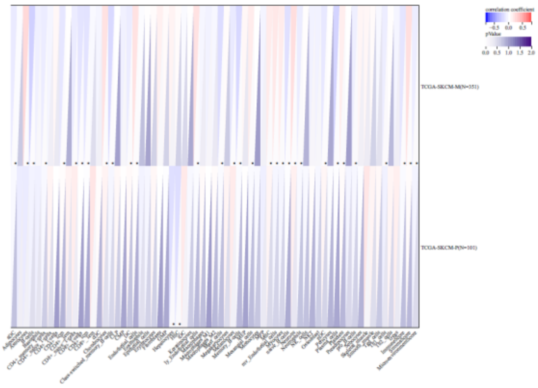

NRP1

OLR1

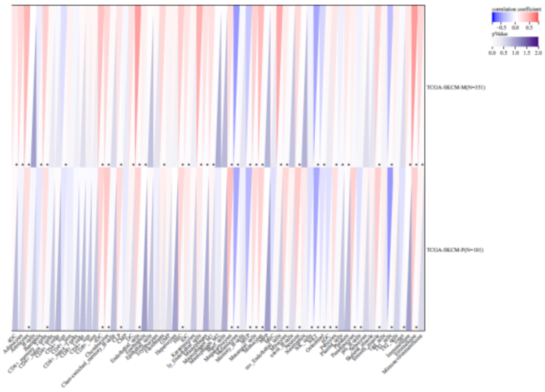

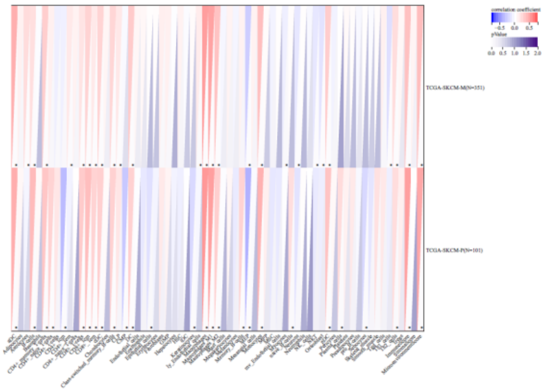

PDGFA

PF4

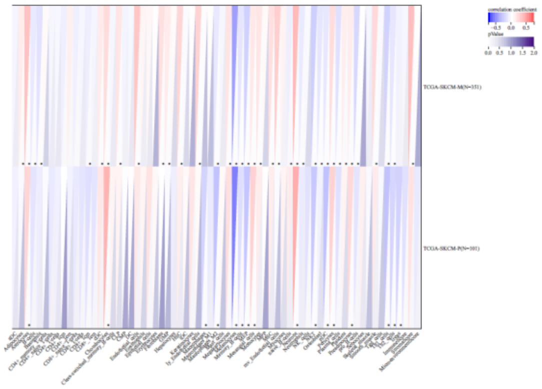

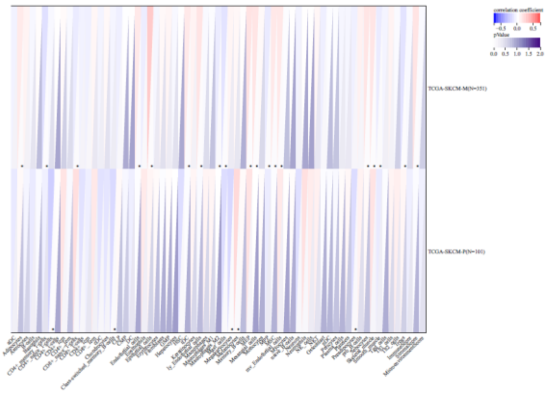

PGLYRP1

POSTN

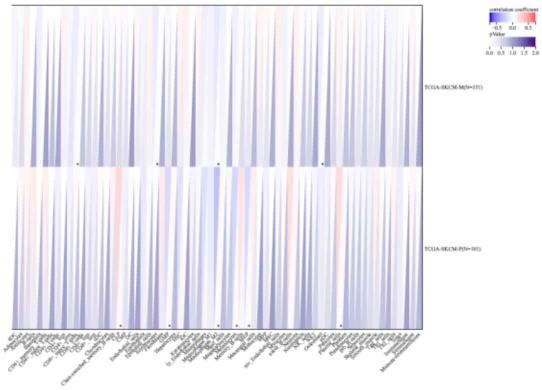

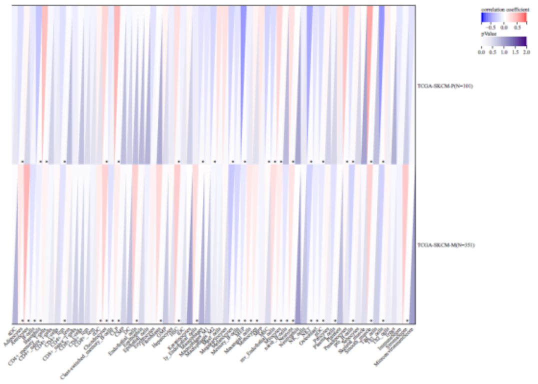

PRG2

PTK2

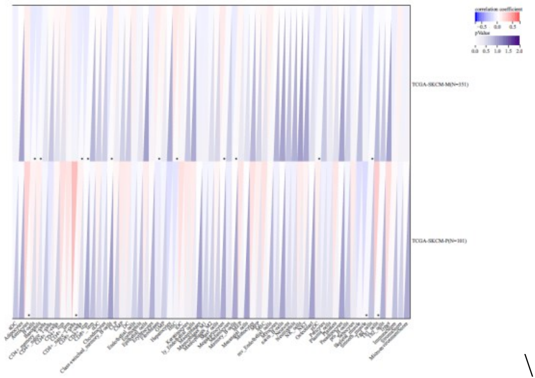

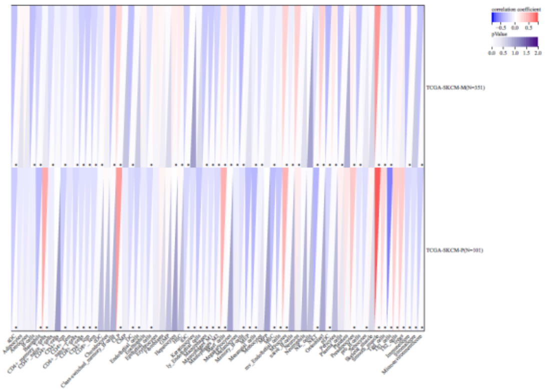

S100A4

SERPINA5

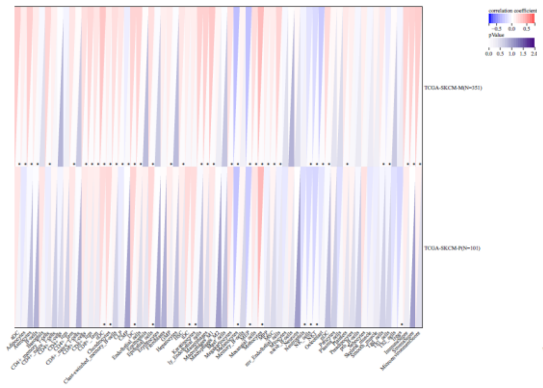

SLCO2A1

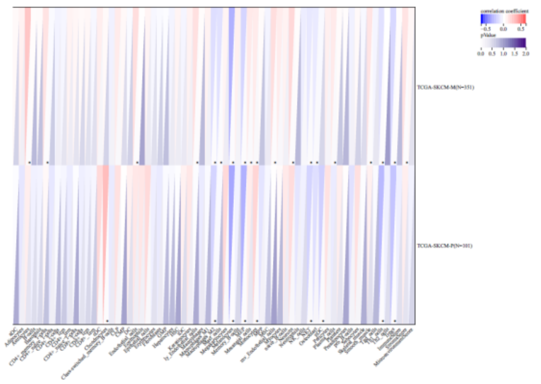

SPP1

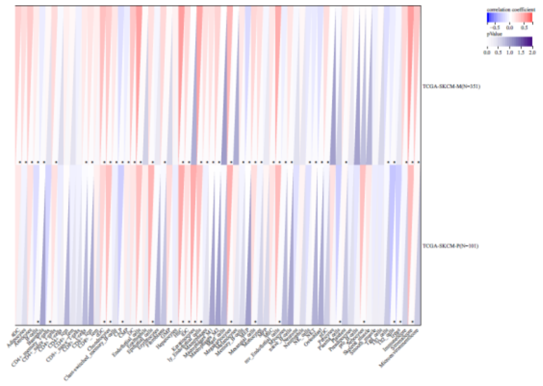

STC1

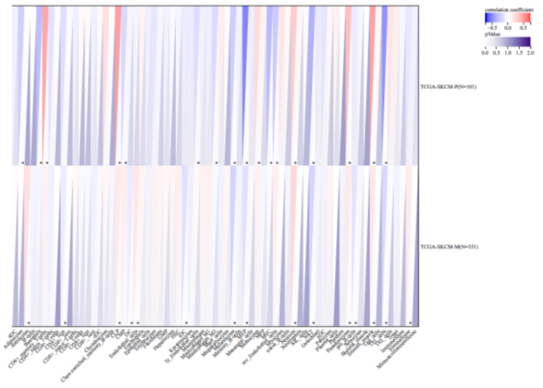

THBD

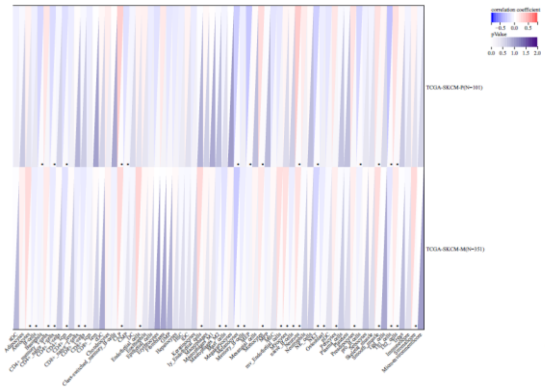

TIMP1

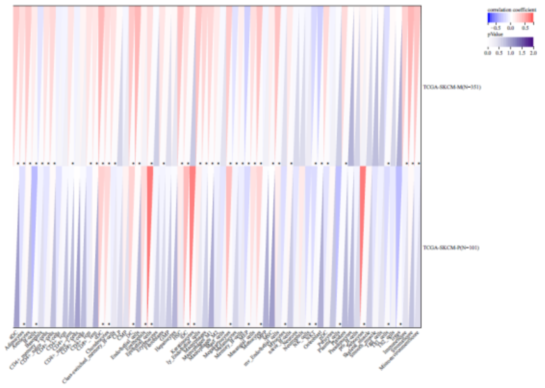

TNFRSF21

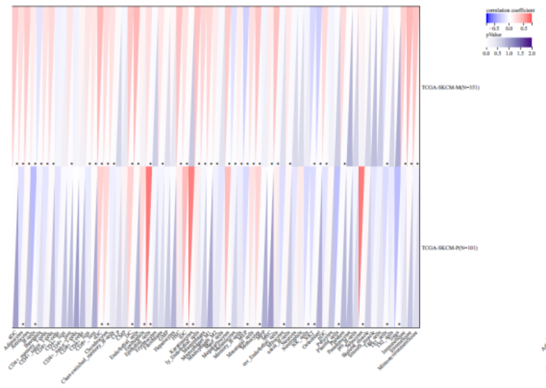

VAV2

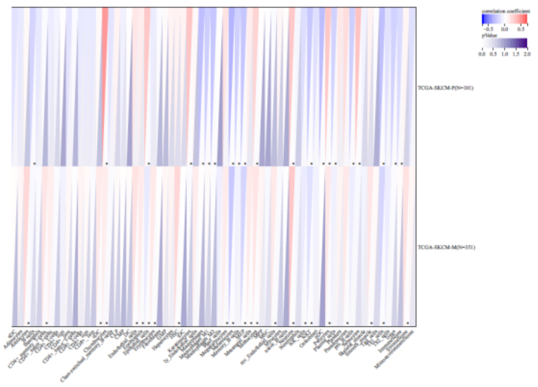

VCAN

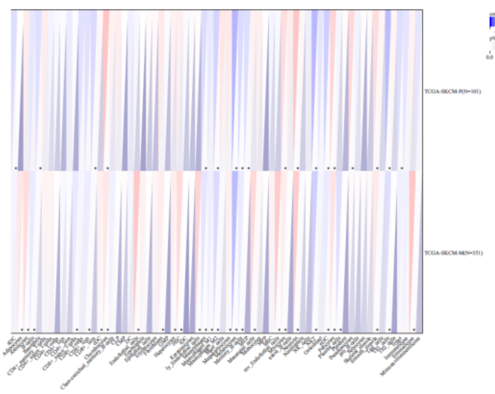

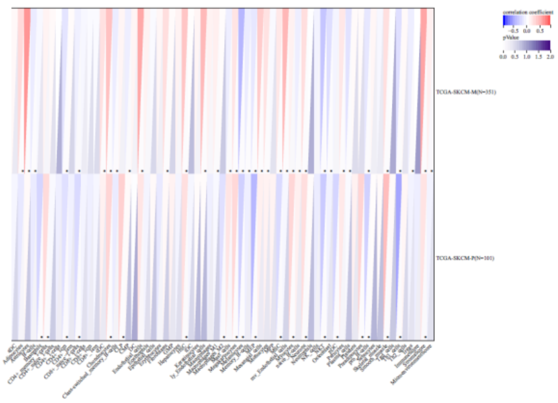

VEGFA

VTN

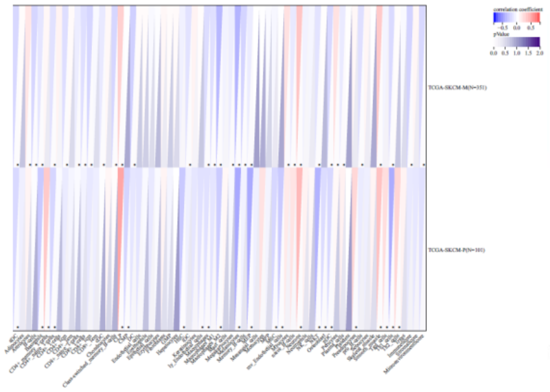

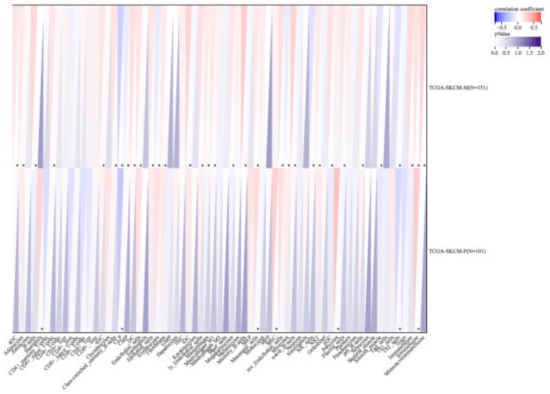

Figure S3. Spearman's correlations between the expression of ARGs and the infiltration in different immune cells in primary and metastatic skin melanoma, using xCELL. Asterisks denote statistical significance: *, p<0.05; ****, p<0.001.

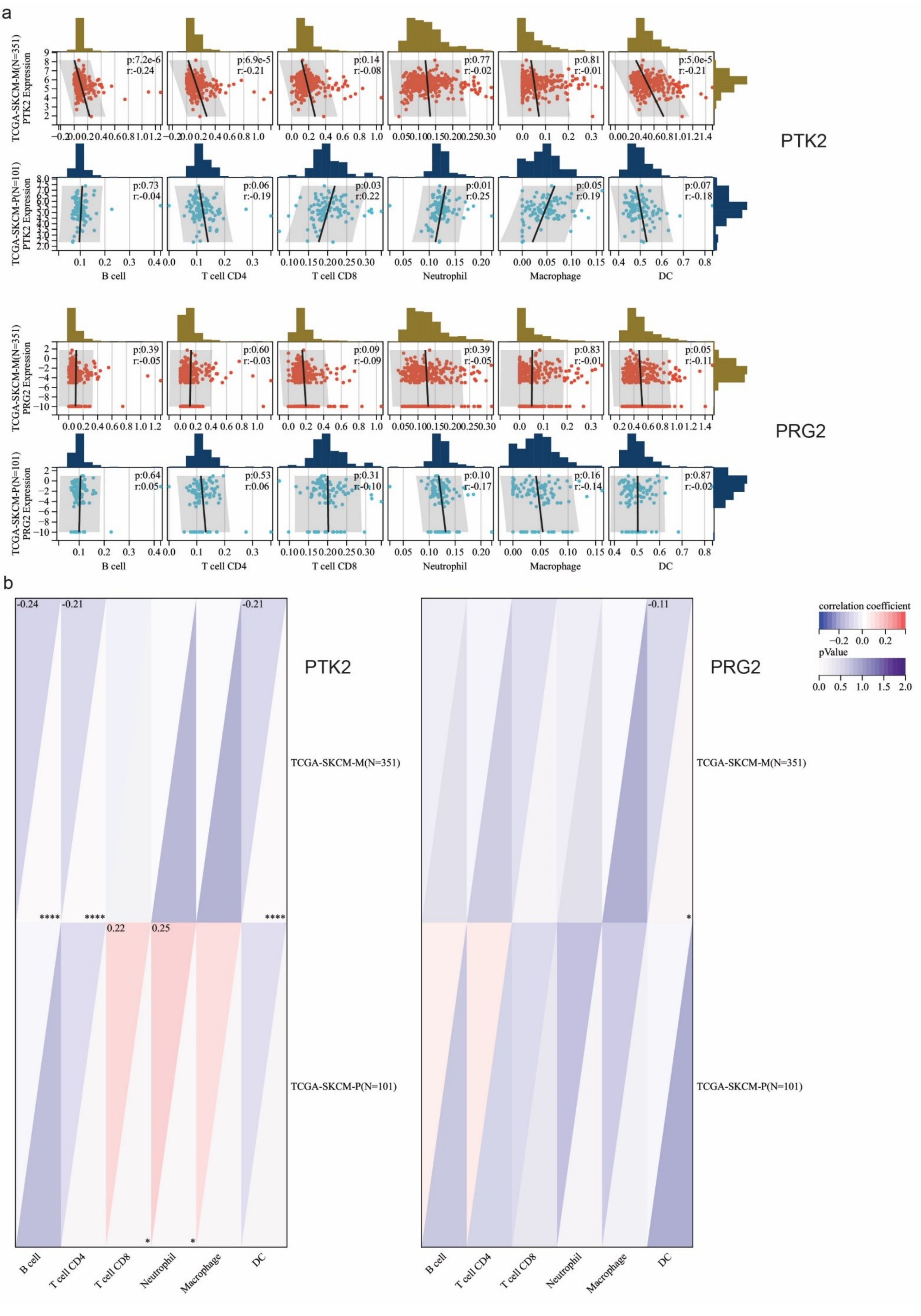

a
PTK2
PRG2
TCGA-SKCM-M(N=351) PTK2 Expression
TCGA-SKCM-P(N=101) PTK2 Expression
TCGA-SKCM-M(N=351) PRG2 Expression
TCGA-SKCM-P(N=101) PRG2 Expression
p:7.2e-6 r:-0.24
p:6.9e-5 r:-0.21
p:0.14 r:-0.08
p:0.77 r:-0.02
p:0.81 r:-0.01
p:5.0e-5 r:-0.21
p:0.73 r:-0.04
p:0.06 r:-0.19
p:0.03 r:0.22
p:0.01 r:0.25
p:0.05 r:0.19
p:0.07 r:-0.18
p:0.39 r:-0.05
p:0.60 r:-0.03
p:0.09 r:-0.09
p:0.39 r:-0.05
p:0.83 r:-0.01
p:0.05 r:-0.11
p:0.64 r:0.05
p:0.53 r:0.06
p:0.31 r:-0.10
p:0.10 r:-0.17
p:0.16 r:-0.14
p:0.87 r:-0.02
B cell
T cell CD4
T cell CD8
Neutrophil
Macrophage
DC
b
PTK2
PRG2
-0.24
-0.21
-0.21
****
****
****
0.22
0.25
*
*
-0.11
*
TCGA-SKCM-M(N=351)
TCGA-SKCM-P(N=101)
correlation coefficient
−0.2 0.0 0.2
pValue
0.0 0.5 1.0 1.5 2.0

Figure S4. Spearman's correlation analysis depicted in the form of scatterplots (**a**) and heatmaps (**b**) between the expression of *PTK2* (or *PRG2*) and immune cell populations in primary (TCGA-SCKM-P) and metastatic skin melanoma (TCGA-SCKM-M), respectively, using TIMER. Asterisks denote statistical significance: *, $p<0.05$; ****, $p<0.001$.

APOH

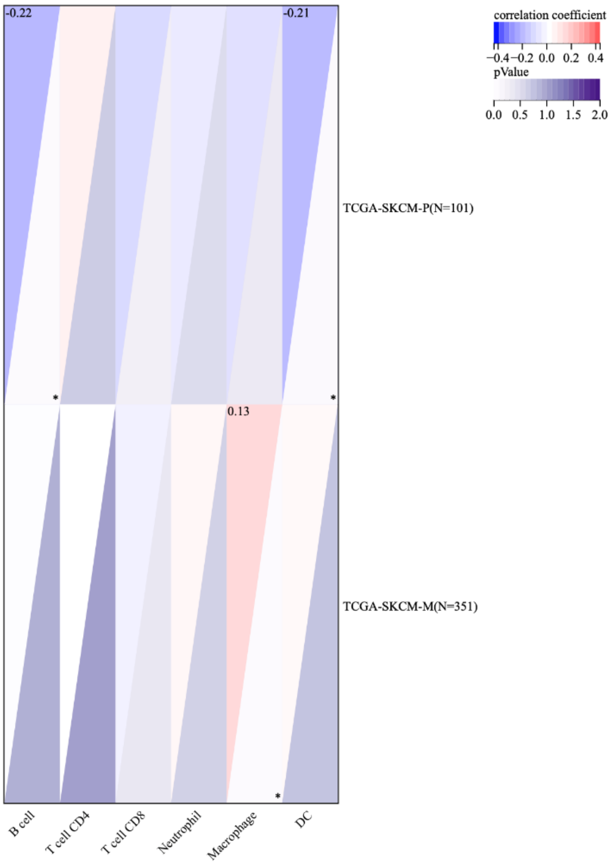

APP

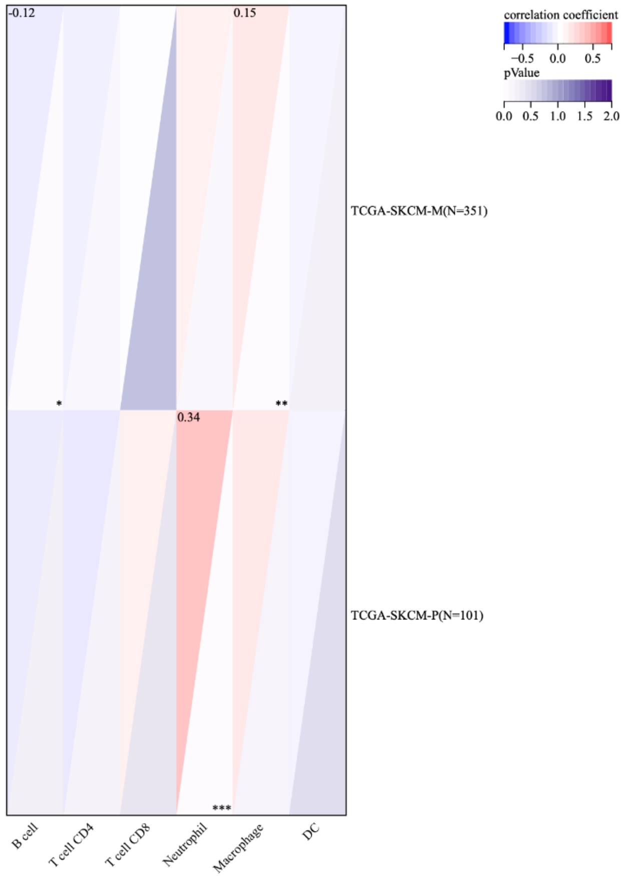

CCND2

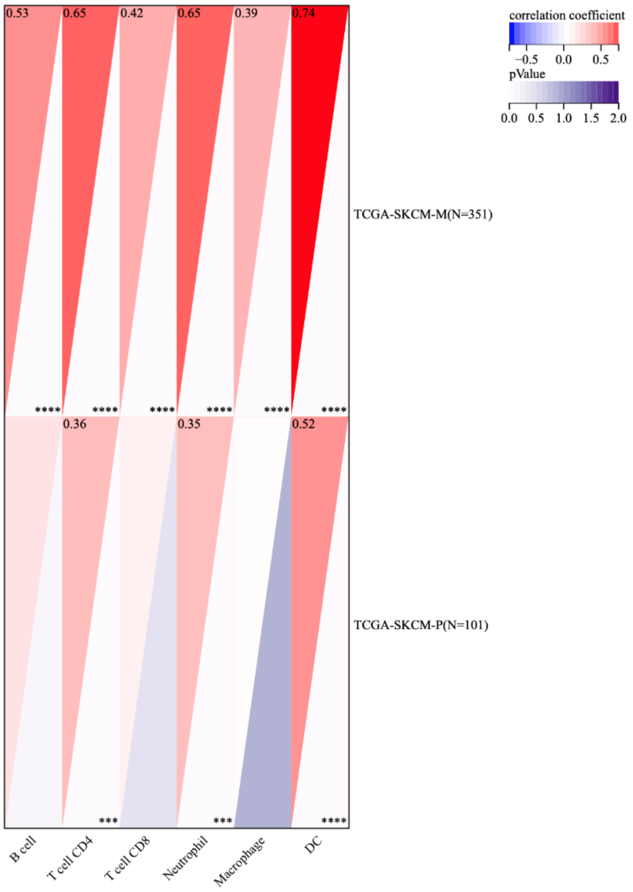

COL3A1

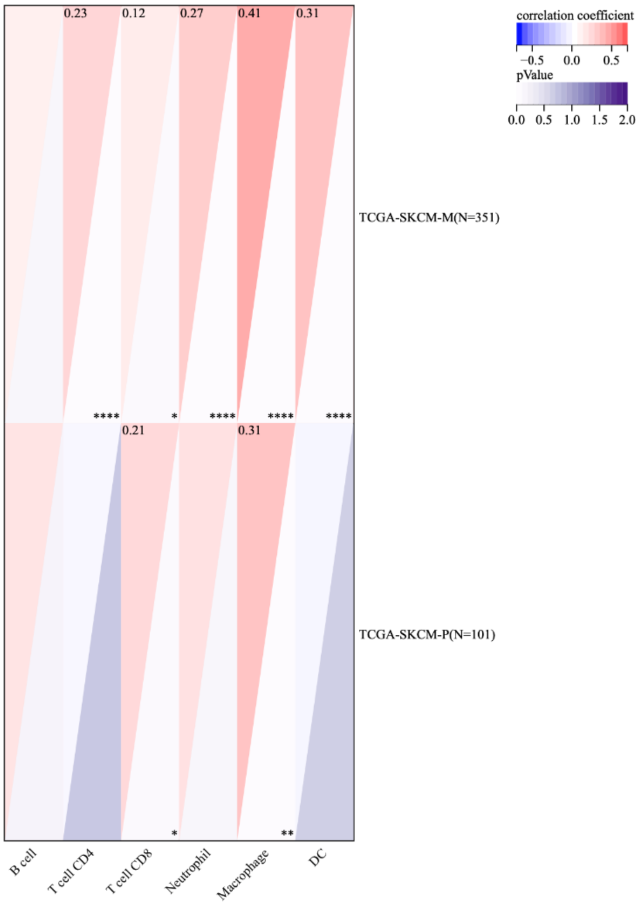

COL5A2

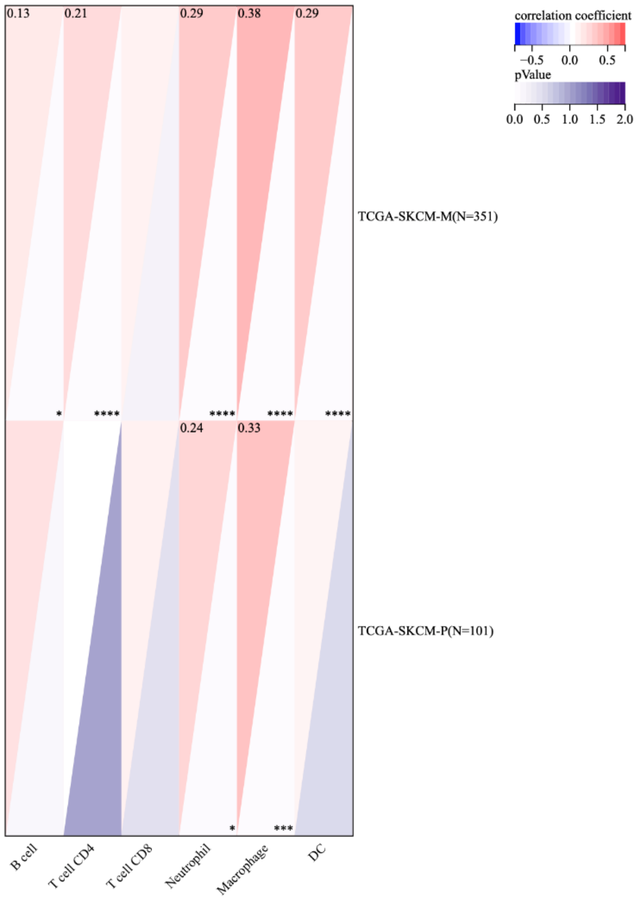

CXCL6

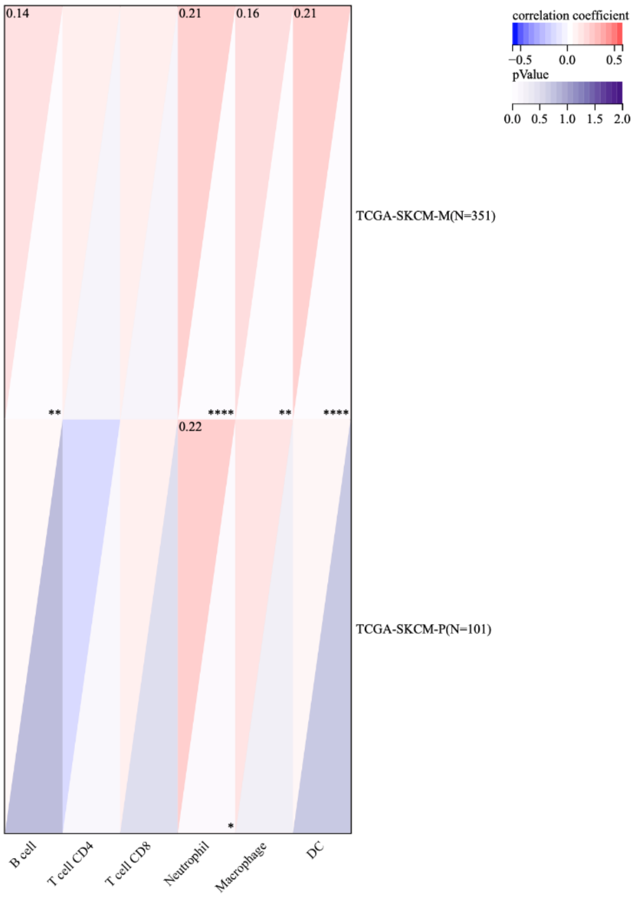

FGFR1

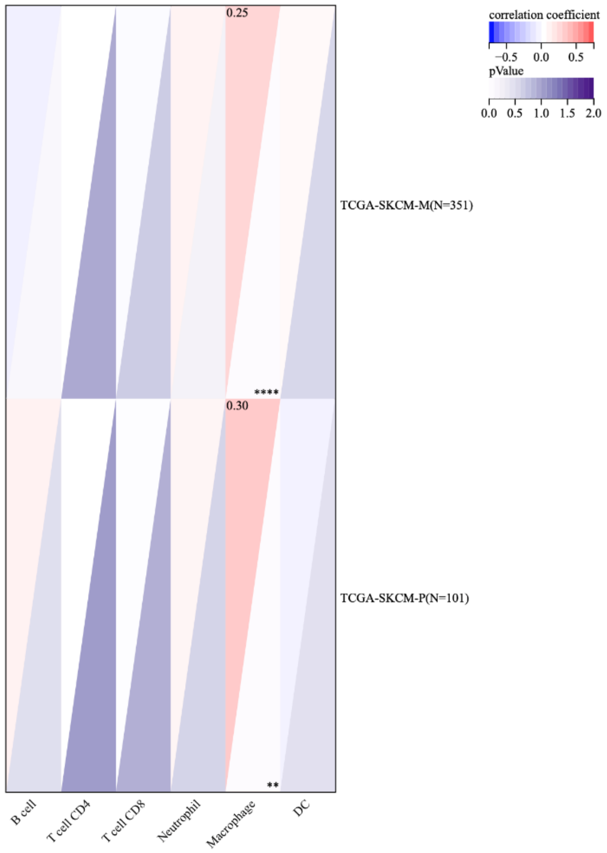

FSTL1

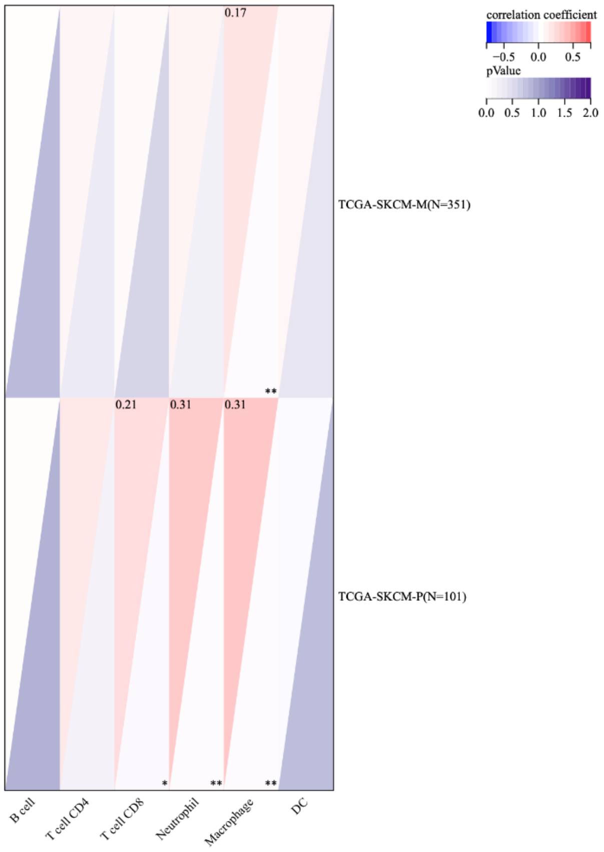

ITGAV

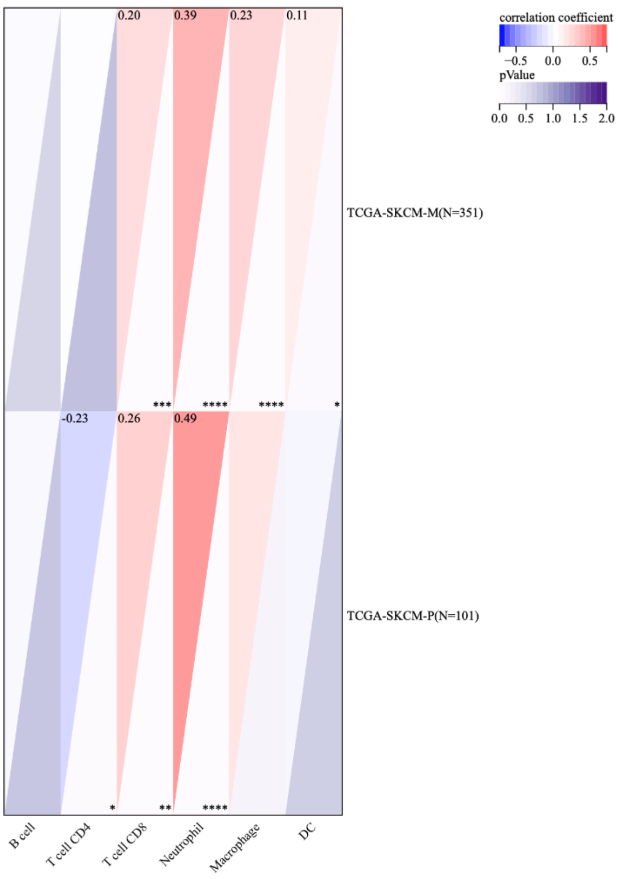

JAG1

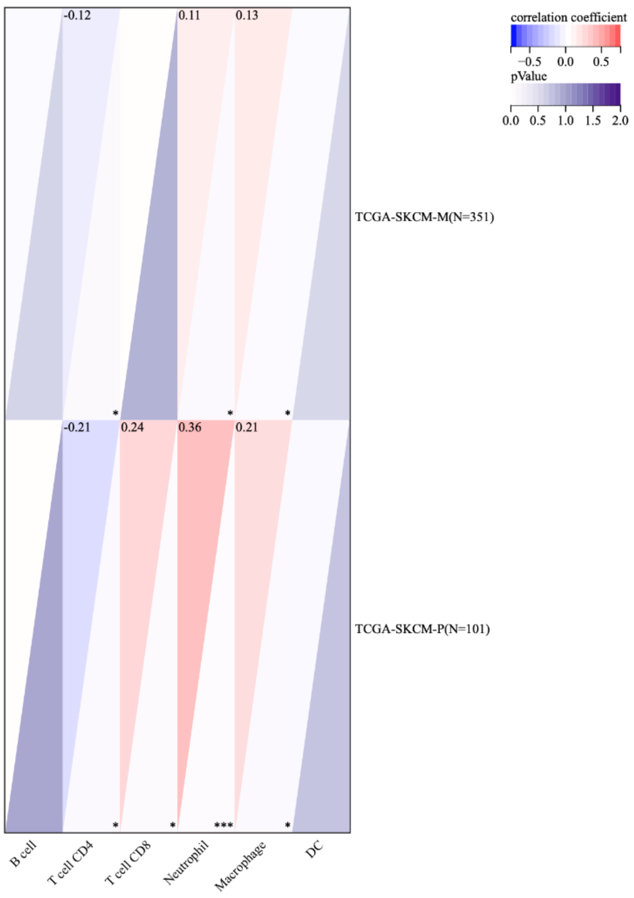

JAG2

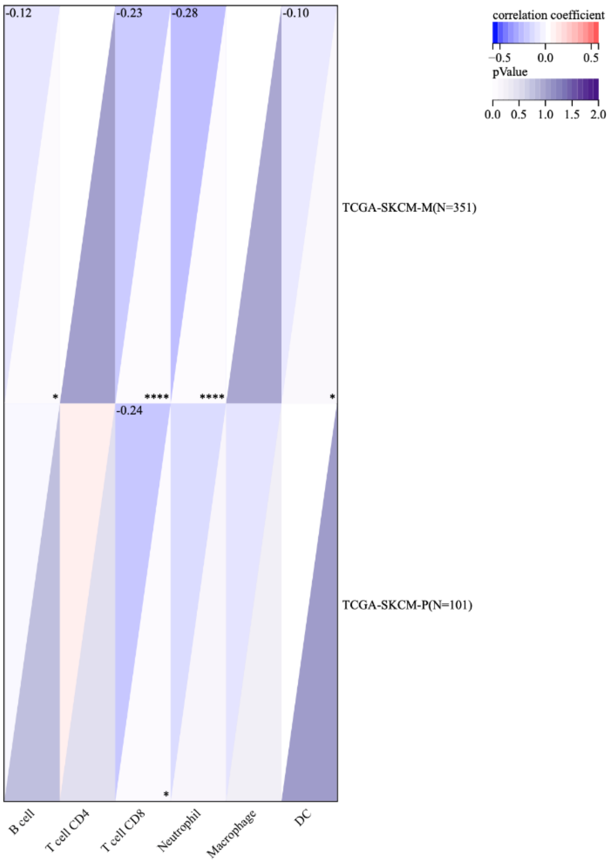

KCNJ8

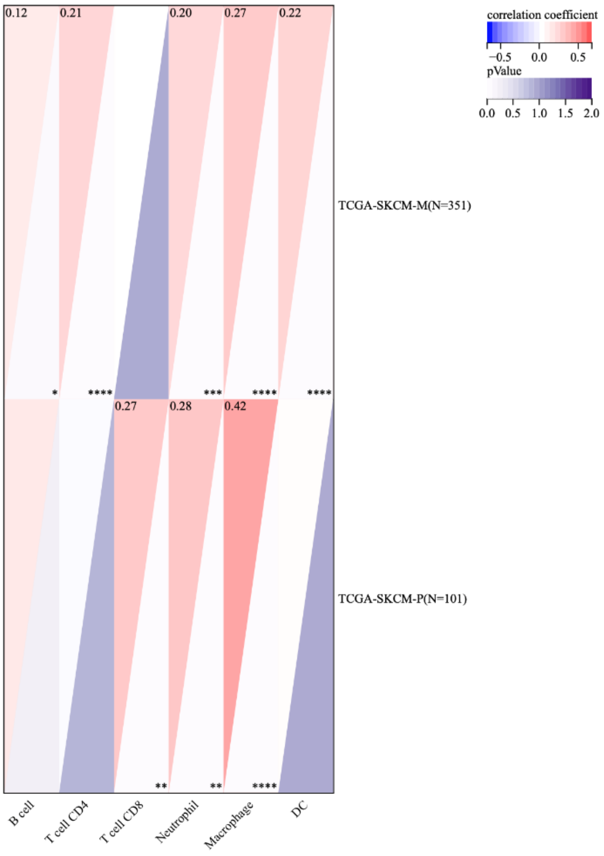

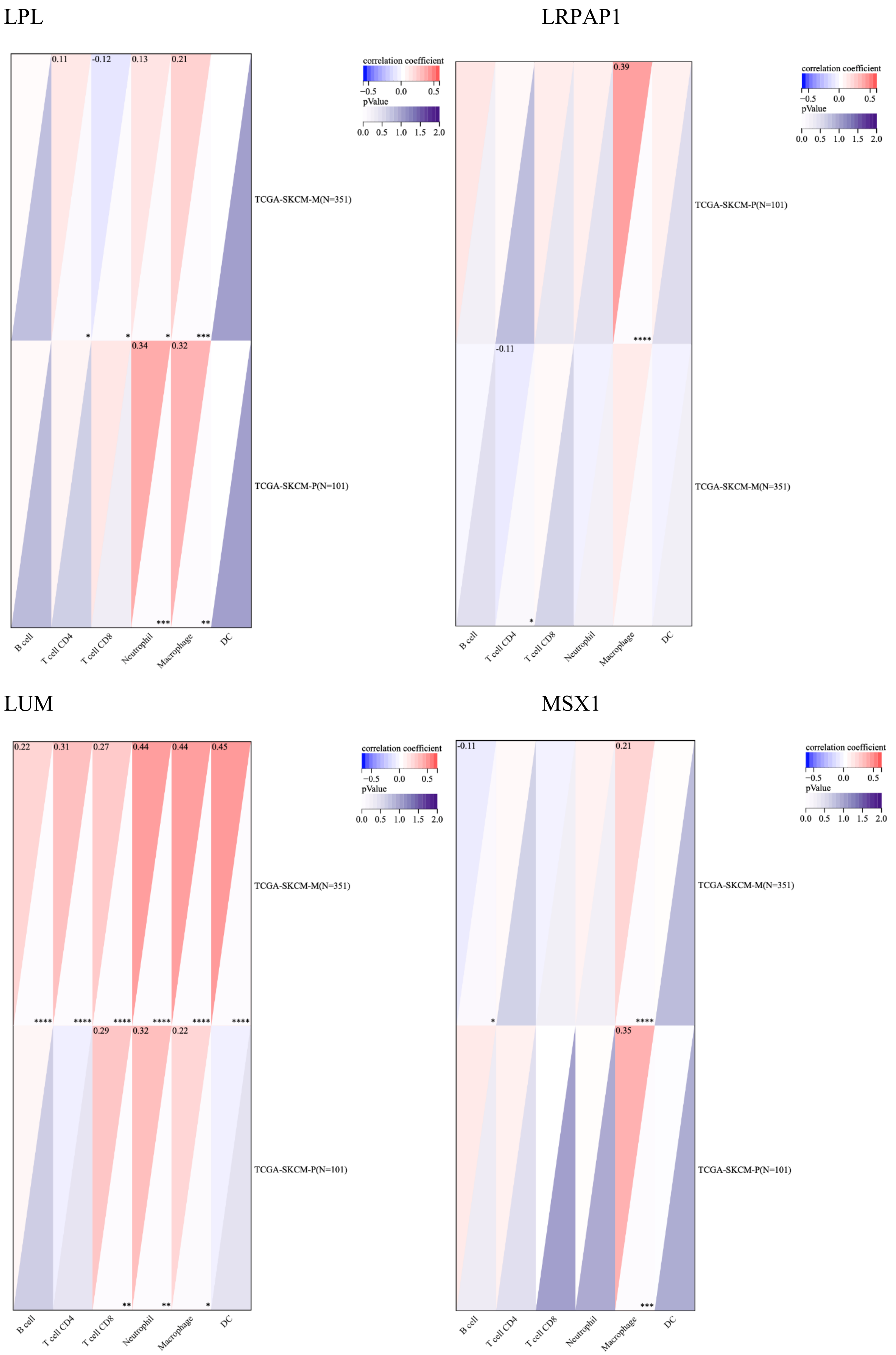
LPL
correlation coefficient
−0.5 0.0 0.5
pValue
0.0 0.5 1.0 1.5 2.0
0.11
-0.12
0.13
0.21
* * * ***
TCGA-SKCM-M(N=351)
0.34
0.32
*** **
TCGA-SKCM-P(N=101)
B cell
T cell CD4
T cell CD8
Neutrophil
Macrophage
DC
LRPAP1
correlation coefficient
−0.5 0.0 0.5
pValue
0.0 0.5 1.0 1.5 2.0
0.39
****
TCGA-SKCM-P(N=101)
-0.11
*
TCGA-SKCM-M(N=351)
B cell
T cell CD4
T cell CD8
Neutrophil
Macrophage
DC
LUM
correlation coefficient
−0.5 0.0 0.5
pValue
0.0 0.5 1.0 1.5 2.0
0.22
0.31
0.27
0.44
0.44
0.45
**** **** **** **** **** ****
TCGA-SKCM-M(N=351)
0.29
0.32
0.22
** ** *
TCGA-SKCM-P(N=101)
B cell
T cell CD4
T cell CD8
Neutrophil
Macrophage
DC
MSX1
correlation coefficient
−0.5 0.0 0.5
pValue
0.0 0.5 1.0 1.5 2.0
-0.11
0.21
* ****
TCGA-SKCM-M(N=351)
0.35
***
TCGA-SKCM-P(N=101)
B cell
T cell CD4
T cell CD8
Neutrophil
Macrophage
DC

NRP1

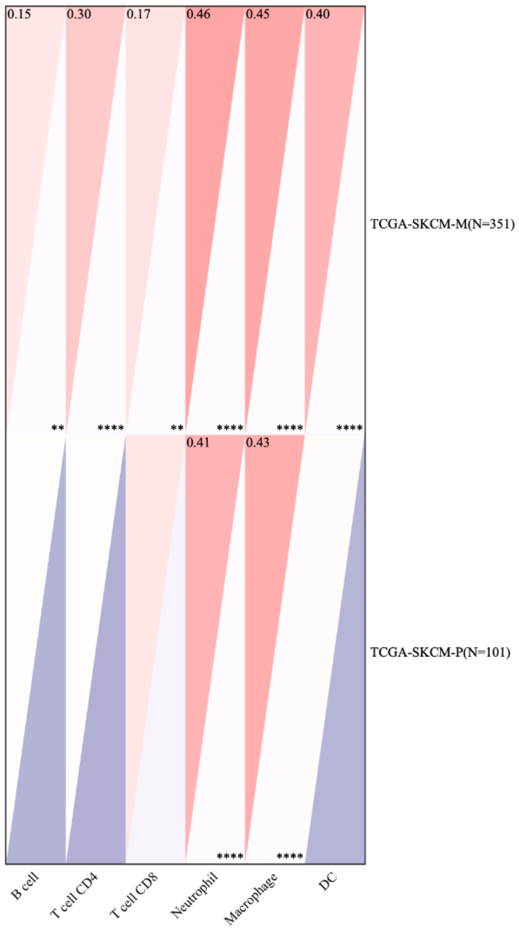

OLR1

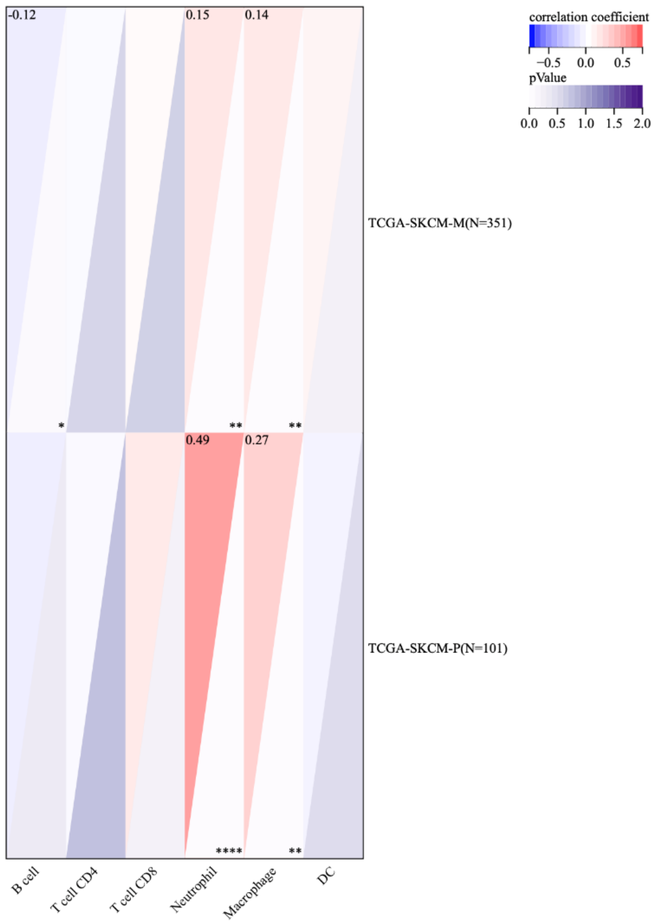

PDGFA

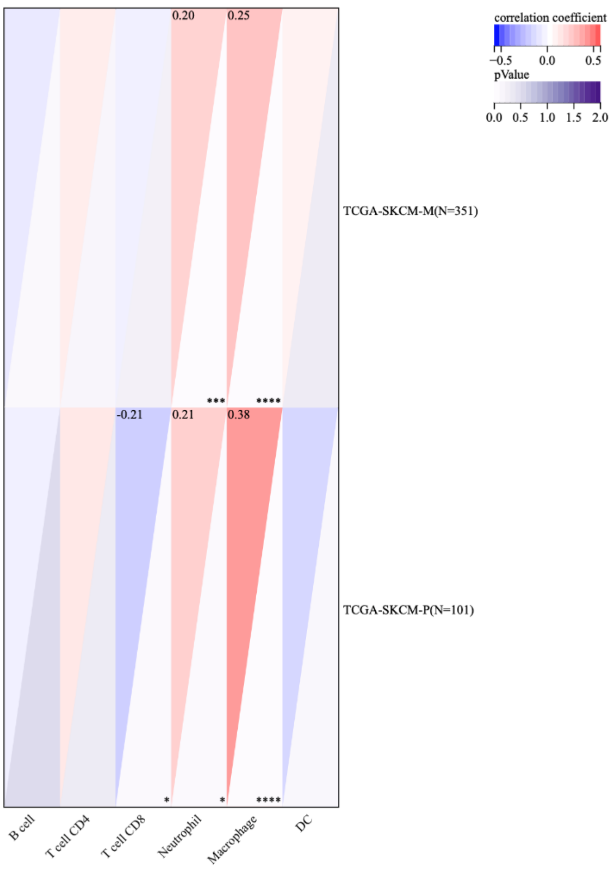

PF4

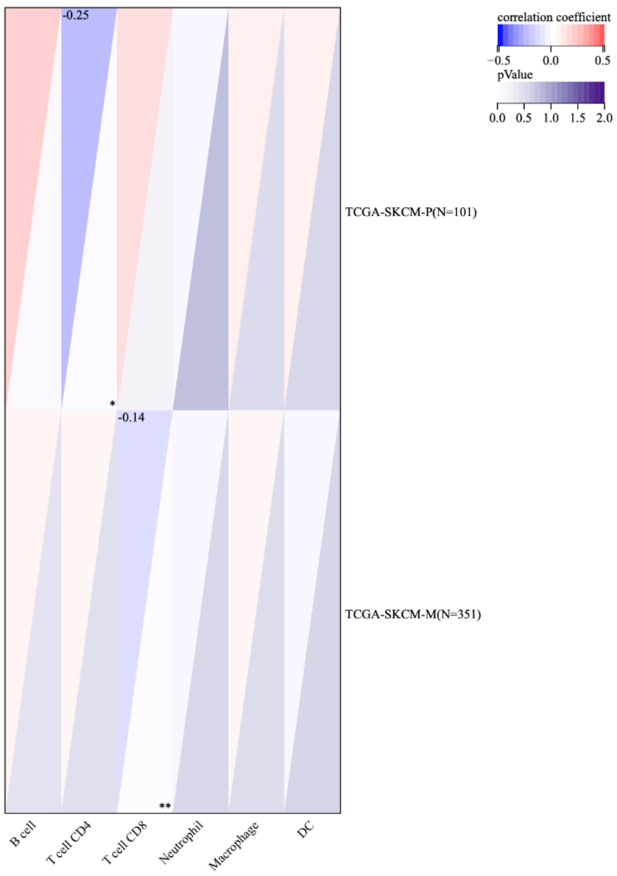

PGLYRP1

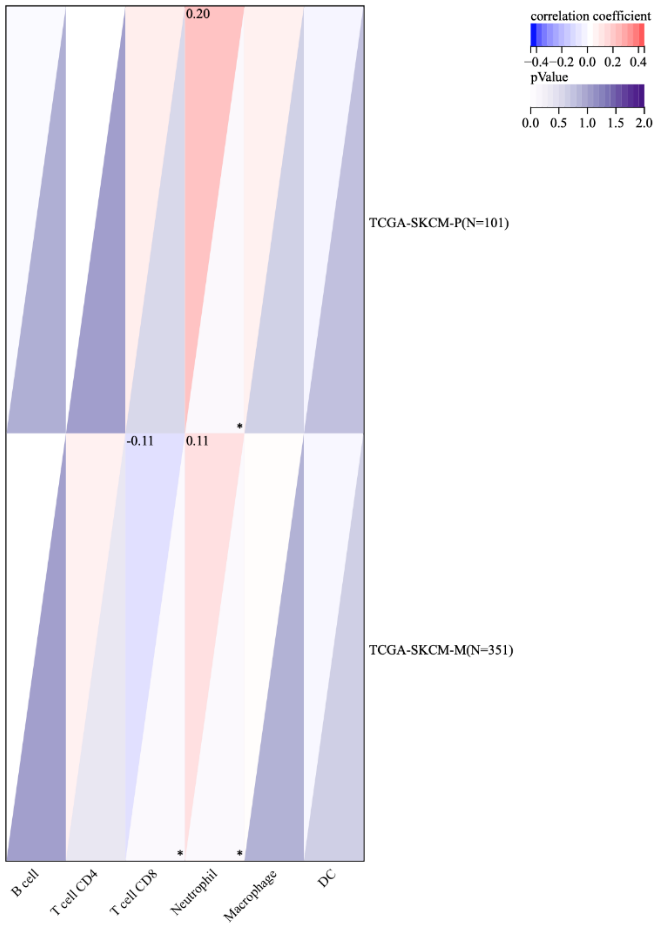

POSTN

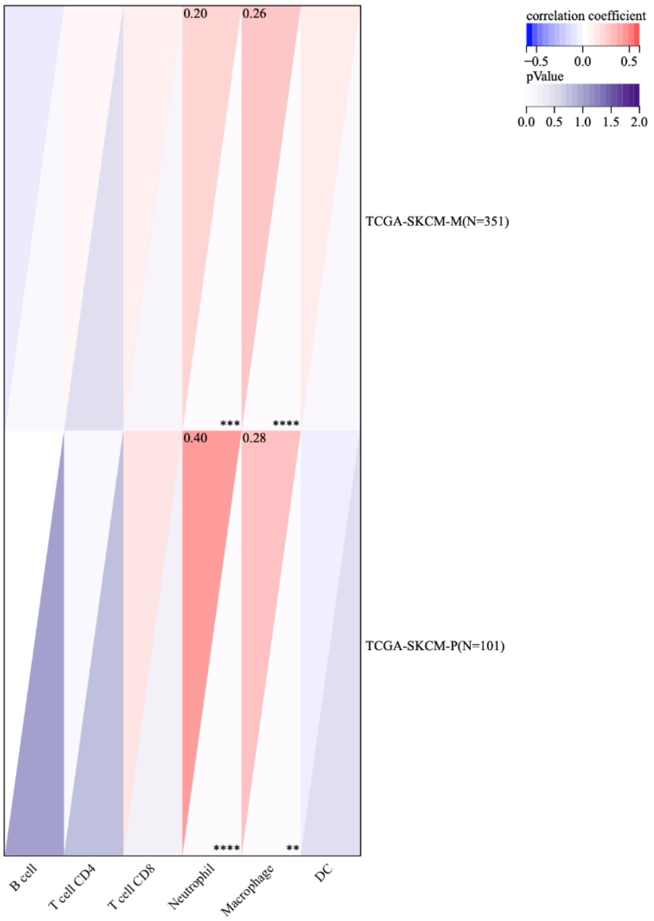

PRG2

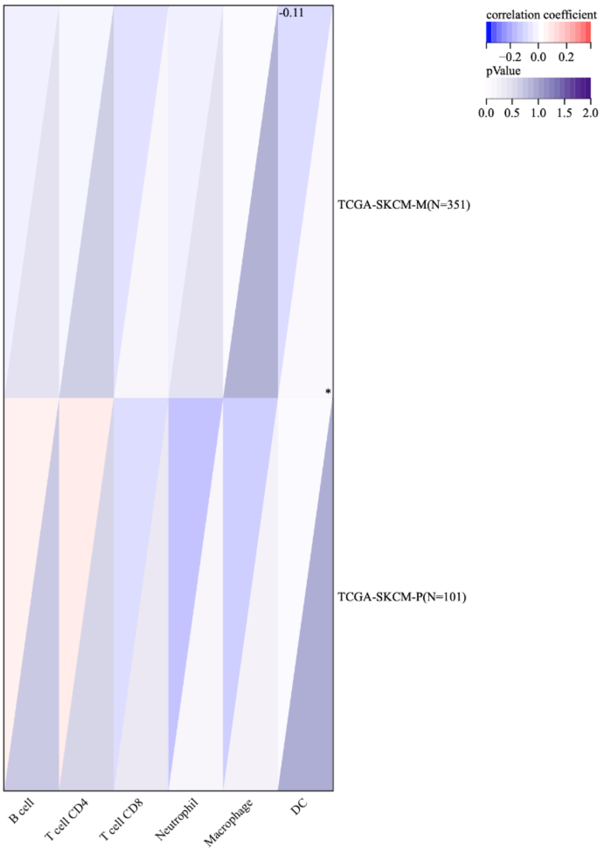

PTK2

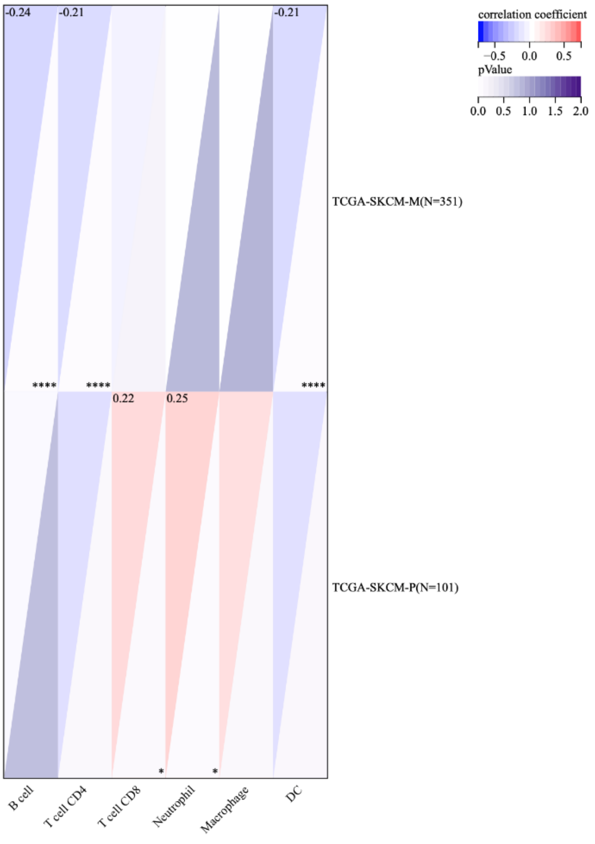

S100A4

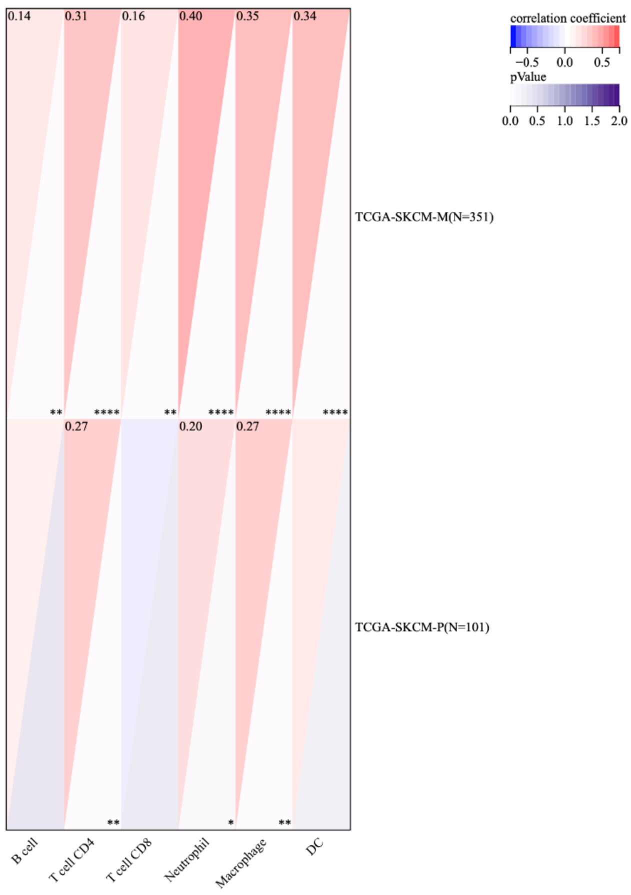

SERPINA5

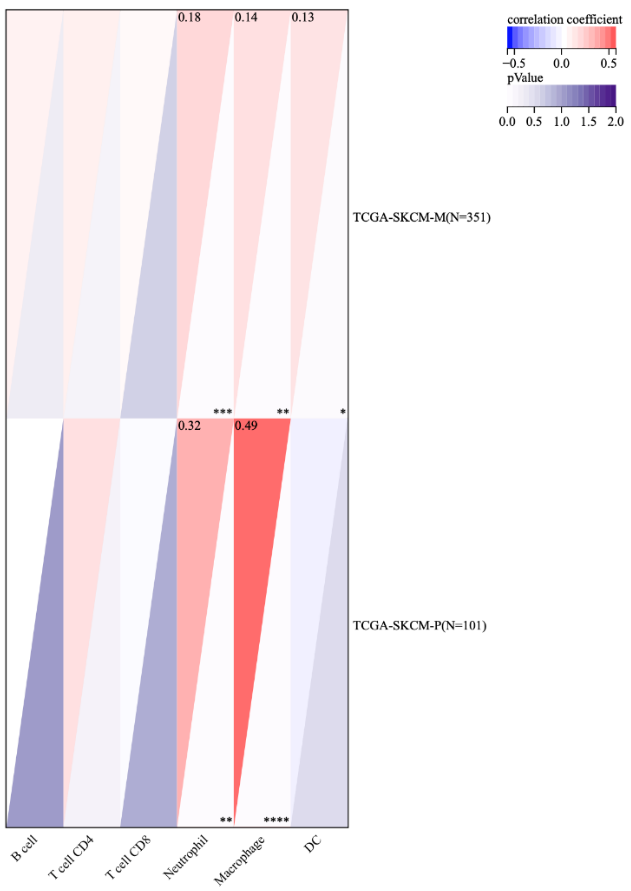

SLCO2A1

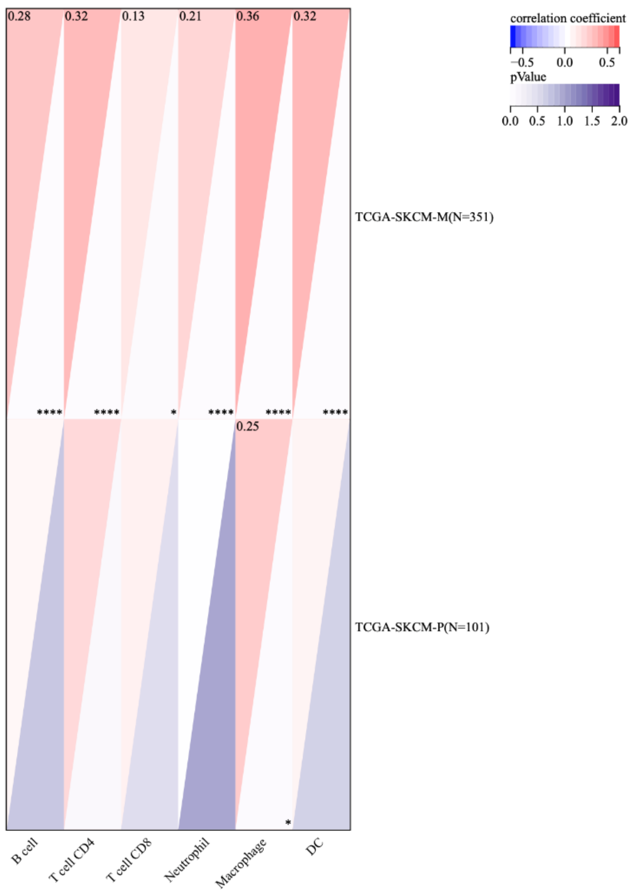

SPP1

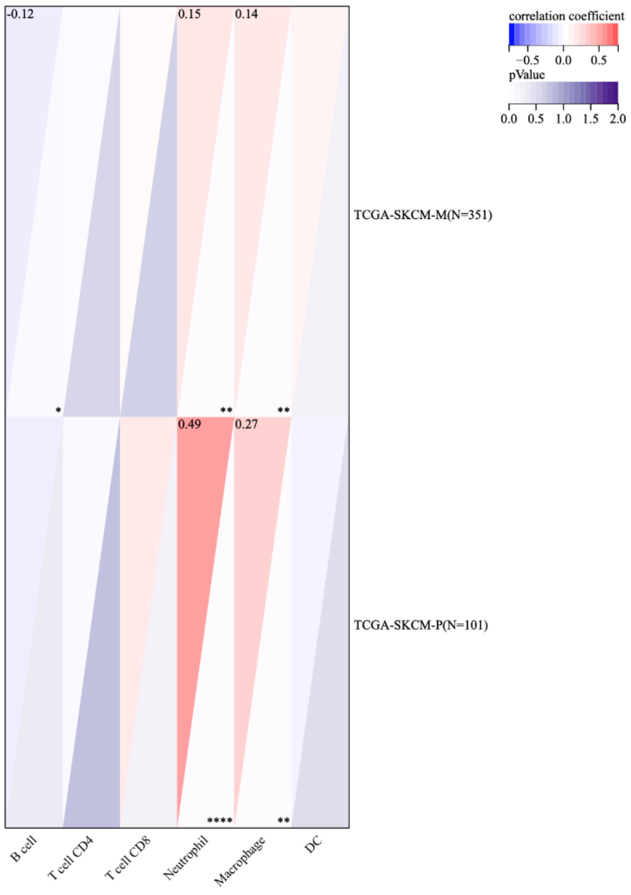

STC1

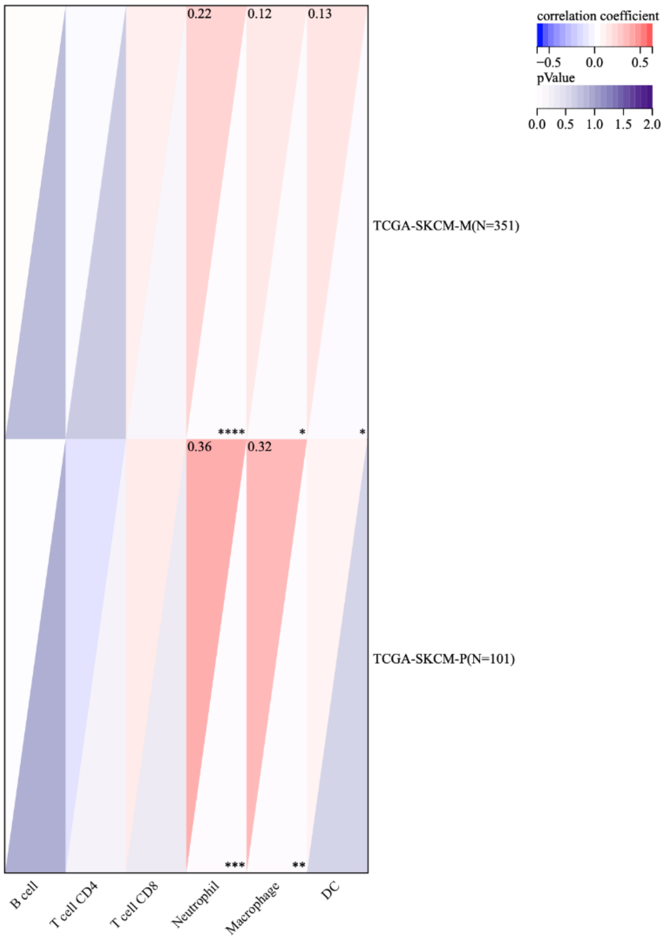

THBD

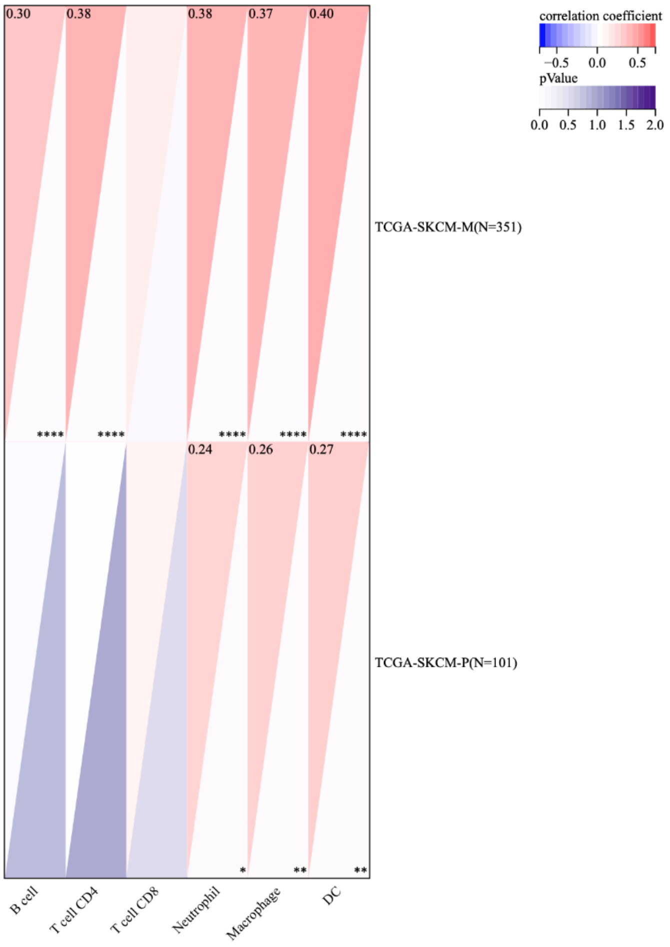

TIMP1

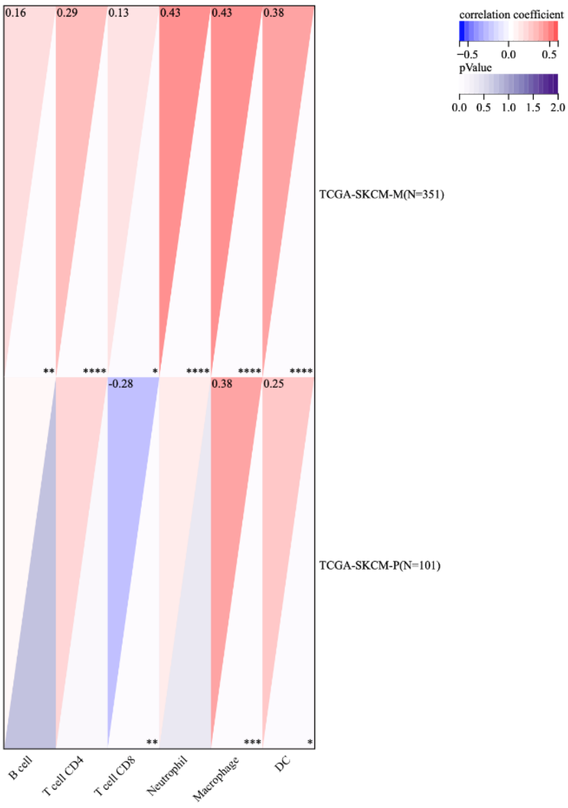

TNFRSF21

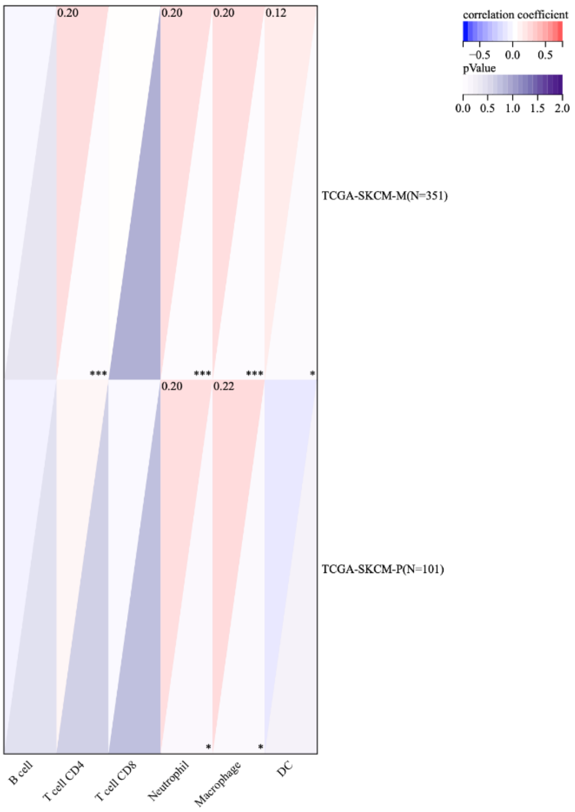

VAV2

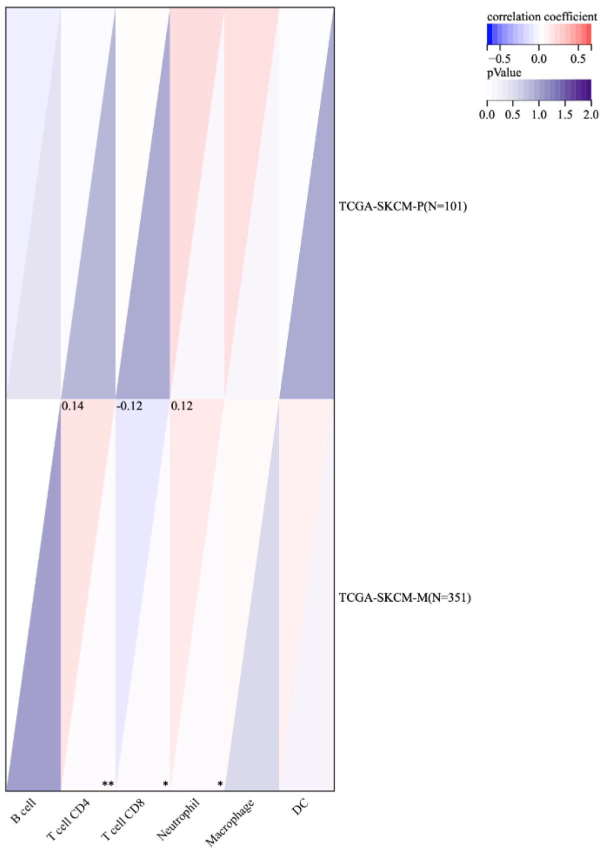

VCAN

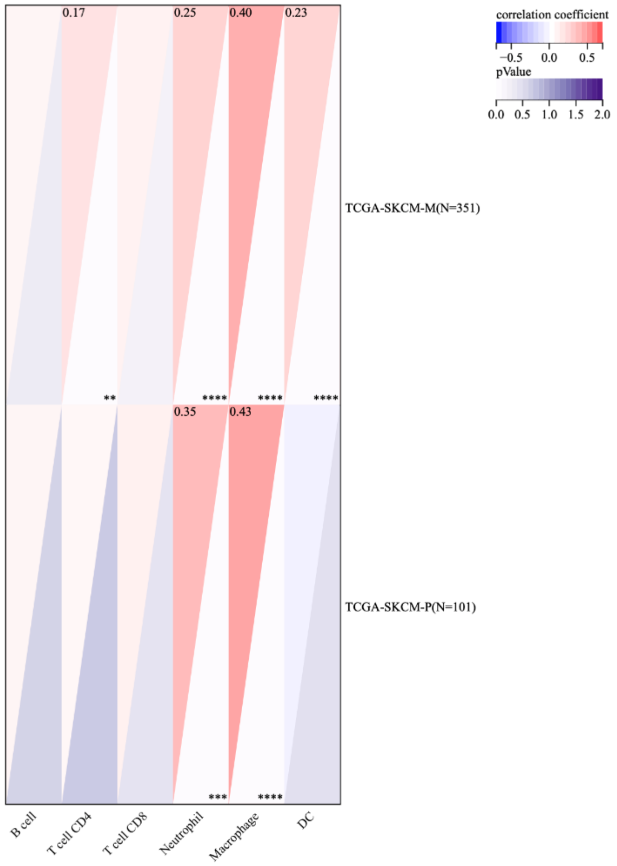

VEGFA

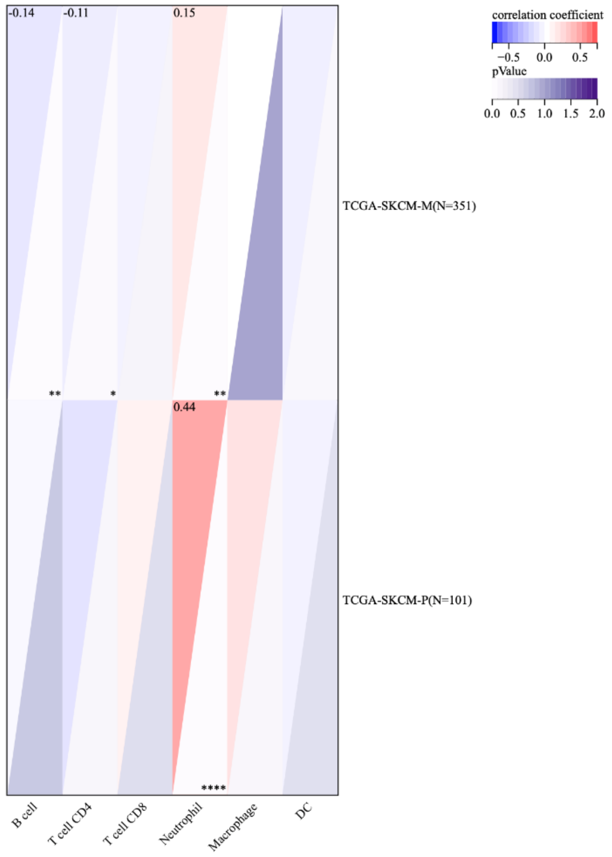

Figure S5. Spearman's correlation analysis depicted in the form of scatterplots (**a**) and heatmaps (**b**) between the expression of ARGs and immune cell populations in primary (TCGA-SCKM-P) and metastatic skin melanoma (TCGA-SCKM-M), respectively, using TIMER. Asterisks denote statistical significance: *, $p<0.05$; ****, $p<0.001$.

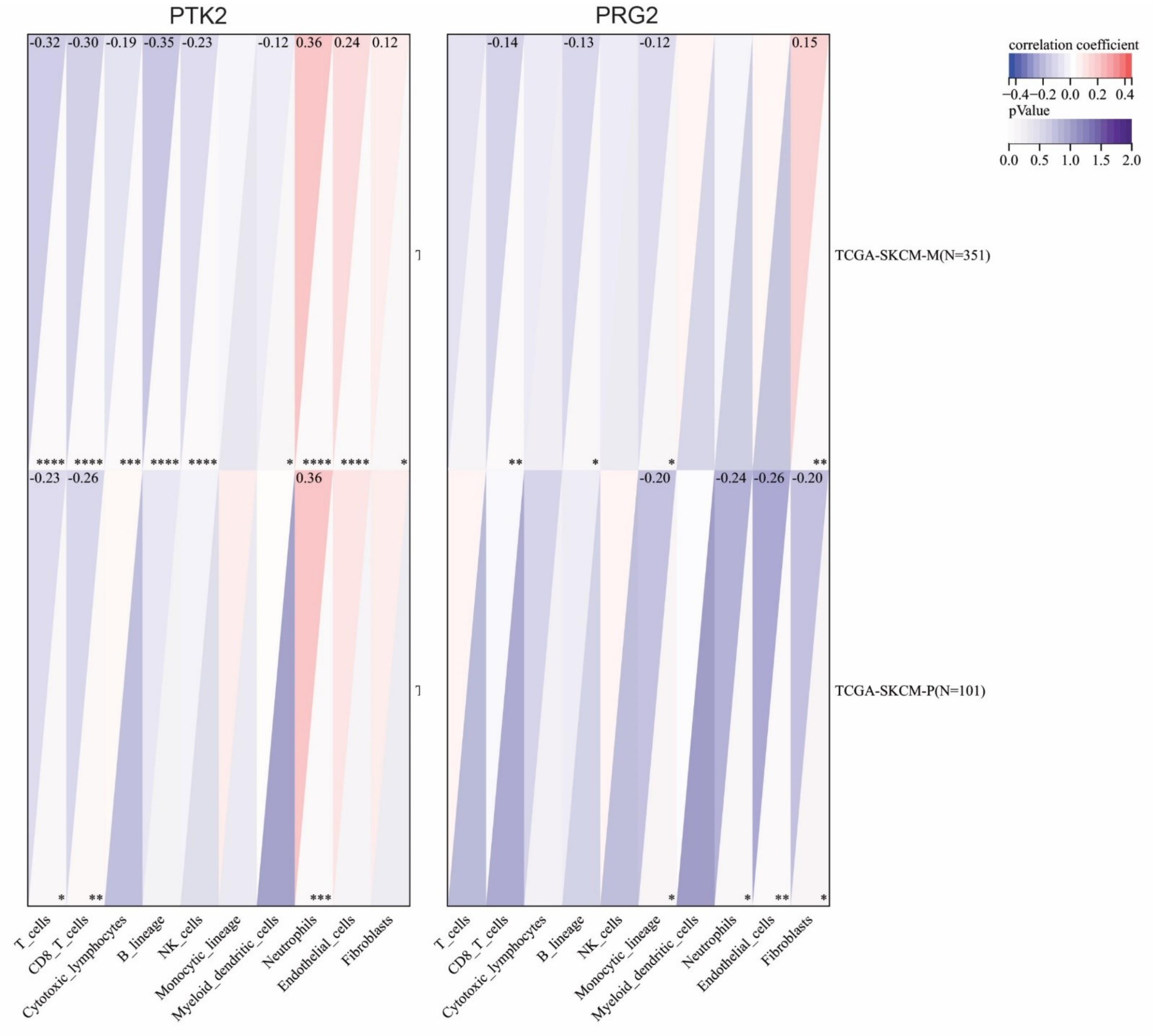

Figure S6. Spearman's correlation analysis between the expression of *PTK2* (or *PRG2*) and immune cell populations in primary (TCGA-SCKM-P) and metastatic skin melanoma (TCGA-SCKM-M), respectively, using MCPCounter. Asterisks denote statistical significance: *, $p<0.05$; **, $p<0.01$; ***, $p<0.005$; ****, $p<0.001$.

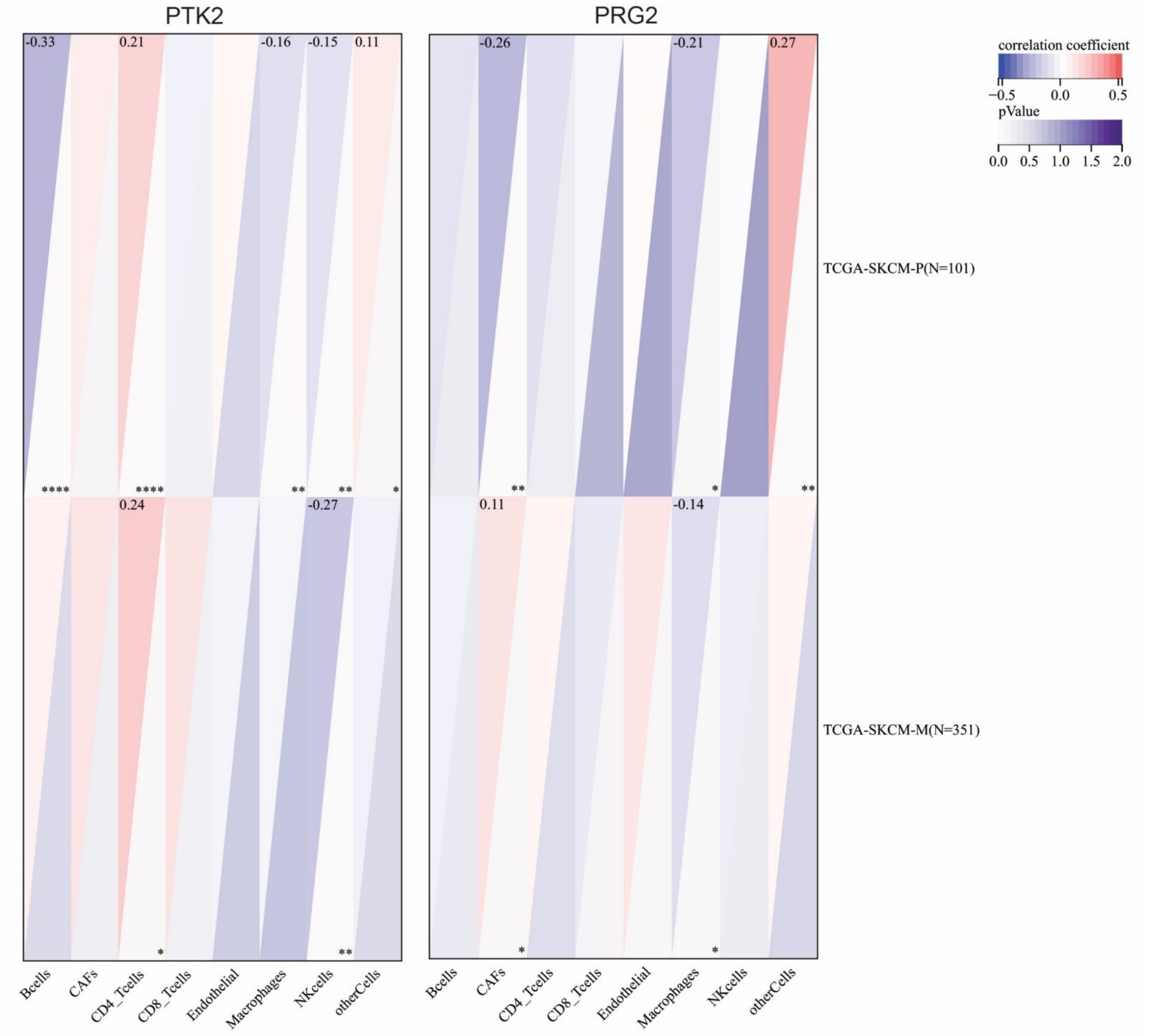

Figure S7. Spearman's correlation analysis between the expression of *PTK2* (or *PRG2*) and immune cell populations in primary (TCGA-SCKM-P) and metastatic skin melanoma (TCGA-SCKM-M), respectively, using EPIC. Asterisks denote statistical significance: *, p<0.05; ****, p<0.001.

APOH

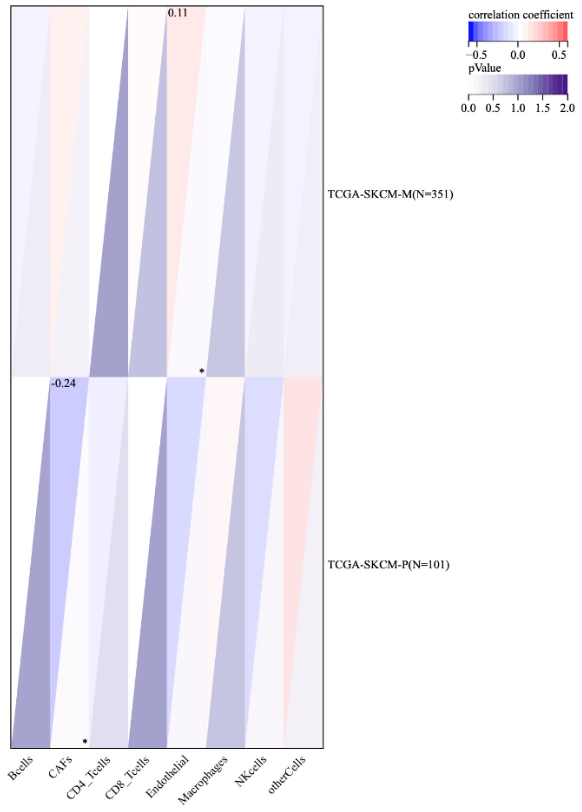

APP

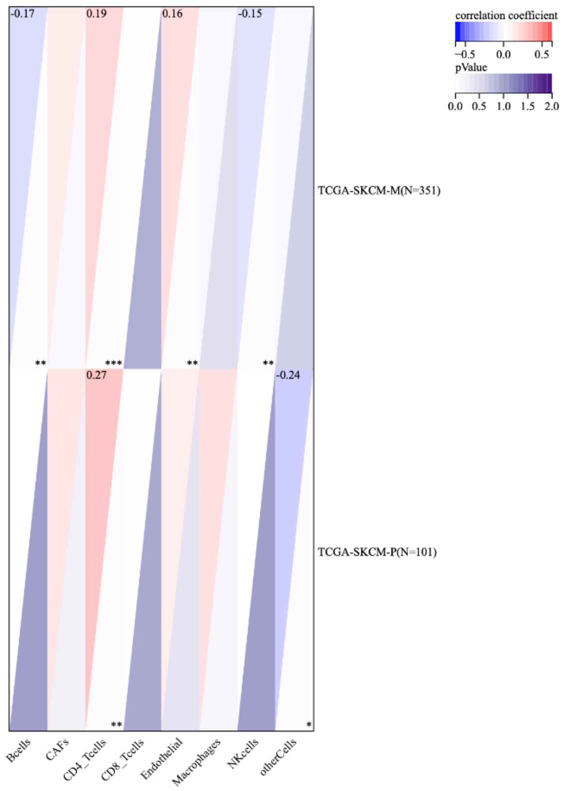

CCND2

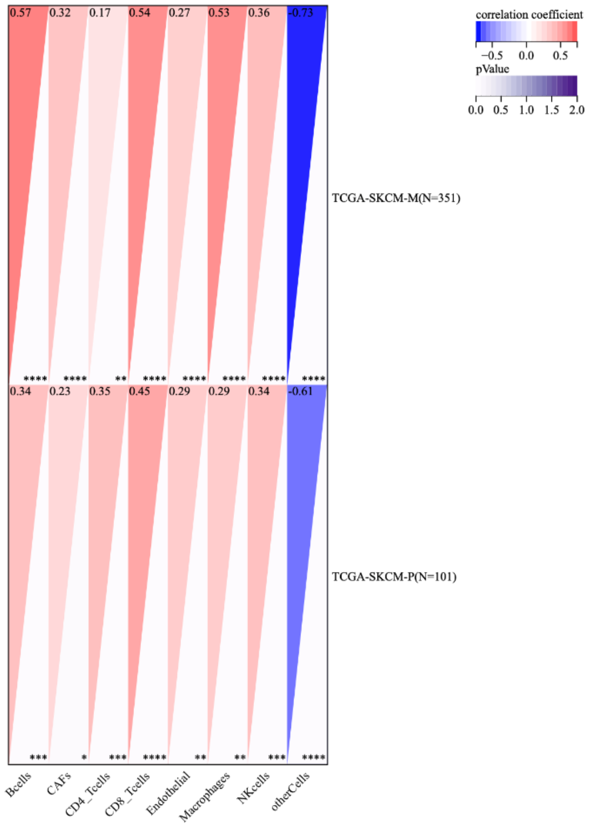

COL3A1

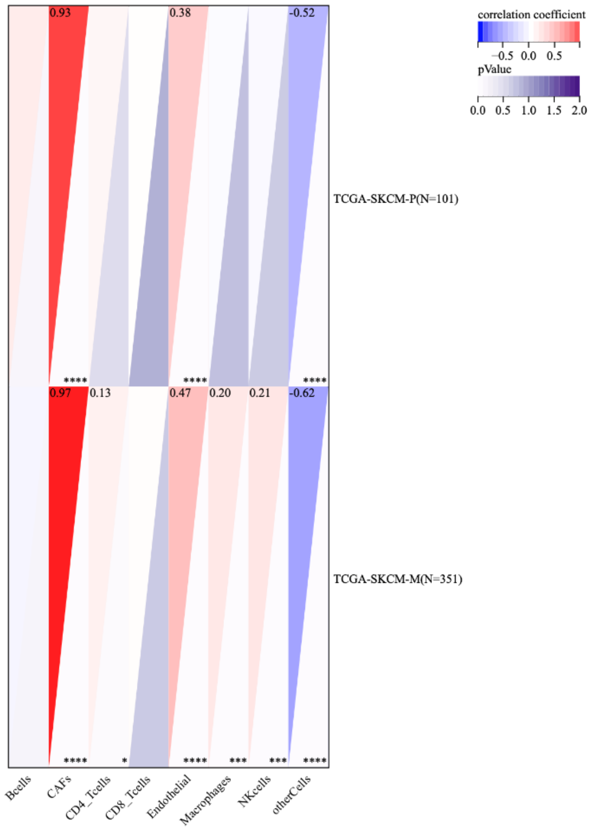

COL5A2

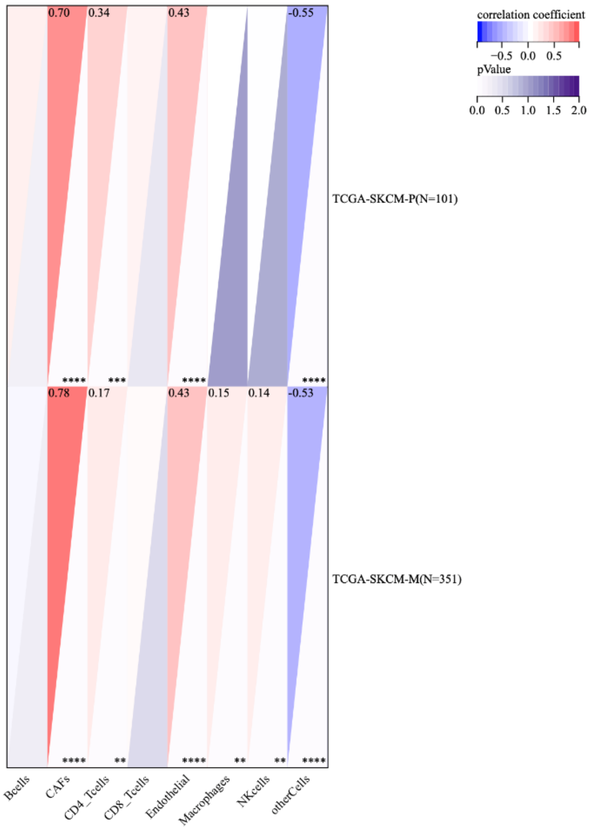

CXCL6

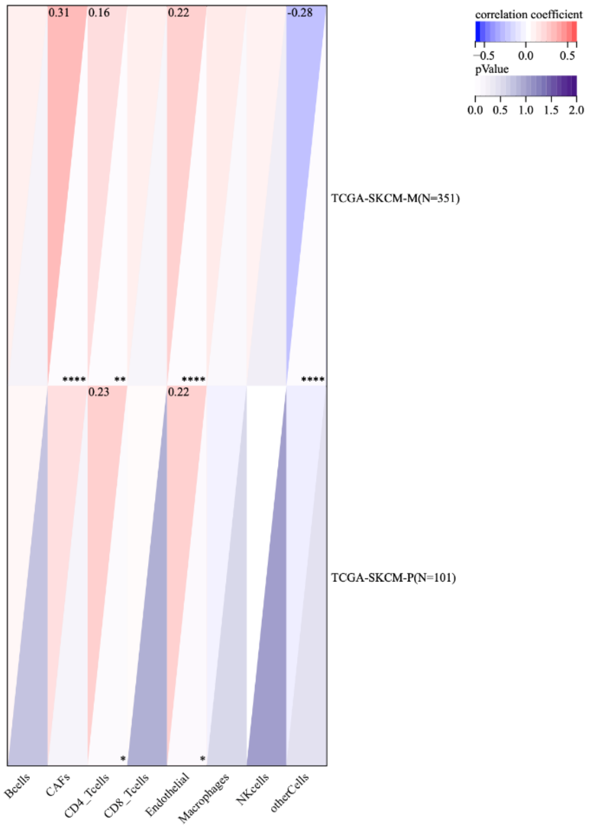

FGFR1

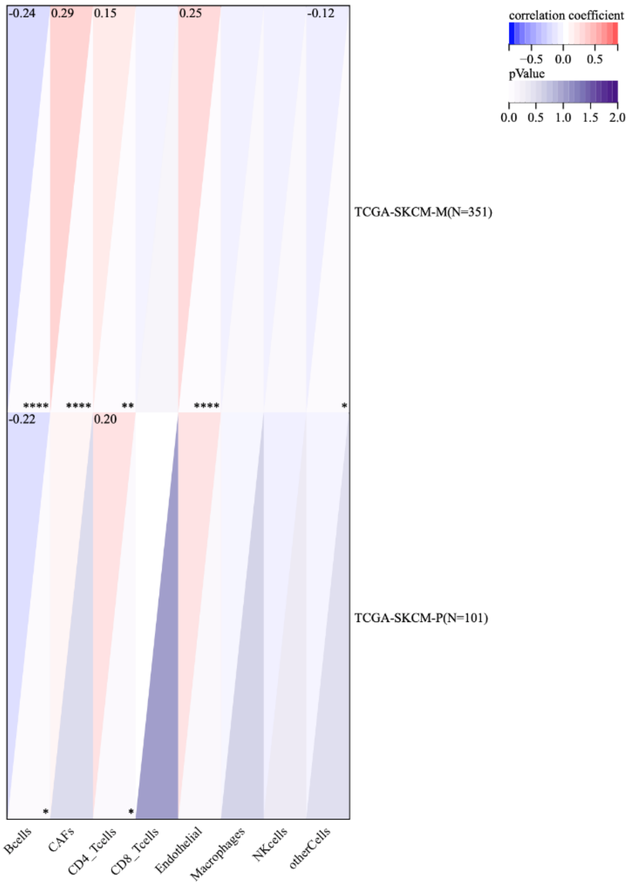

FSTL1

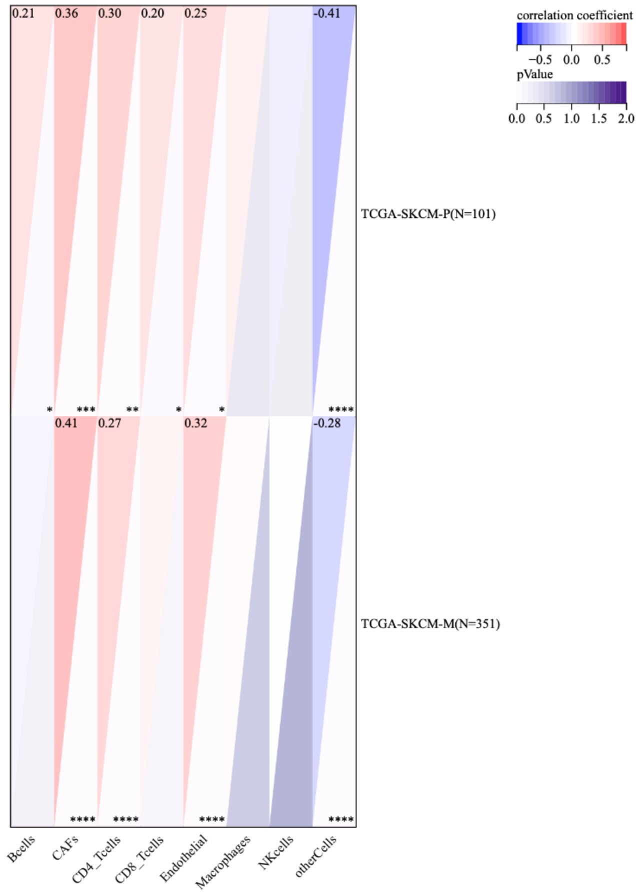

ITGAV

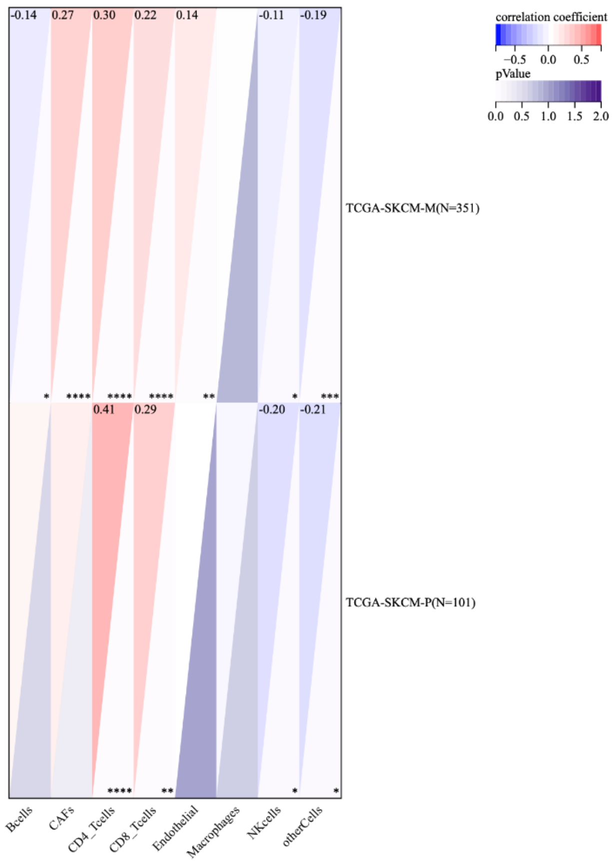

JAG1

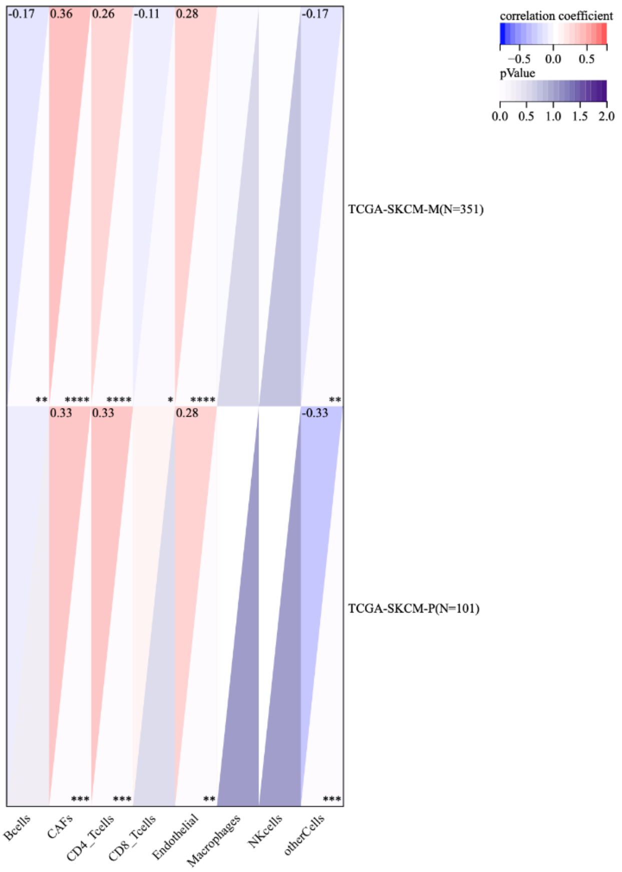

JAG2

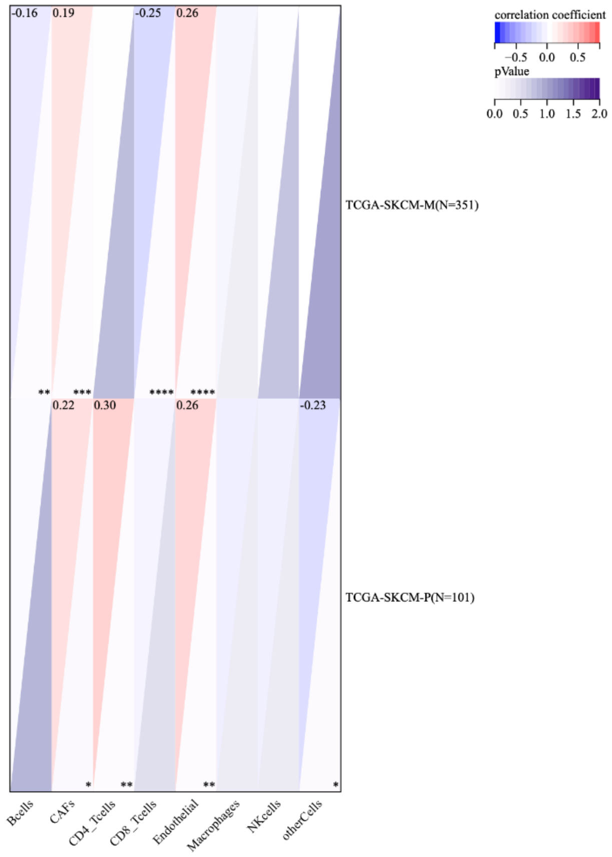

KCNJ8

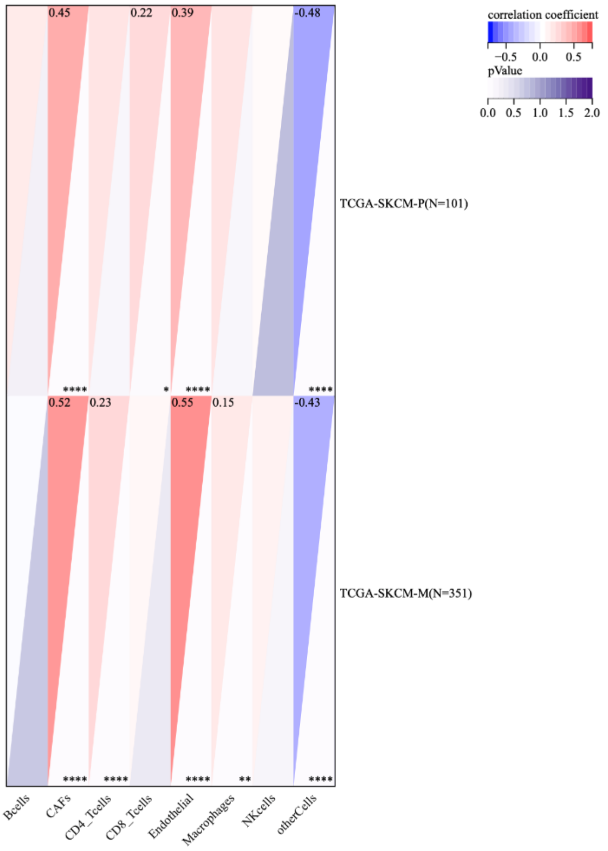

LPL

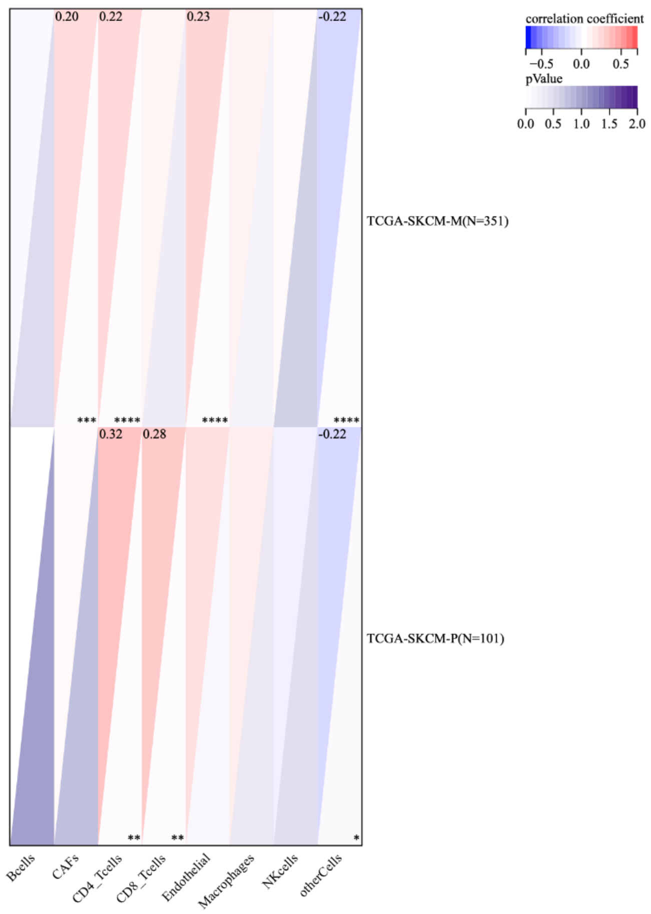

LRPAP1

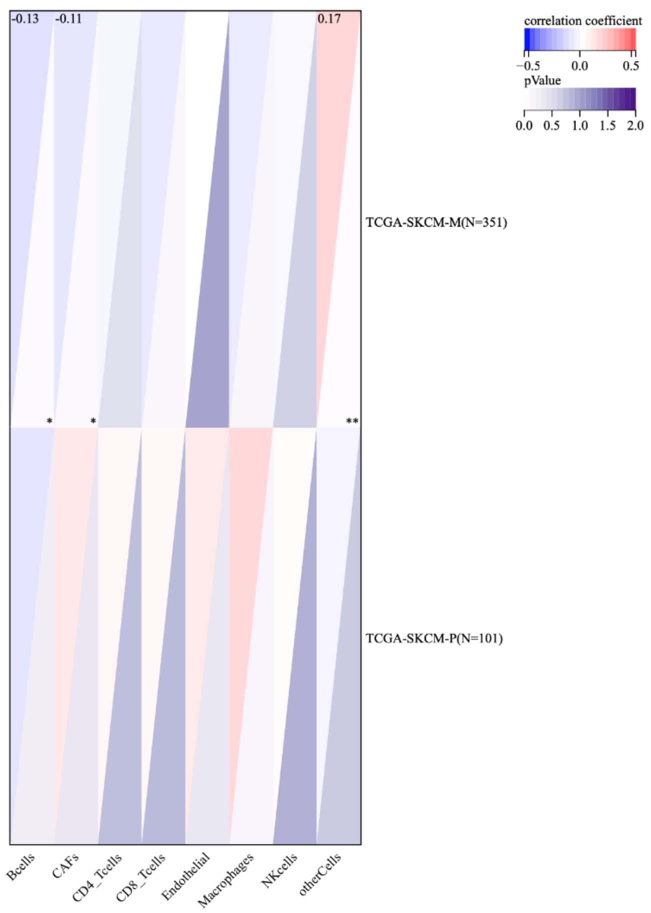

LUM

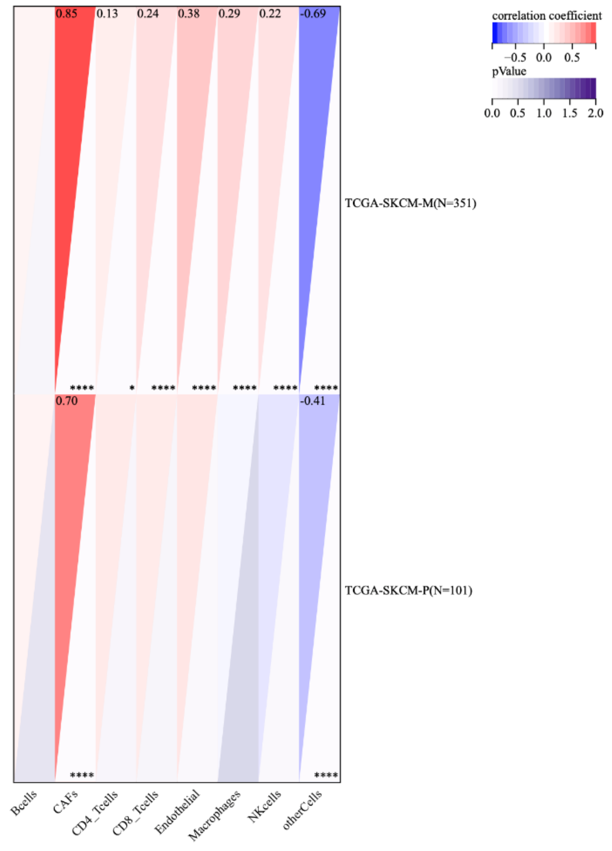

MSX1

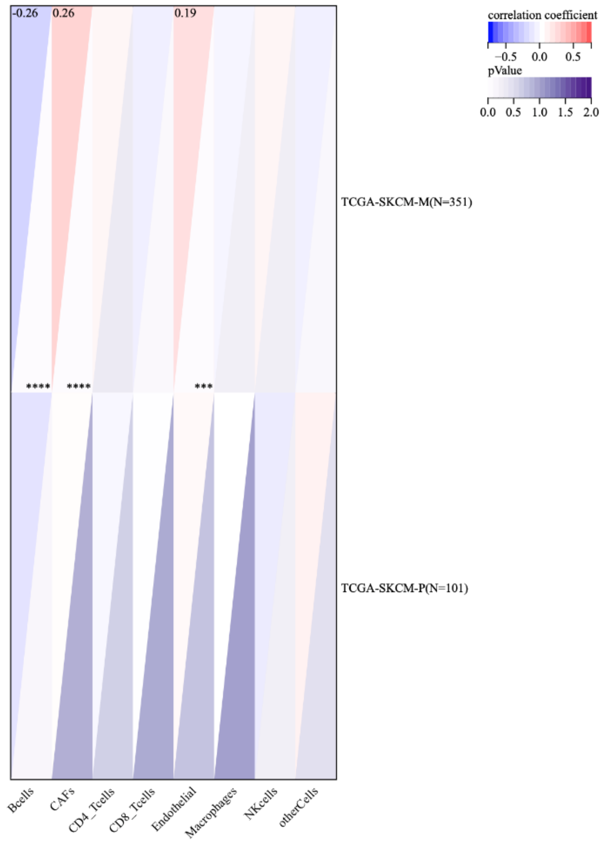

NRP1

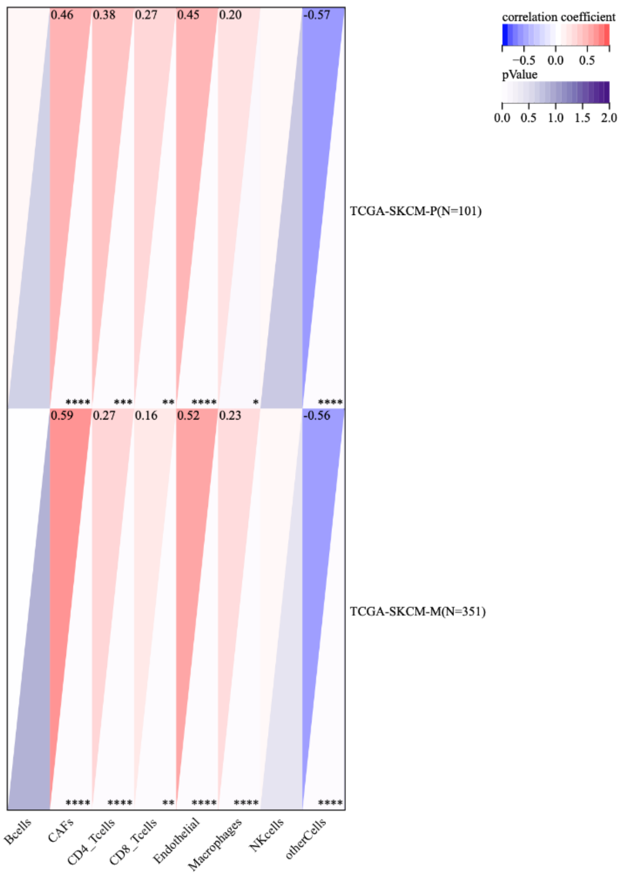

OLR1

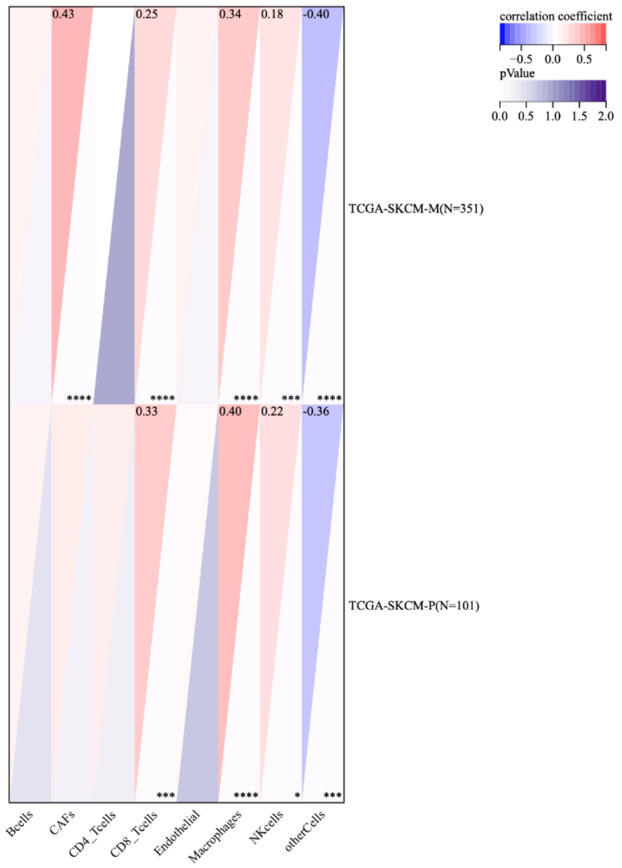

PDGFA

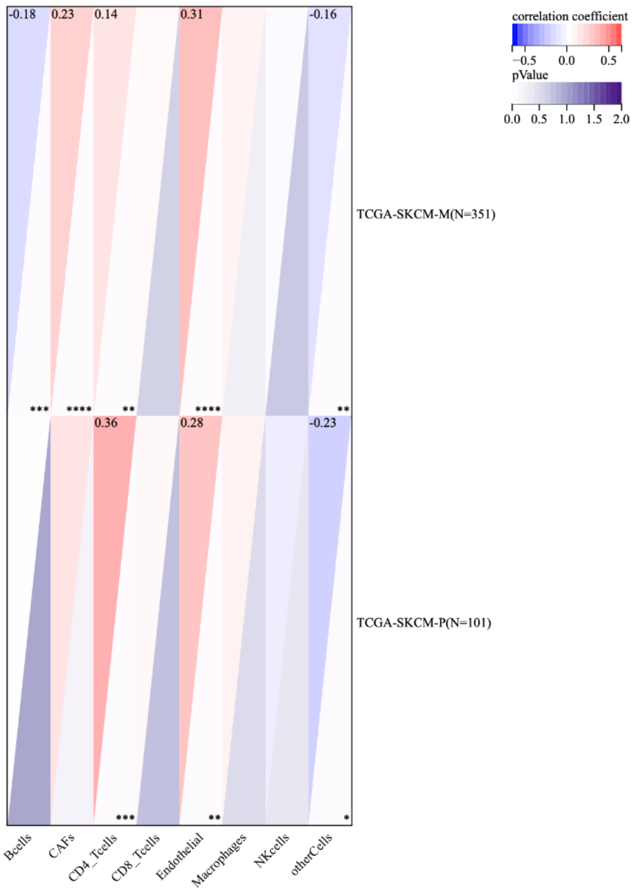

PF4

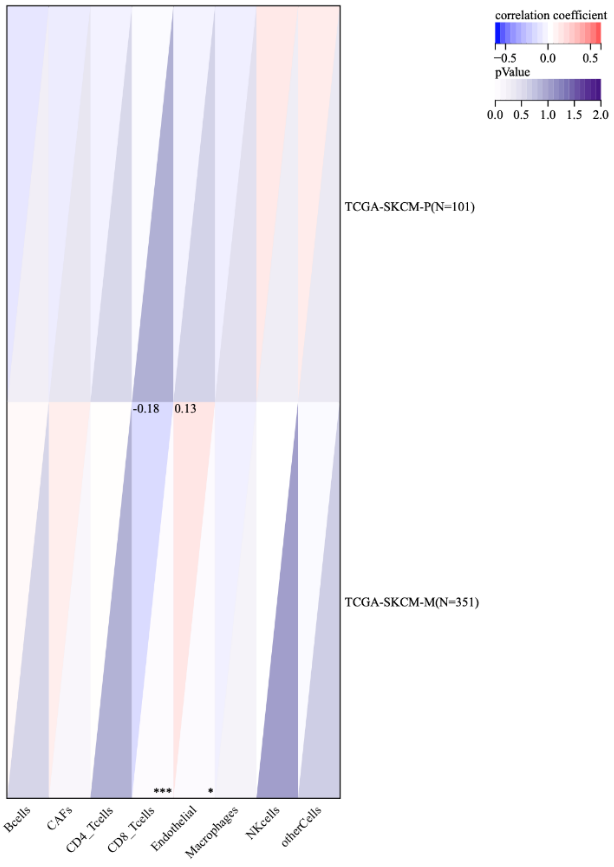

PGLYRP1

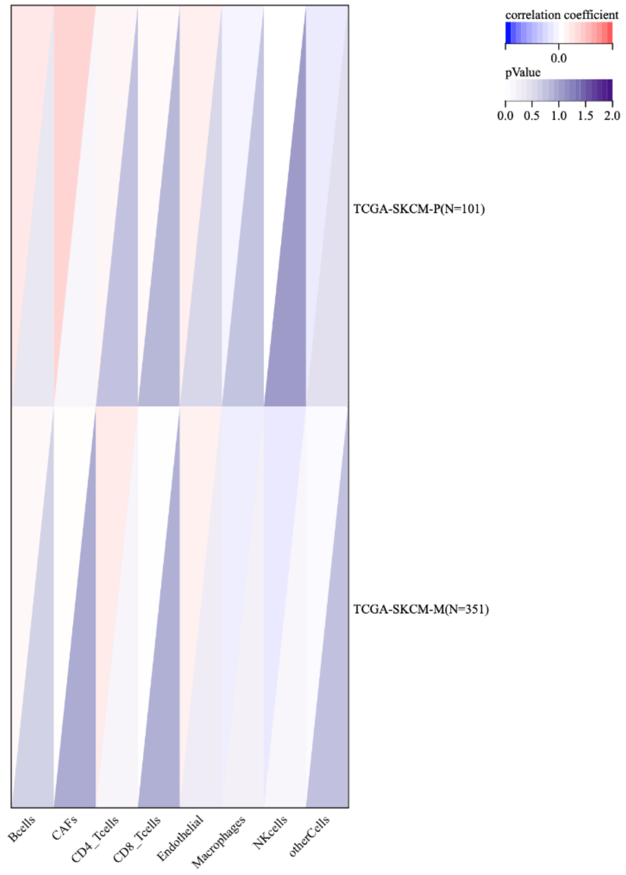

POSTN

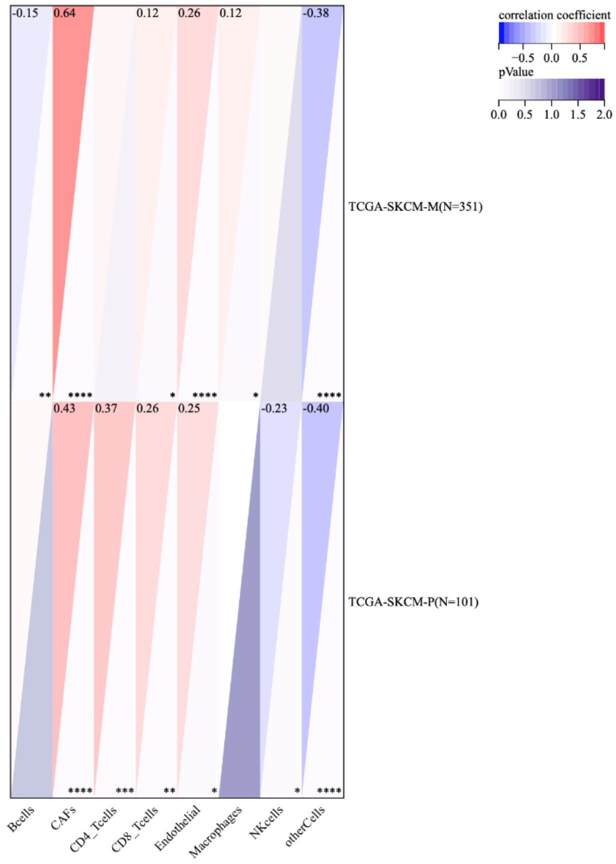

PRG2

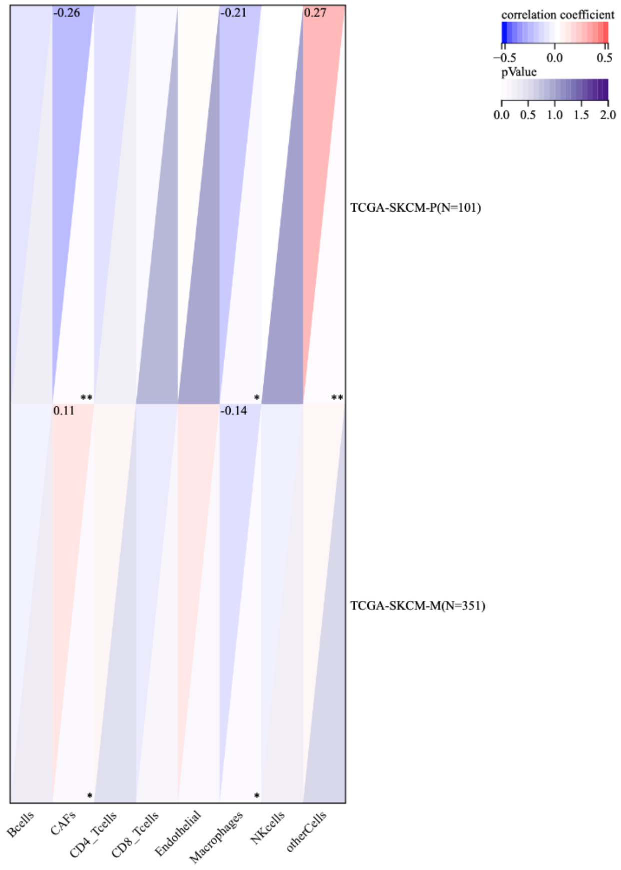

PTK

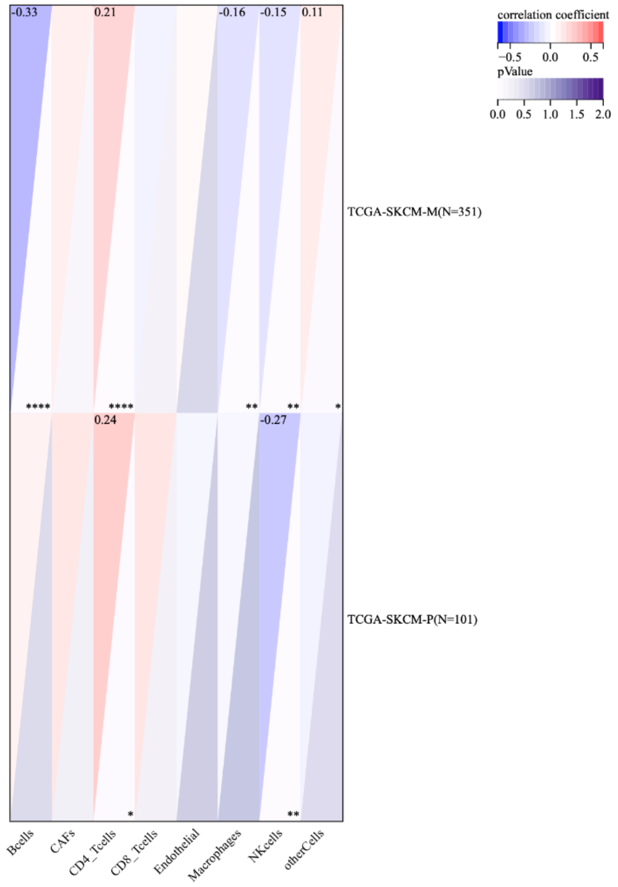

S100A4

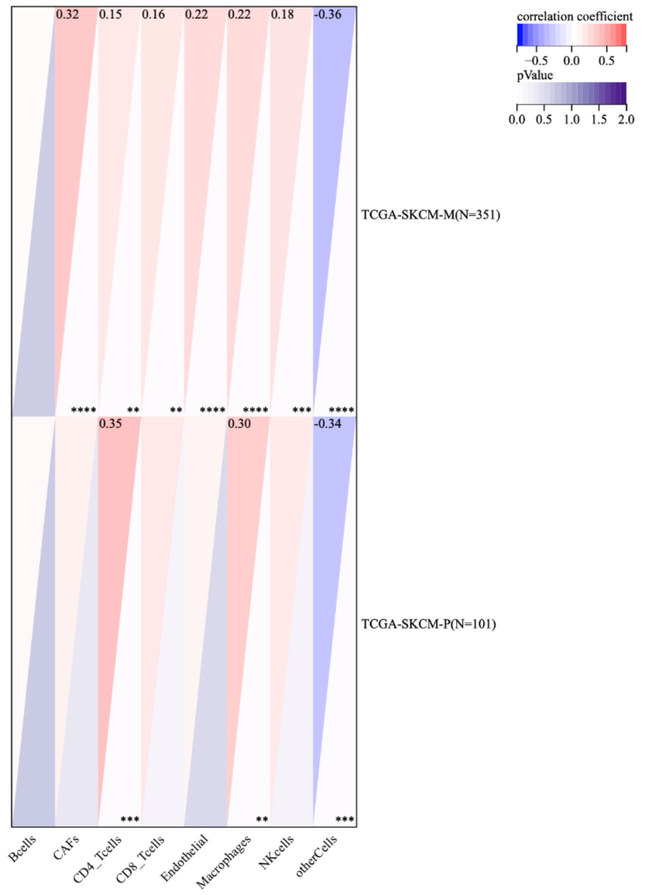

SERPINA5

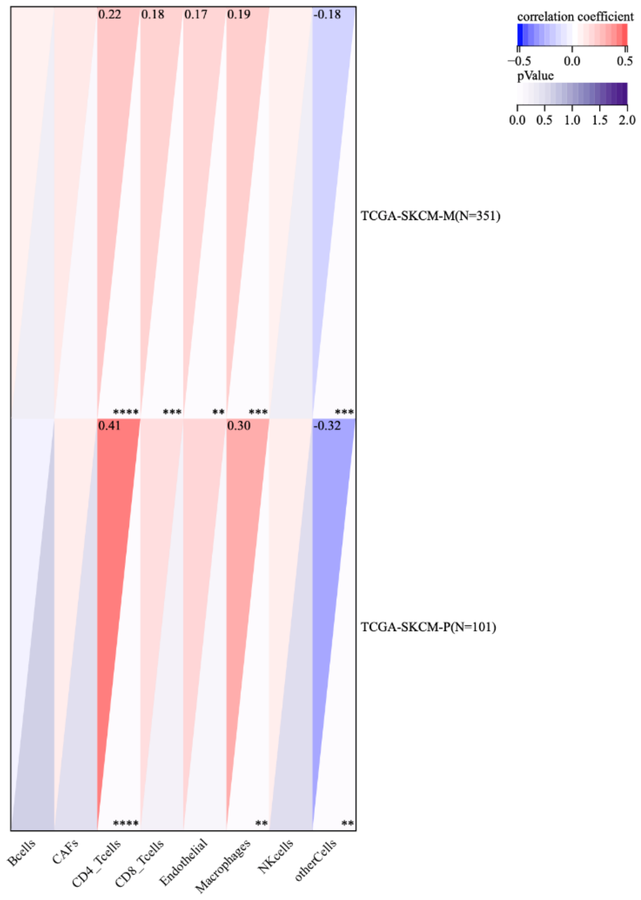

SLCO2A1

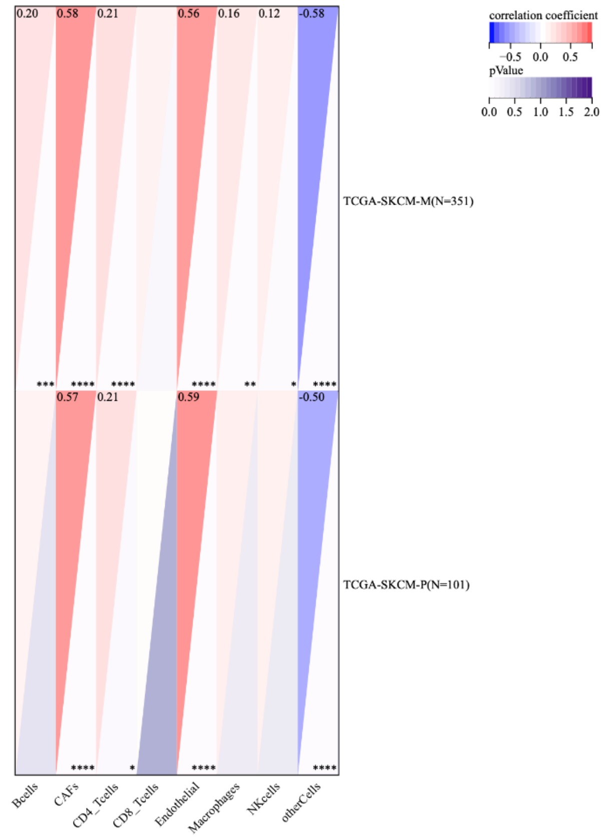

SPP1

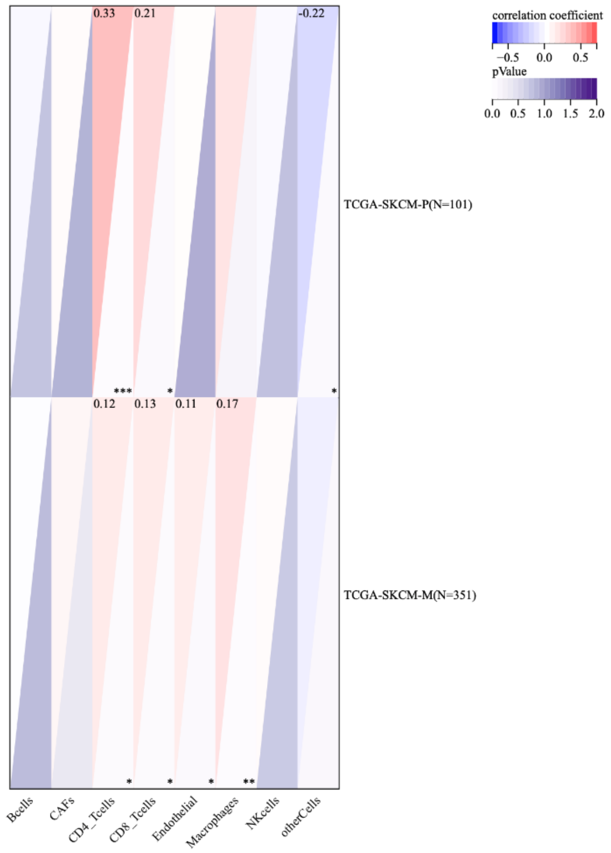

STC1

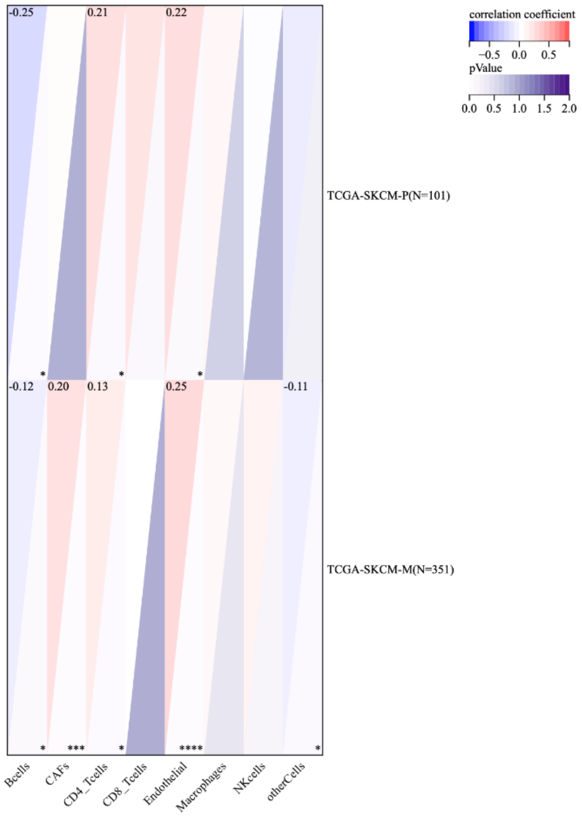

THBD

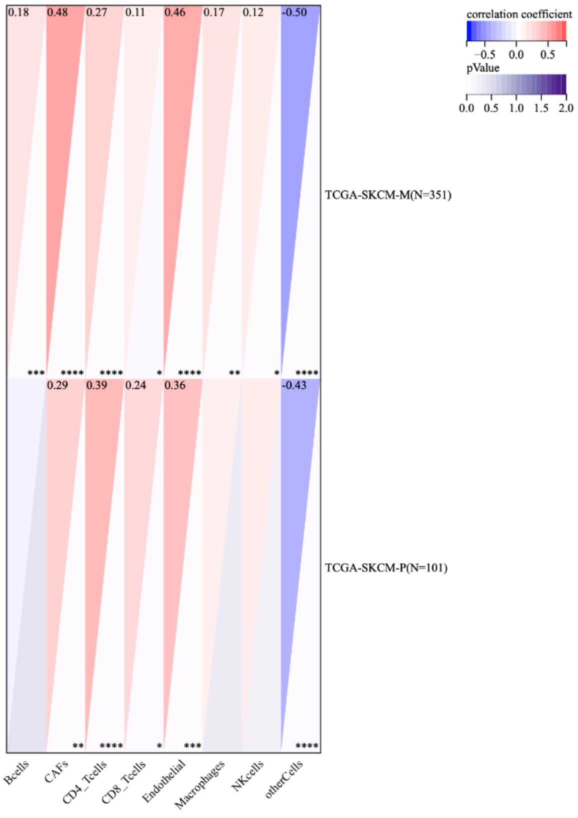

TIMP1

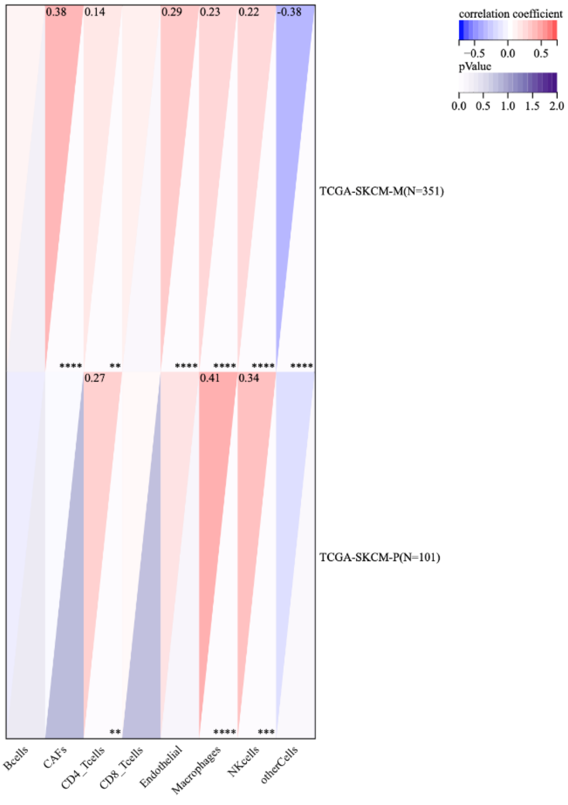

TNFRSF21

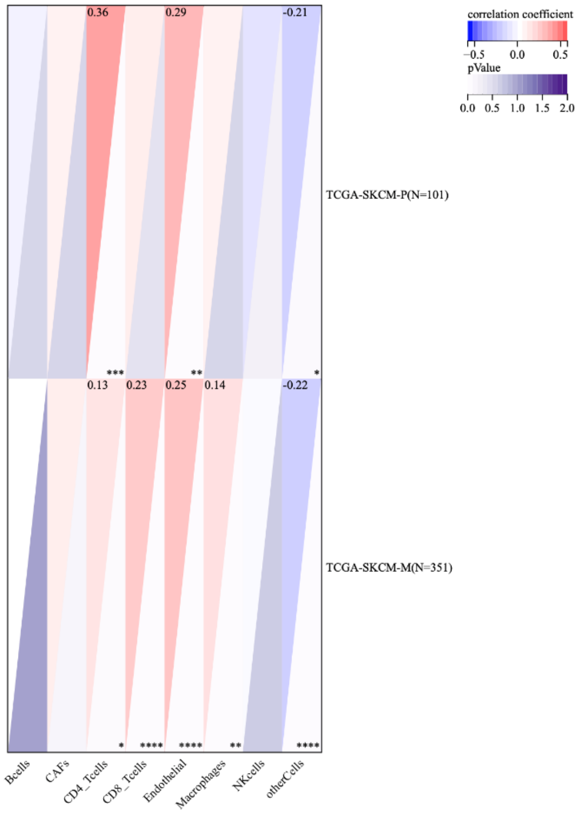

VAV2

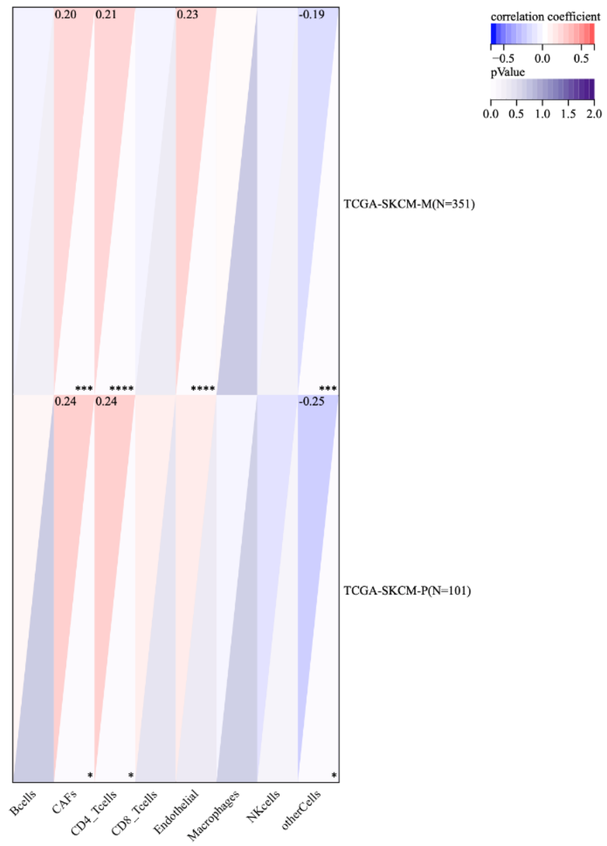

VCAN

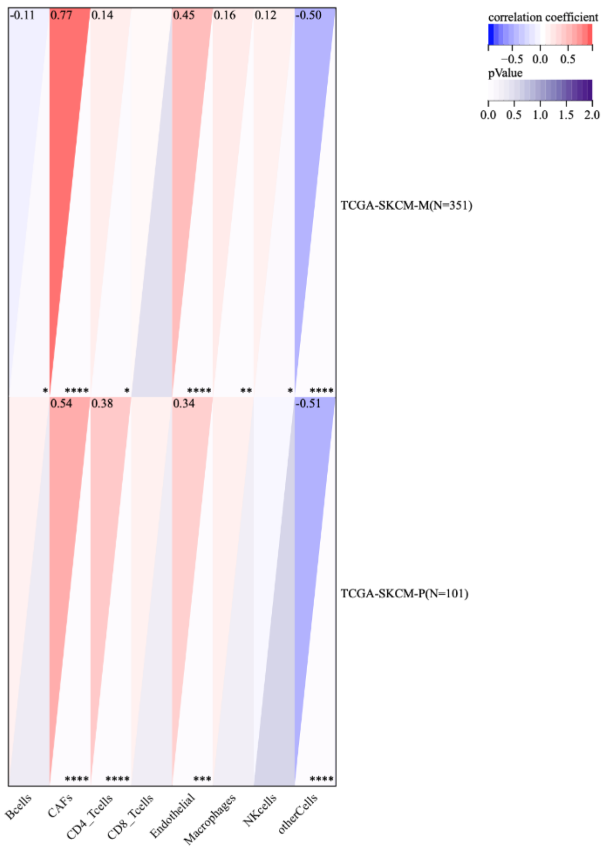

VEGFA

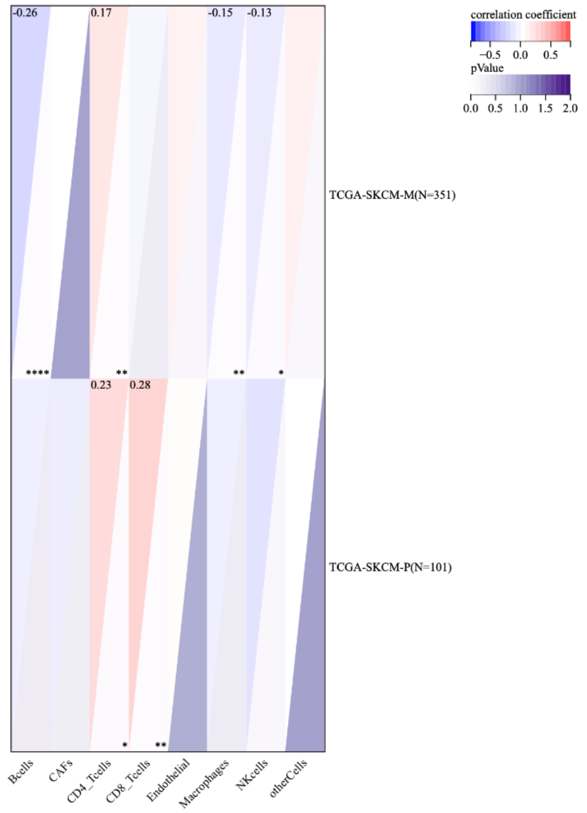

VTN

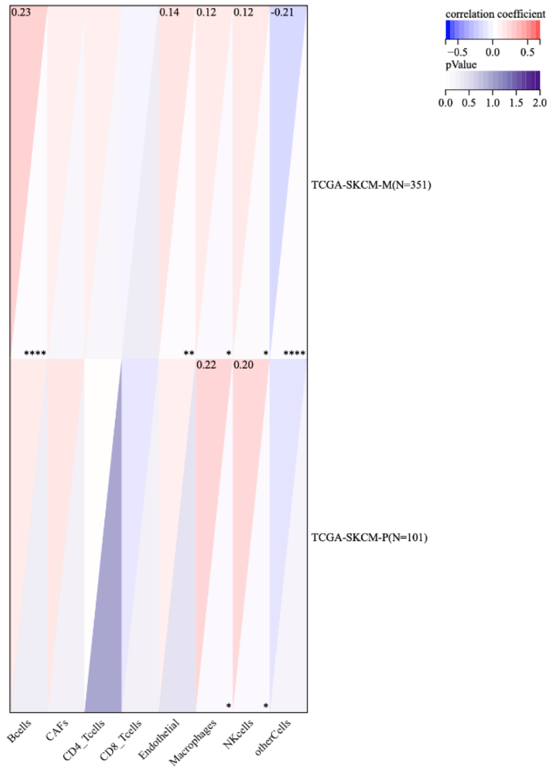

Figure S8. Spearman's correlations between the expression of ARGs and the infiltration in different immune cells in primary and metastatic skin melanoma, using EPIC. Asterisks denote statistical significance: *, p<0.05; ****, p<0.001.

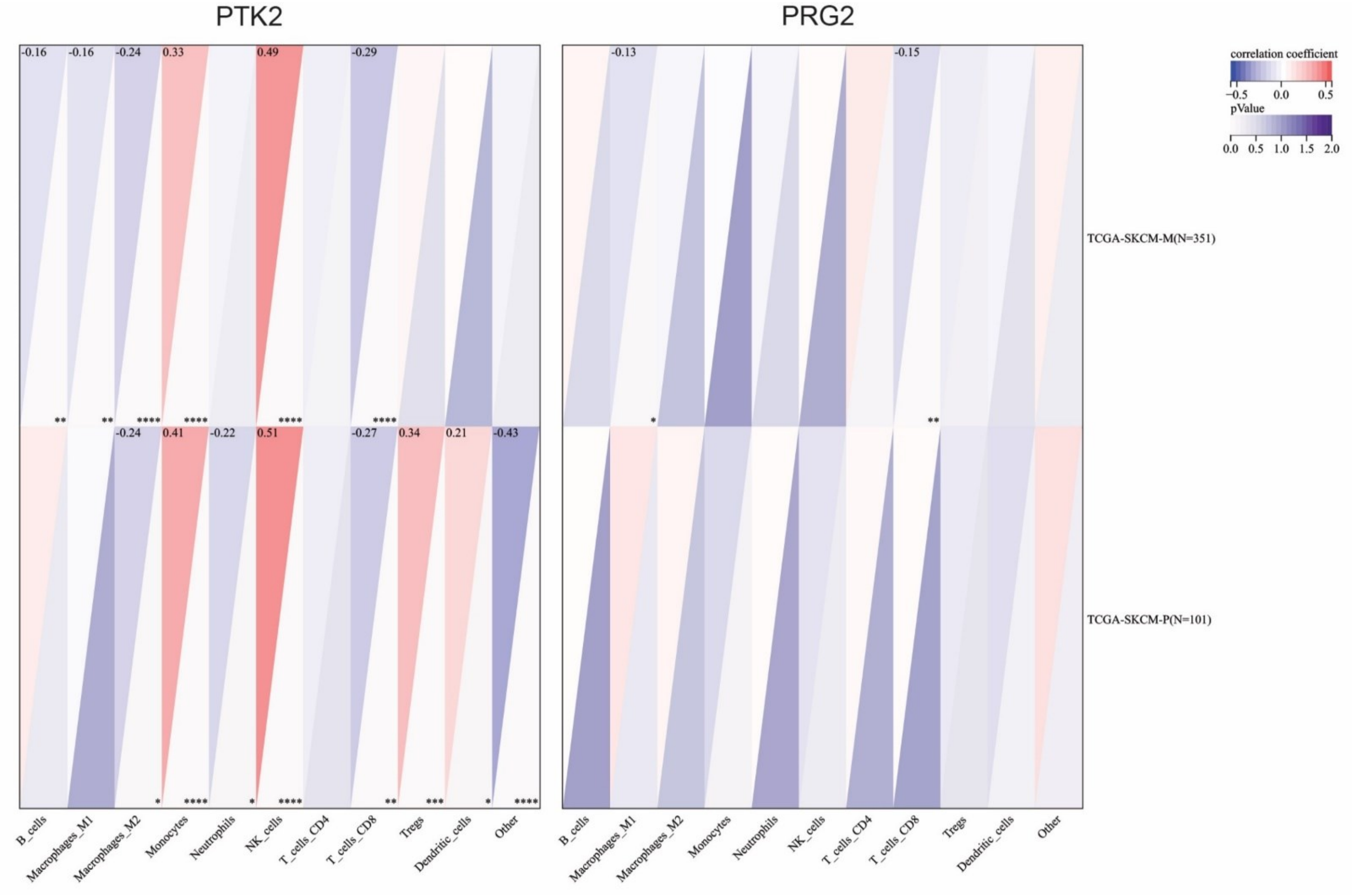

Figure S9 Spearman's correlation analysis between the expression of *PTK2* (or *PRG2*) and immune cell populations in primary (TCGA-SCKM-P) and metastatic skin melanoma (TCGA-SCKM-M), respectively, using QUANTISEC. Asterisks denote statistical significance: *, p<0.05; **, p<0.01; ***, p<0.005; ****, p<0.001.

APOH

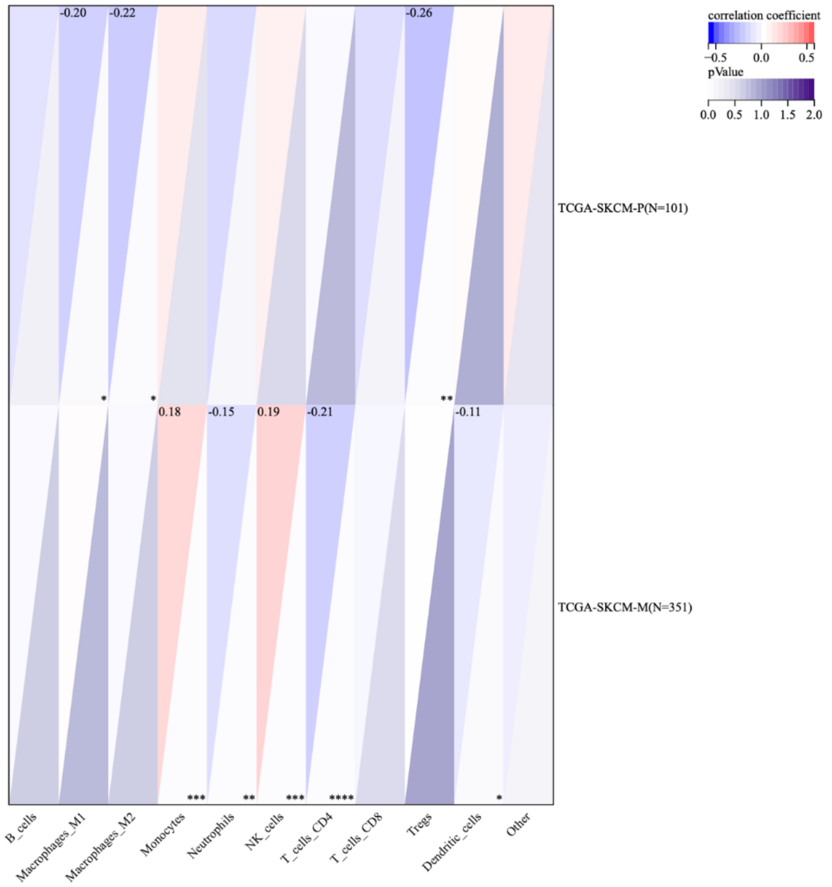
correlation coefficient
−0.5 0.0 0.5
pValue
0.0 0.5 1.0 1.5 2.0
TCGA-SKCM-P(N=101)
TCGA-SKCM-M(N=351)
-0.20
-0.22
-0.26
0.18
-0.15
0.19
-0.21
-0.11
B_cells
Macrophages_M1
Macrophages_M2
Monocytes
Neutrophils
NK_cells
T_cells_CD4
T_cells_CD8
Tregs
Dendritic_cells
Other

APP

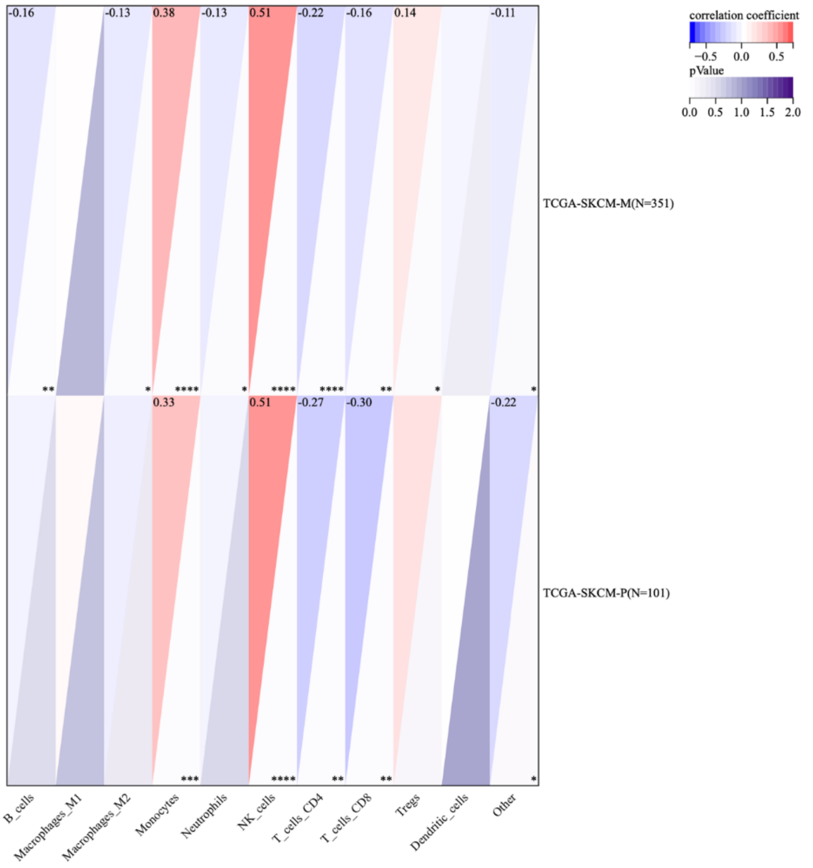
correlation coefficient
−0.5 0.0 0.5
pValue
0.0 0.5 1.0 1.5 2.0
TCGA-SKCM-M(N=351)
TCGA-SKCM-P(N=101)
-0.16
-0.13
0.38
-0.13
0.51
-0.22
-0.16
0.14
-0.11
0.33
0.51
-0.27
-0.30
-0.22
B_cells
Macrophages_M1
Macrophages_M2
Monocytes
Neutrophils
NK_cells
T_cells_CD4
T_cells_CD8
Tregs
Dendritic_cells
Other

CCND2

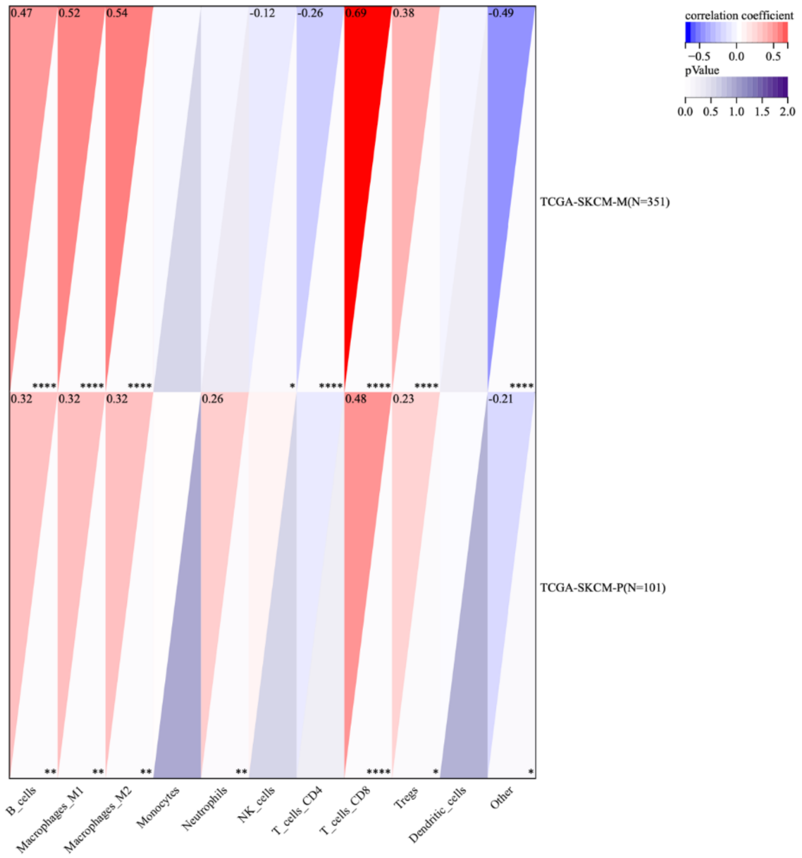

COL3A1

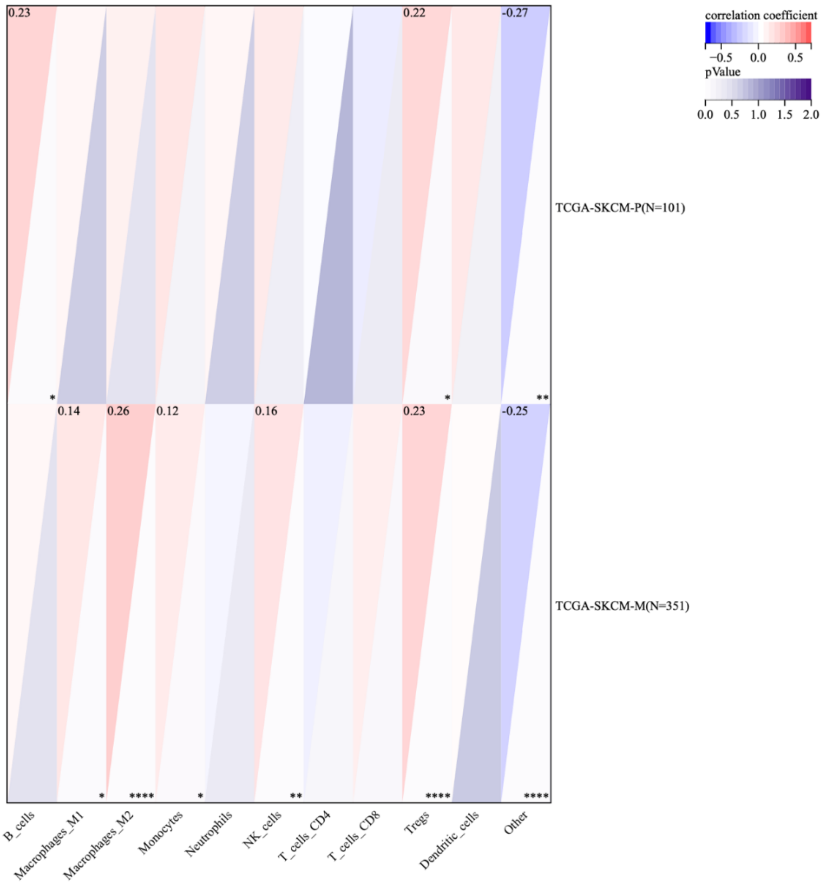

COL5A2

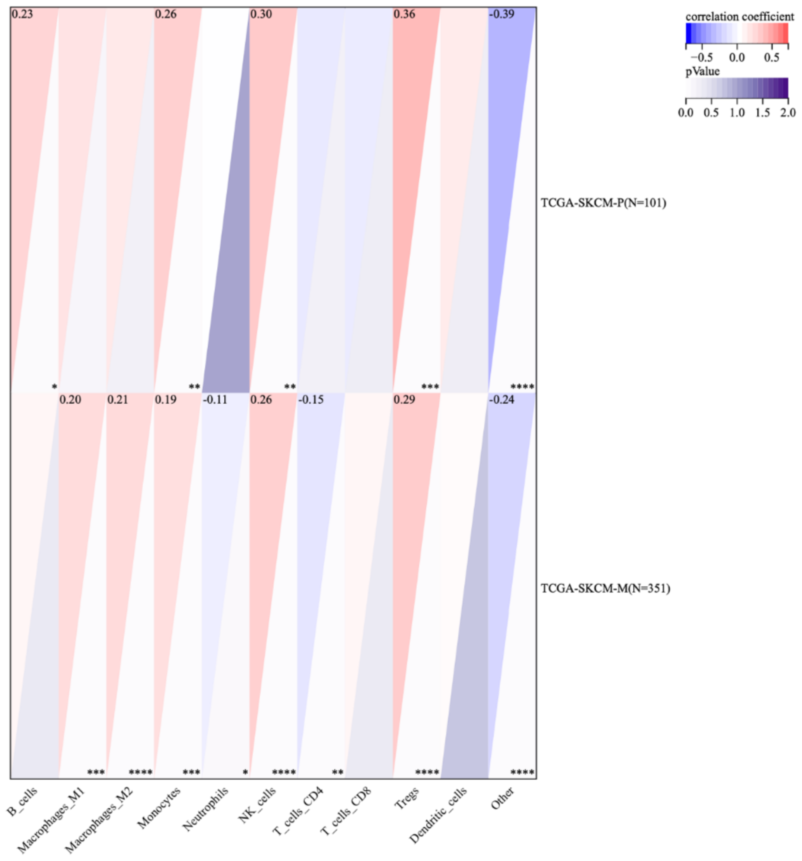

CXCL6

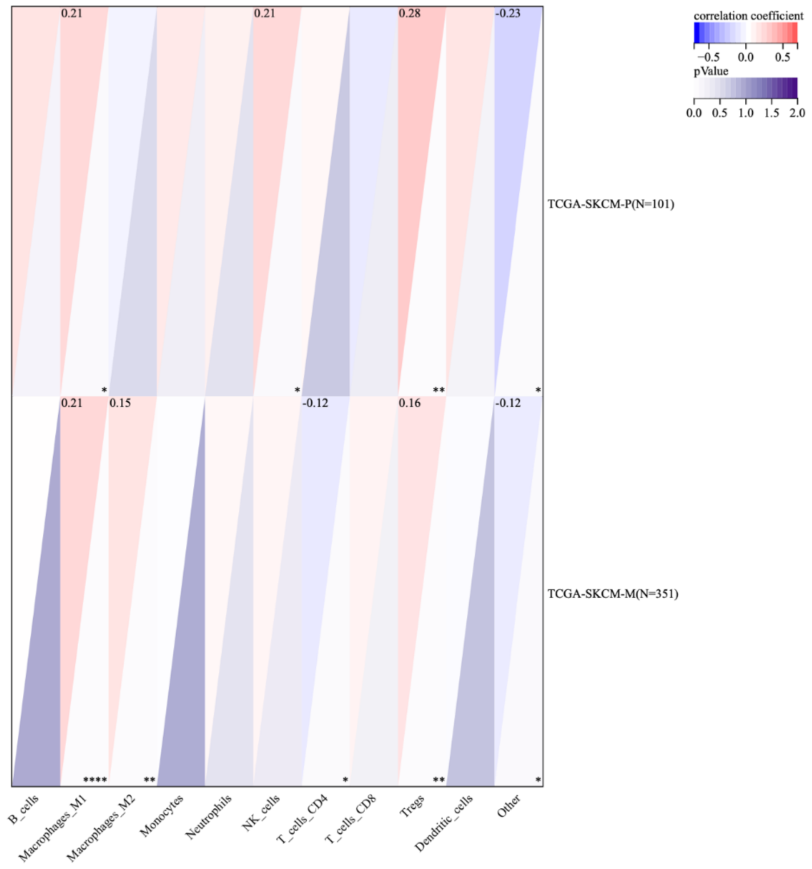

FGFR1

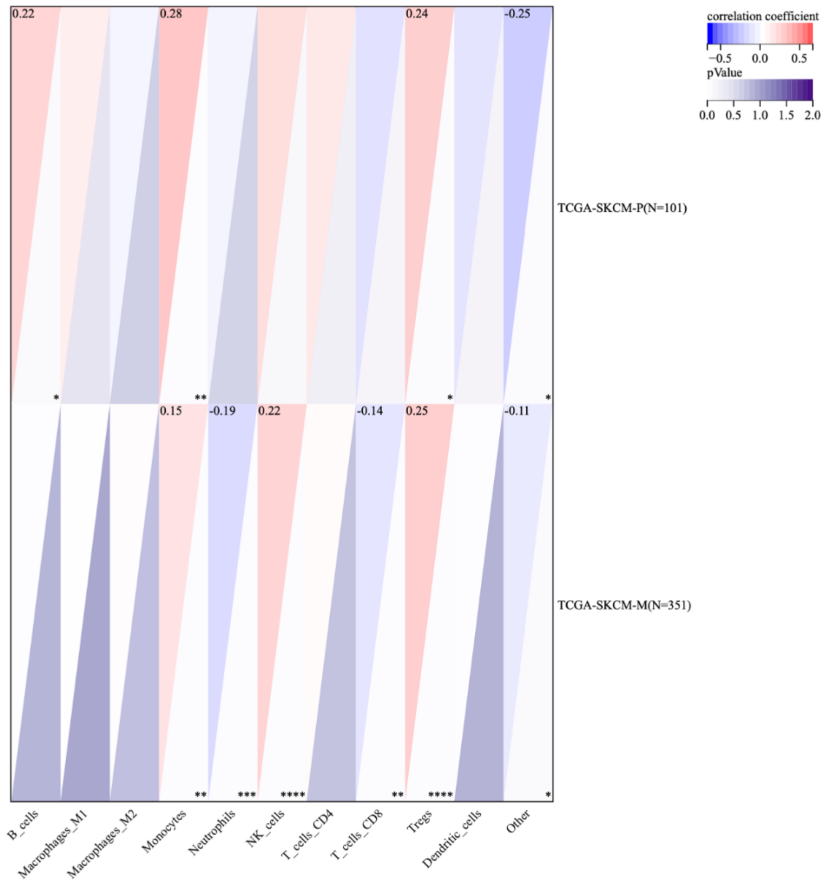

correlation coefficient
−0.5 0.0 0.5
pValue
0.0 0.5 1.0 1.5 2.0
TCGA-SKCM-P(N=101)
TCGA-SKCM-M(N=351)
0.22
0.28
0.24
-0.25
0.15
-0.19
0.22
-0.14
0.25
-0.11
B_cells
Macrophages_M1
Macrophages_M2
Monocytes
Neutrophils
NK_cells
T_cells_CD4
T_cells_CD8
Tregs
Dendritic_cells
Other

FSTL1

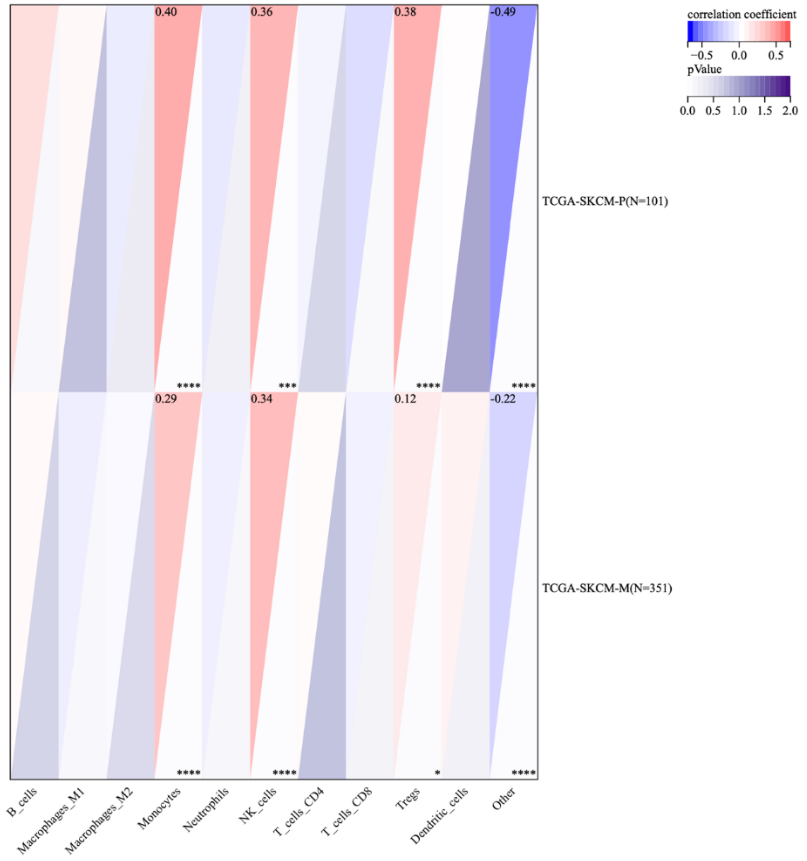

correlation coefficient
−0.5 0.0 0.5
pValue
0.0 0.5 1.0 1.5 2.0
TCGA-SKCM-P(N=101)
TCGA-SKCM-M(N=351)
0.40
0.36
0.38
-0.49
0.29
0.34
0.12
-0.22
B_cells
Macrophages_M1
Macrophages_M2
Monocytes
Neutrophils
NK_cells
T_cells_CD4
T_cells_CD8
Tregs
Dendritic_cells
Other

ITGAV

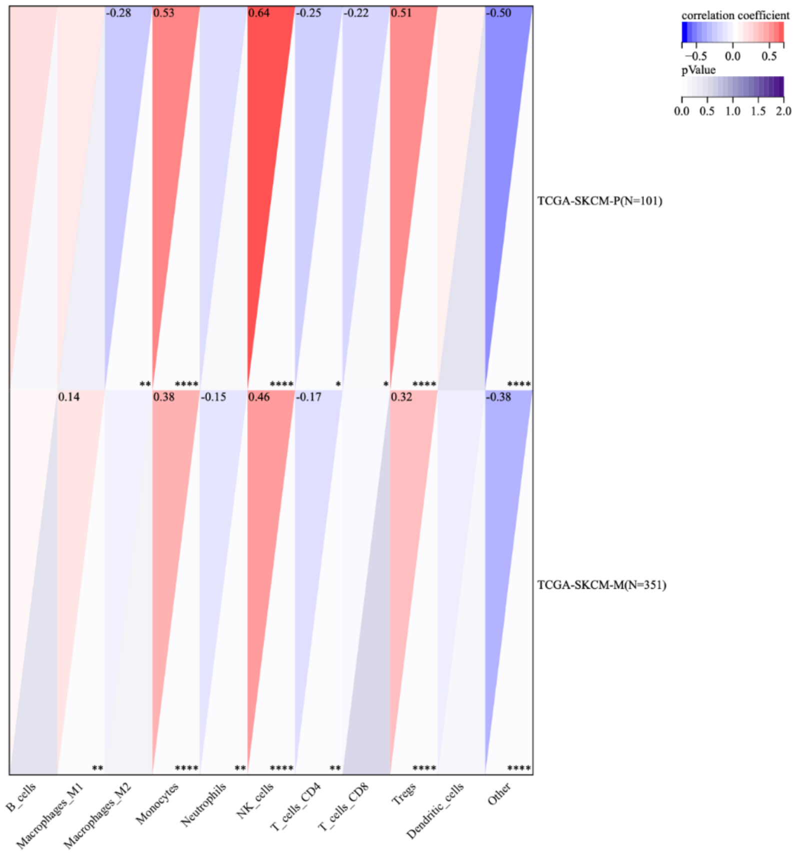
correlation coefficient
−0.5 0.0 0.5
pValue
0.0 0.5 1.0 1.5 2.0
TCGA-SKCM-P(N=101)
TCGA-SKCM-M(N=351)
-0.28 0.53 0.64 -0.25 -0.22 0.51 -0.50
** **** **** * * **** ****
0.14 0.38 -0.15 0.46 -0.17 0.32 -0.38
** **** ** **** ** **** ****
B_cells
Macrophages_M1
Macrophages_M2
Monocytes
Neutrophils
NK_cells
T_cells_CD4
T_cells_CD8
Tregs
Dendritic_cells
Other

JAG1

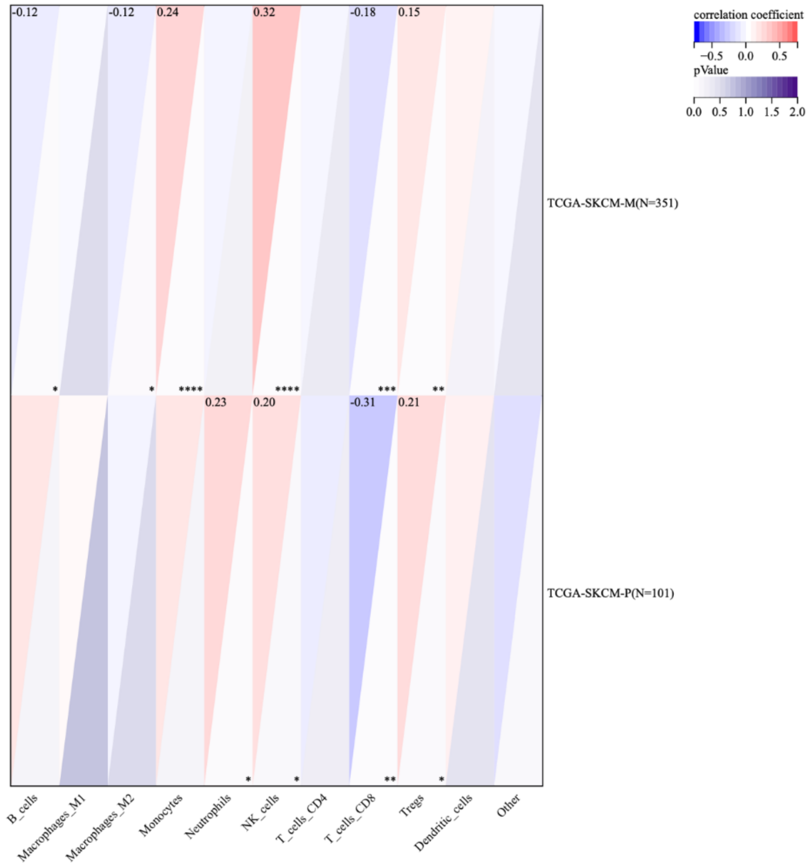
correlation coefficient
−0.5 0.0 0.5
pValue
0.0 0.5 1.0 1.5 2.0
TCGA-SKCM-M(N=351)
TCGA-SKCM-P(N=101)
-0.12 -0.12 0.24 0.32 -0.18 0.15
* * **** **** *** **
0.23 0.20 -0.31 0.21
* * ** *
B_cells
Macrophages_M1
Macrophages_M2
Monocytes
Neutrophils
NK_cells
T_cells_CD4
T_cells_CD8
Tregs
Dendritic_cells
Other

JAG2

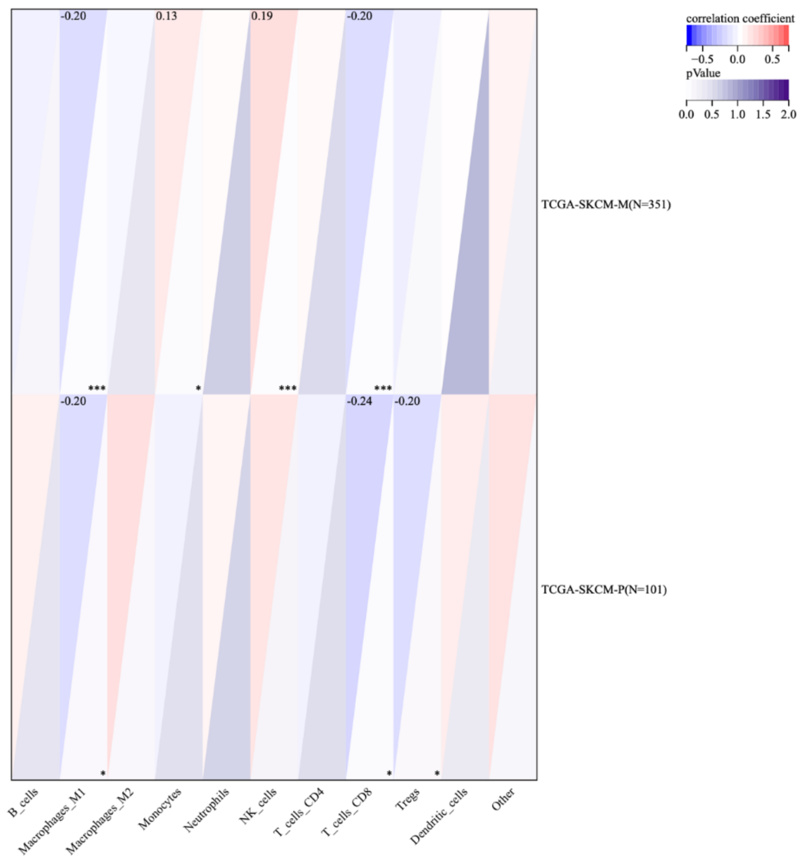
correlation coefficient
−0.5 0.0 0.5
pValue
0.0 0.5 1.0 1.5 2.0
-0.20
0.13
0.19
-0.20
***
*
***
***
-0.20
-0.24
-0.20
*
*
*
TCGA-SKCM-M(N=351)
TCGA-SKCM-P(N=101)
B_cells
Macrophages_M1
Macrophages_M2
Monocytes
Neutrophils
NK_cells
T_cells_CD4
T_cells_CD8
Tregs
Dendritic_cells
Other

KCNJ8

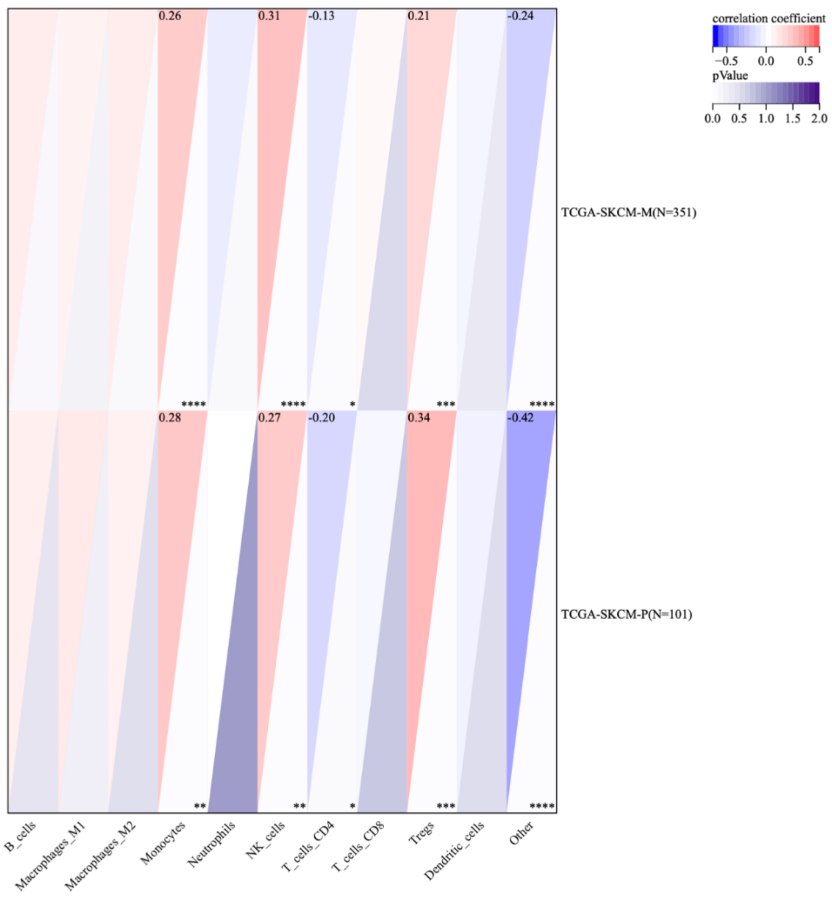
correlation coefficient
−0.5 0.0 0.5
pValue
0.0 0.5 1.0 1.5 2.0
0.26
0.31
-0.13
0.21
-0.24
****
****
*
***
****
0.28
0.27
-0.20
0.34
-0.42
**
**
*
***
****
TCGA-SKCM-M(N=351)
TCGA-SKCM-P(N=101)
B_cells
Macrophages_M1
Macrophages_M2
Monocytes
Neutrophils
NK_cells
T_cells_CD4
T_cells_CD8
Tregs
Dendritic_cells
Other

LPL

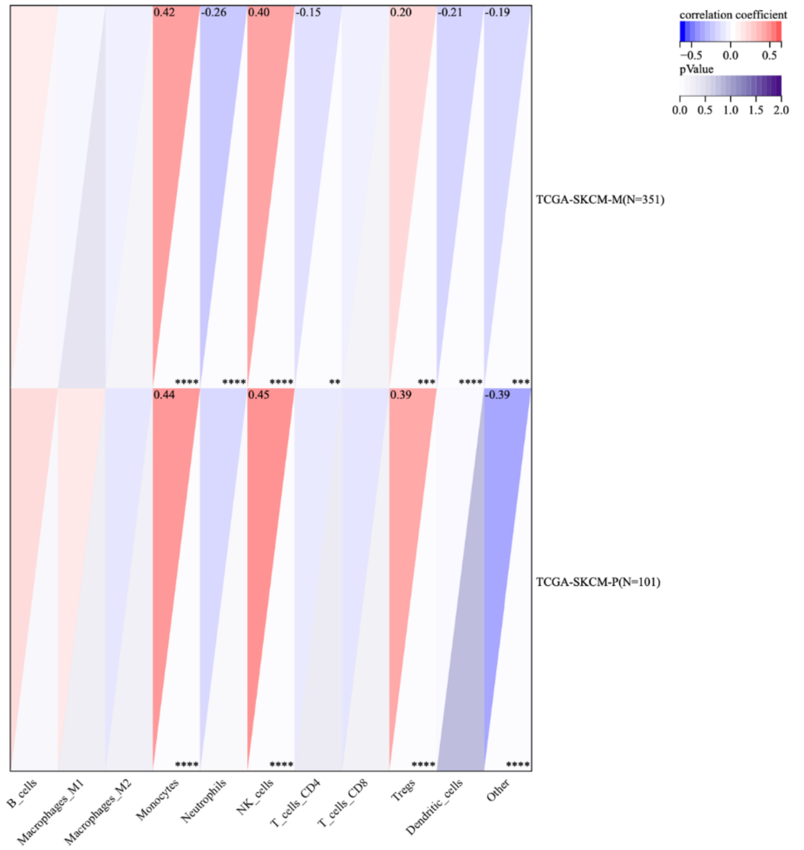

correlation coefficient
−0.5 0.0 0.5
pValue
0.0 0.5 1.0 1.5 2.0
0.42 -0.26 0.40 -0.15 0.20 -0.21 -0.19
**** **** **** ** *** **** ***
TCGA-SKCM-M(N=351)
0.44 0.45 0.39 -0.39
**** **** **** ****
TCGA-SKCM-P(N=101)
B_cells
Macrophages_M1
Macrophages_M2
Monocytes
Neutrophils
NK_cells
T_cells_CD4
T_cells_CD8
Tregs
Dendritic_cells
Other

LRPAP1

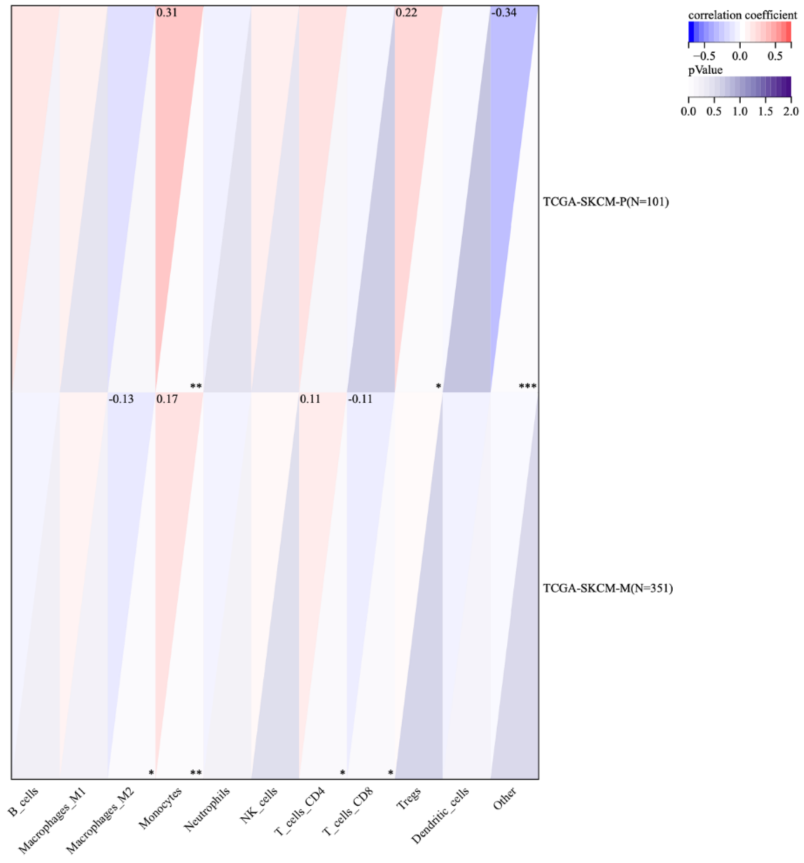

correlation coefficient
−0.5 0.0 0.5
pValue
0.0 0.5 1.0 1.5 2.0
0.31 0.22 -0.34
** * ***
TCGA-SKCM-P(N=101)
-0.13 0.17 0.11 -0.11
* ** * *
TCGA-SKCM-M(N=351)
B_cells
Macrophages_M1
Macrophages_M2
Monocytes
Neutrophils
NK_cells
T_cells_CD4
T_cells_CD8
Tregs
Dendritic_cells
Other

LUM

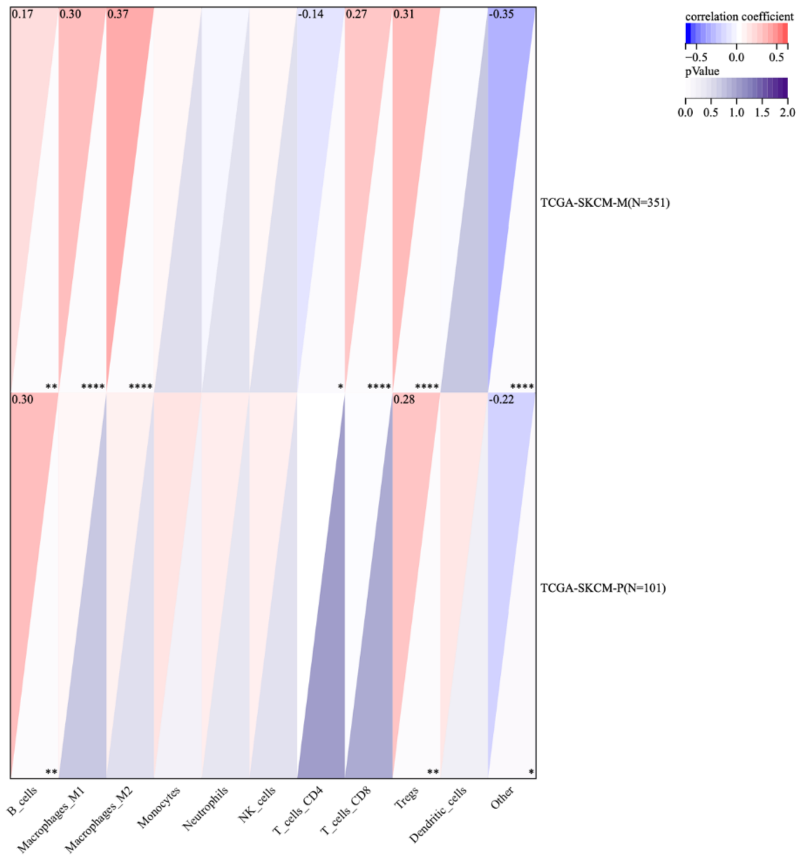
correlation coefficient
−0.5 0.0 0.5
pValue
0.0 0.5 1.0 1.5 2.0
0.17
0.30
0.37
-0.14
0.27
0.31
-0.35
0.30
0.28
-0.22
TCGA-SKCM-M(N=351)
TCGA-SKCM-P(N=101)
B_cells
Macrophages_M1
Macrophages_M2
Monocytes
Neutrophils
NK_cells
T_cells_CD4
T_cells_CD8
Tregs
Dendritic_cells
Other

MSX1

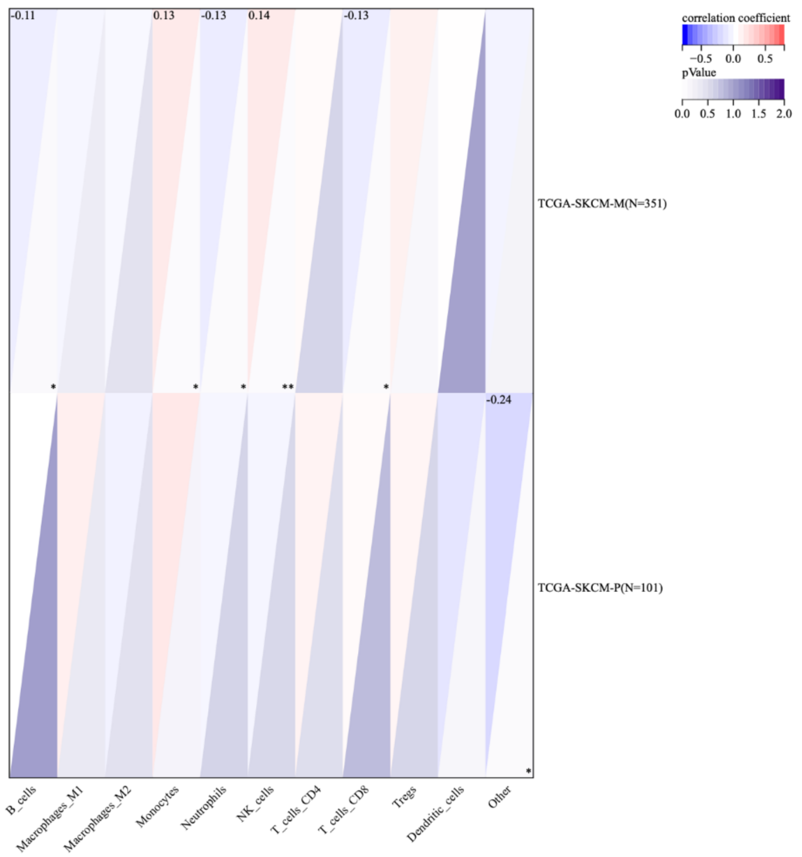
correlation coefficient
−0.5 0.0 0.5
pValue
0.0 0.5 1.0 1.5 2.0
-0.11
0.13
-0.13
0.14
-0.13
-0.24
TCGA-SKCM-M(N=351)
TCGA-SKCM-P(N=101)
B_cells
Macrophages_M1
Macrophages_M2
Monocytes
Neutrophils
NK_cells
T_cells_CD4
T_cells_CD8
Tregs
Dendritic_cells
Other

NRP1

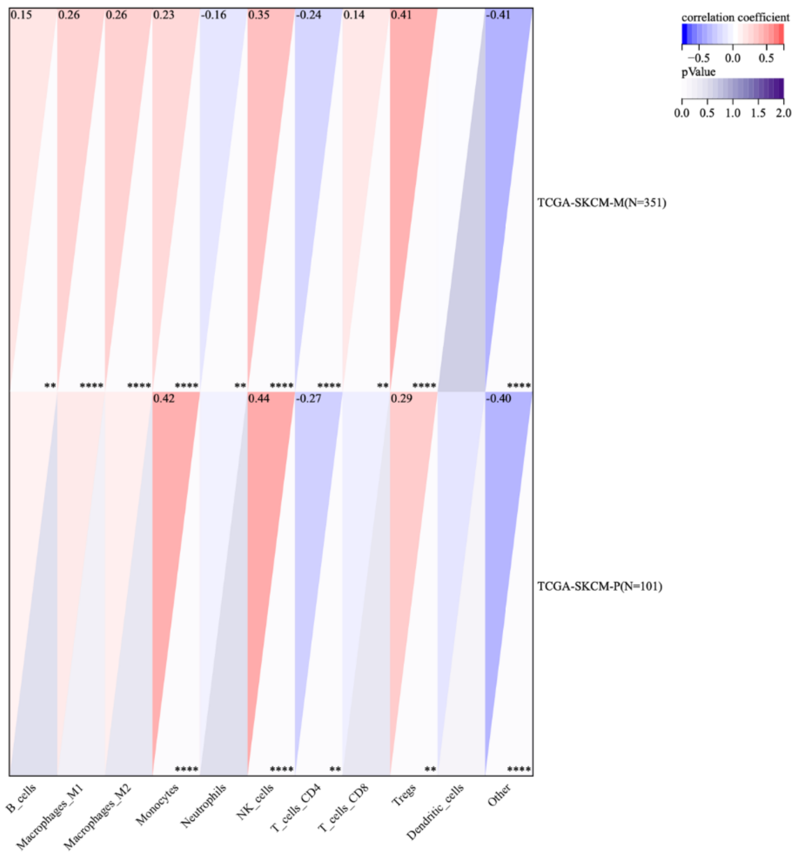

0.15
0.26
0.26
0.23
-0.16
0.35
-0.24
0.14
0.41
-0.41
**
****
****
****
**
****
****
**
****
****
0.42
0.44
-0.27
0.29
-0.40
****
****
**
**
****
TCGA-SKCM-M(N=351)
TCGA-SKCM-P(N=101)
correlation coefficient
−0.5
0.0
0.5
pValue
0.0
0.5
1.0
1.5
2.0
B_cells
Macrophages_M1
Macrophages_M2
Monocytes
Neutrophils
NK_cells
T_cells_CD4
T_cells_CD8
Tregs
Dendritic_cells
Other

OLR1

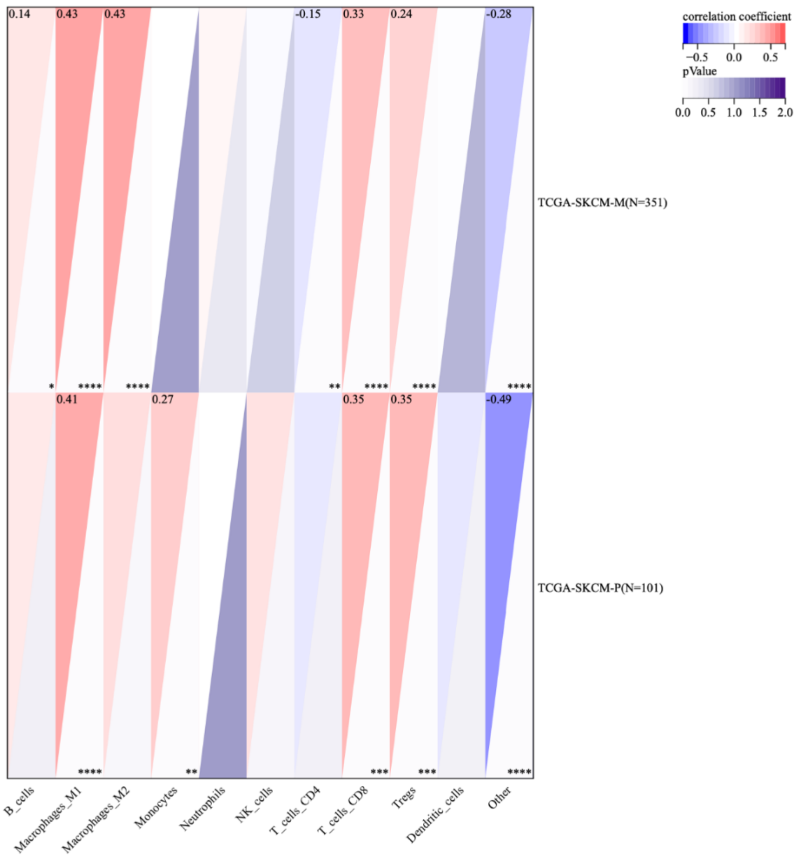

0.14
0.43
0.43
-0.15
0.33
0.24
-0.28
*
****
****
**
****
****
****
0.41
0.27
0.35
0.35
-0.49
****
**
***
***
****
TCGA-SKCM-M(N=351)
TCGA-SKCM-P(N=101)
correlation coefficient
−0.5
0.0
0.5
pValue
0.0
0.5
1.0
1.5
2.0
B_cells
Macrophages_M1
Macrophages_M2
Monocytes
Neutrophils
NK_cells
T_cells_CD4
T_cells_CD8
Tregs
Dendritic_cells
Other

PDGFA

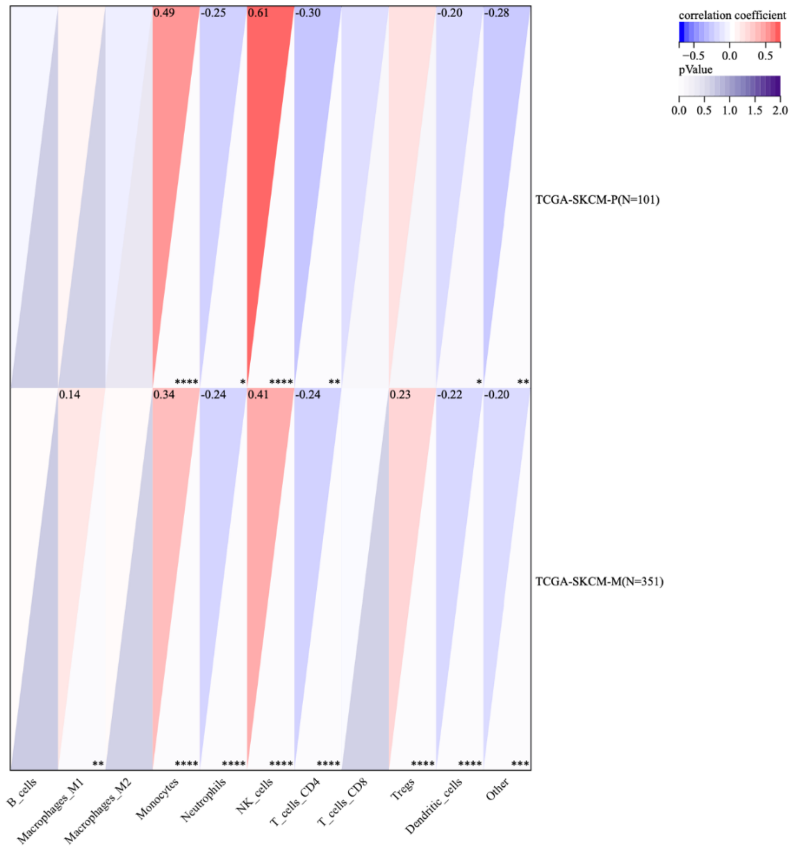
correlation coefficient
−0.5 0.0 0.5
pValue
0.0 0.5 1.0 1.5 2.0
TCGA-SKCM-P(N=101)
TCGA-SKCM-M(N=351)
0.49 -0.25 0.61 -0.30 -0.20 -0.28
0.14 0.34 -0.24 0.41 -0.24 0.23 -0.22 -0.20
B_cells
Macrophages_M1
Macrophages_M2
Monocytes
Neutrophils
NK_cells
T_cells_CD4
T_cells_CD8
Tregs
Dendritic_cells
Other

PF4

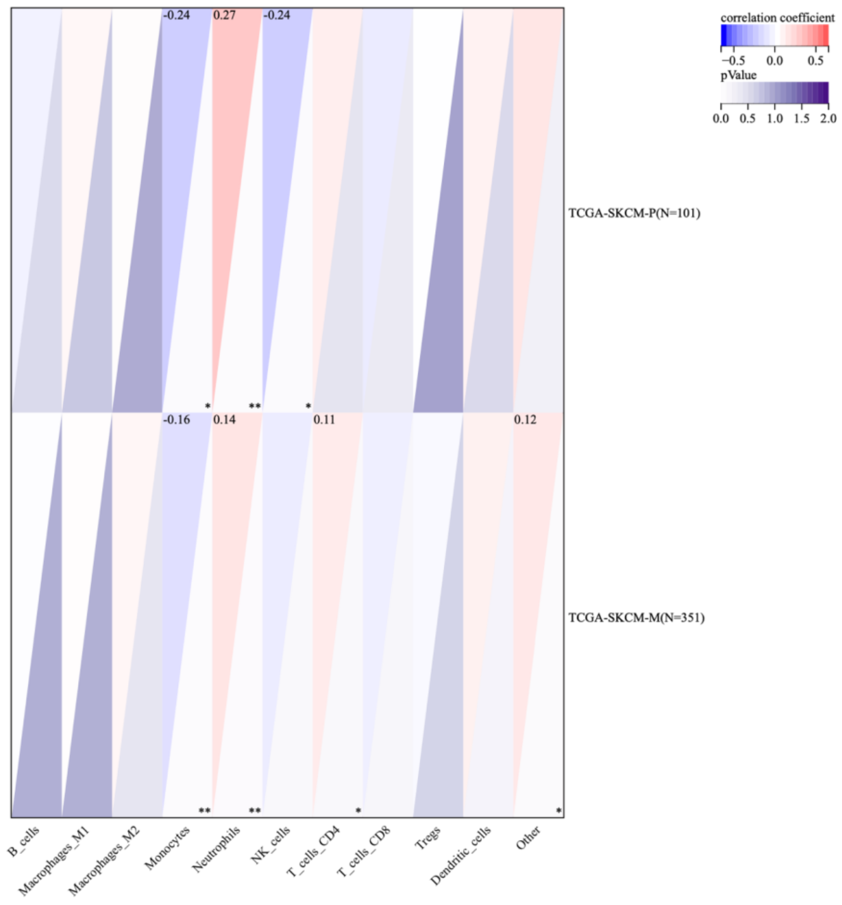
correlation coefficient
−0.5 0.0 0.5
pValue
0.0 0.5 1.0 1.5 2.0
TCGA-SKCM-P(N=101)
TCGA-SKCM-M(N=351)
-0.24 0.27 -0.24
-0.16 0.14 0.11 0.12
B_cells
Macrophages_M1
Macrophages_M2
Monocytes
Neutrophils
NK_cells
T_cells_CD4
T_cells_CD8
Tregs
Dendritic_cells
Other

PGLYRP1

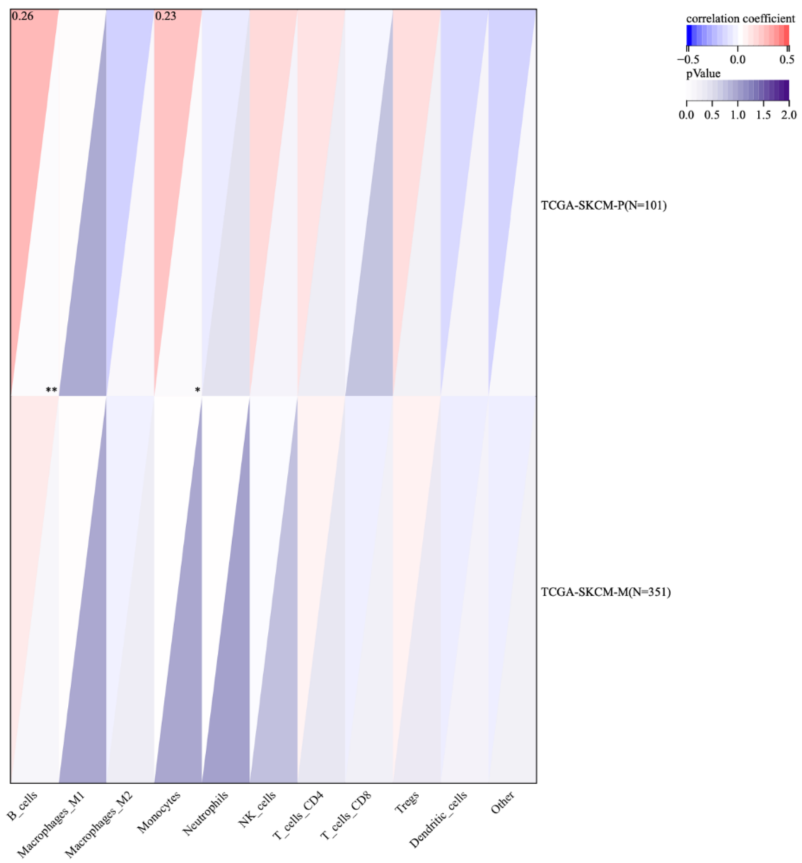

POSTN

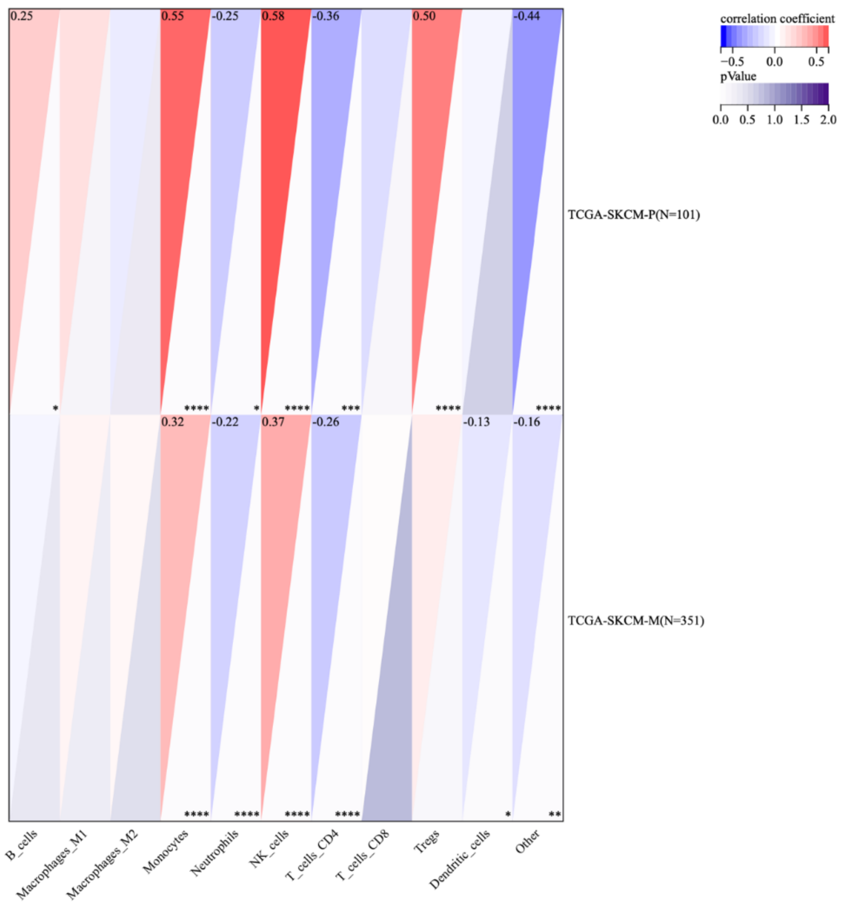

PRG2

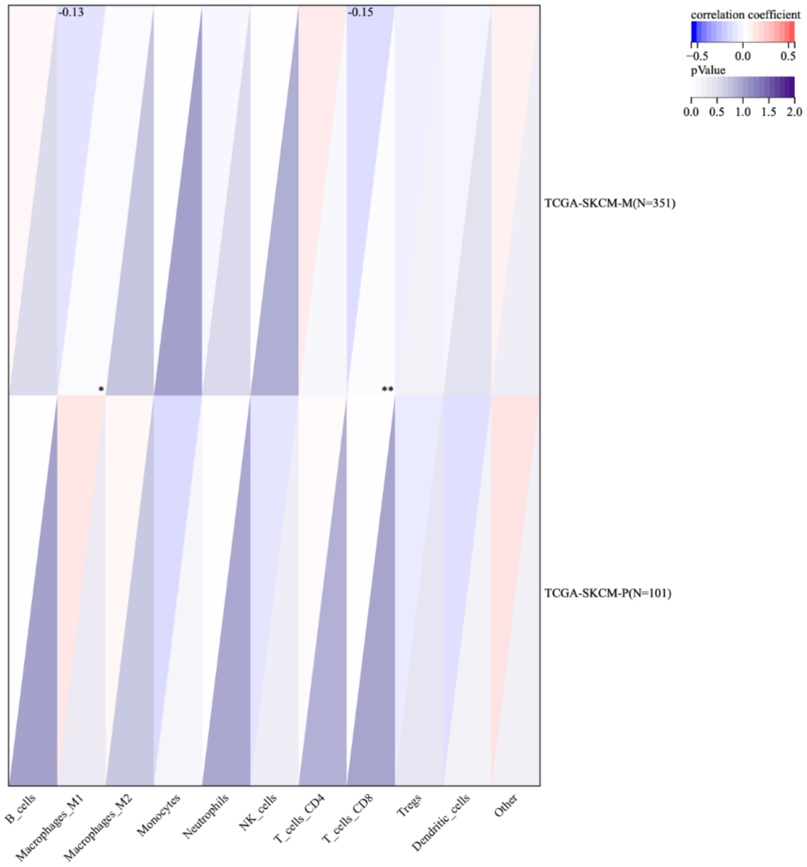
correlation coefficient
−0.5 0.0 0.5
pValue
0.0 0.5 1.0 1.5 2.0
-0.13
-0.15
*
**
TCGA-SKCM-M(N=351)
TCGA-SKCM-P(N=101)
B_cells
Macrophages_M1
Macrophages_M2
Monocytes
Neutrophils
NK_cells
T_cells_CD4
T_cells_CD8
Tregs
Dendritic_cells
Other

PTK2

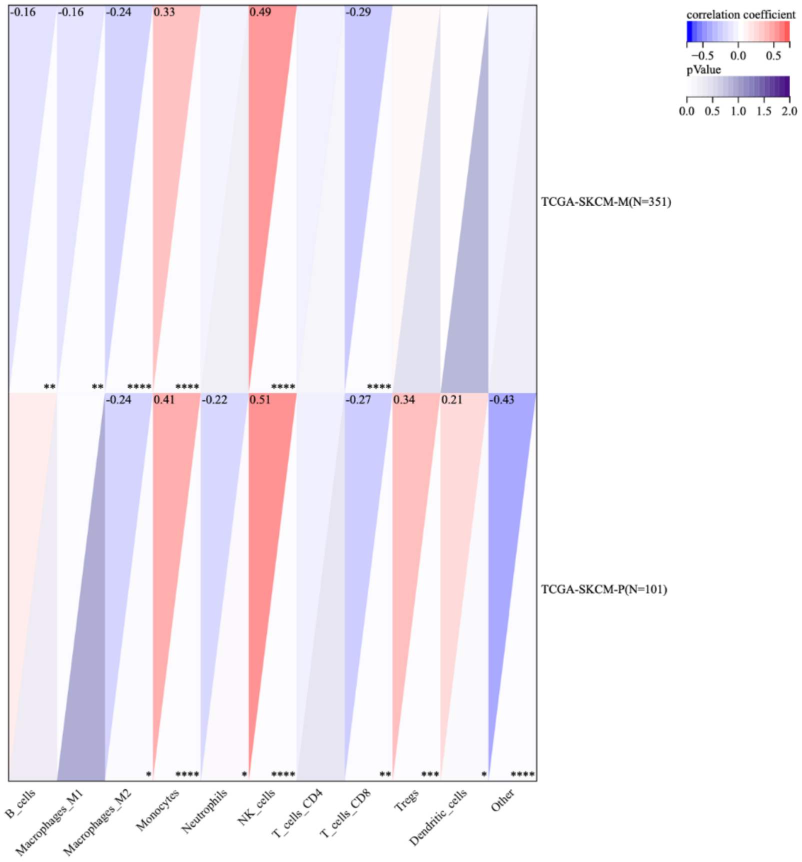
correlation coefficient
−0.5 0.0 0.5
pValue
0.0 0.5 1.0 1.5 2.0
-0.16 -0.16 -0.24 0.33 0.49 -0.29
** ** **** **** **** ****
-0.24 0.41 -0.22 0.51 -0.27 0.34 0.21 -0.43
* **** * **** ** *** * ****
TCGA-SKCM-M(N=351)
TCGA-SKCM-P(N=101)
B_cells
Macrophages_M1
Macrophages_M2
Monocytes
Neutrophils
NK_cells
T_cells_CD4
T_cells_CD8
Tregs
Dendritic_cells
Other

S100A4

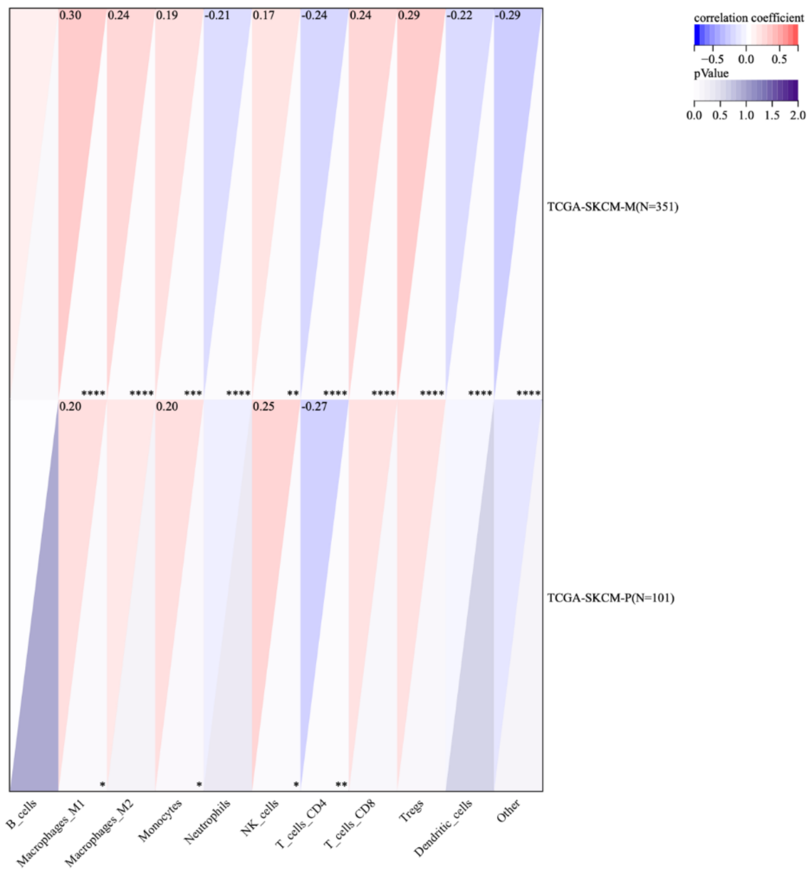

SERPINA5

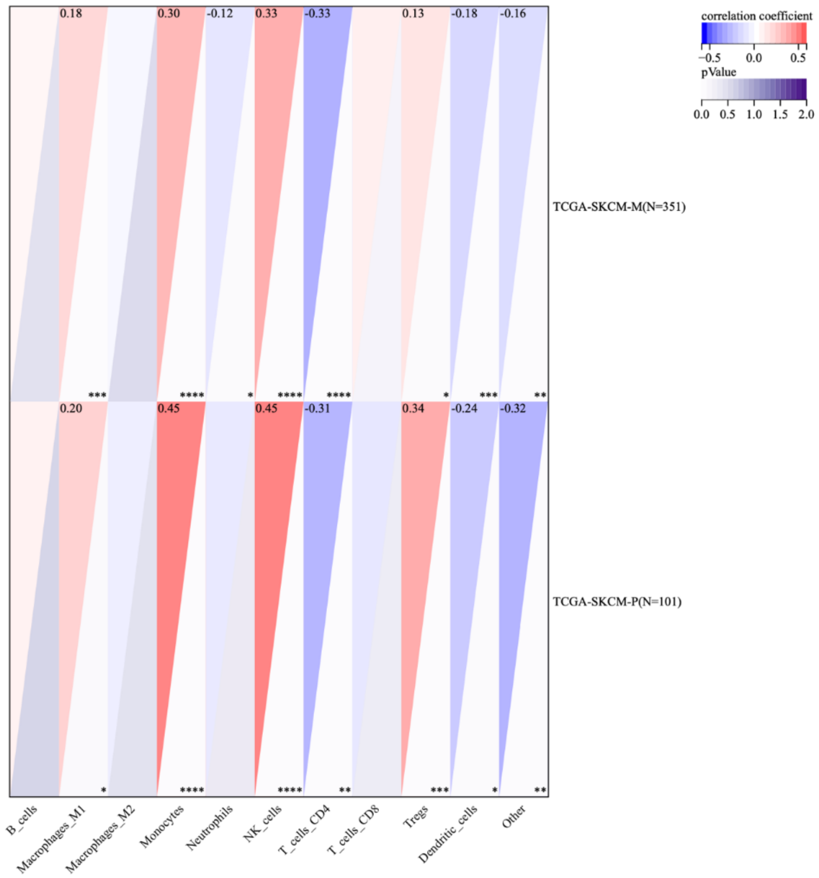

SLCO2A1

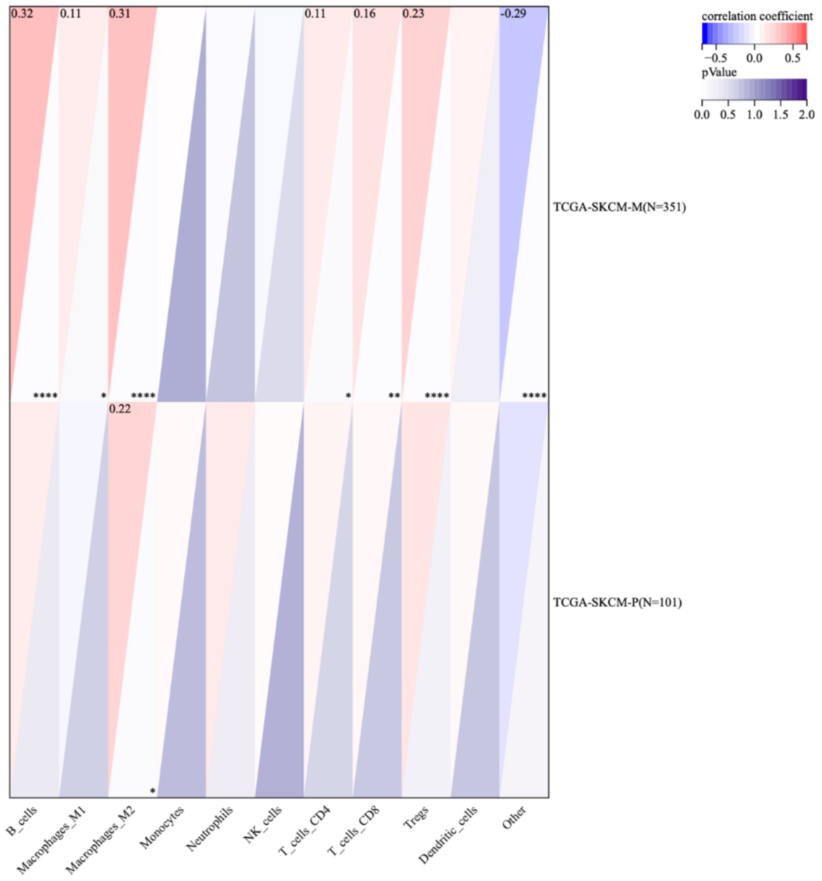

SPP1

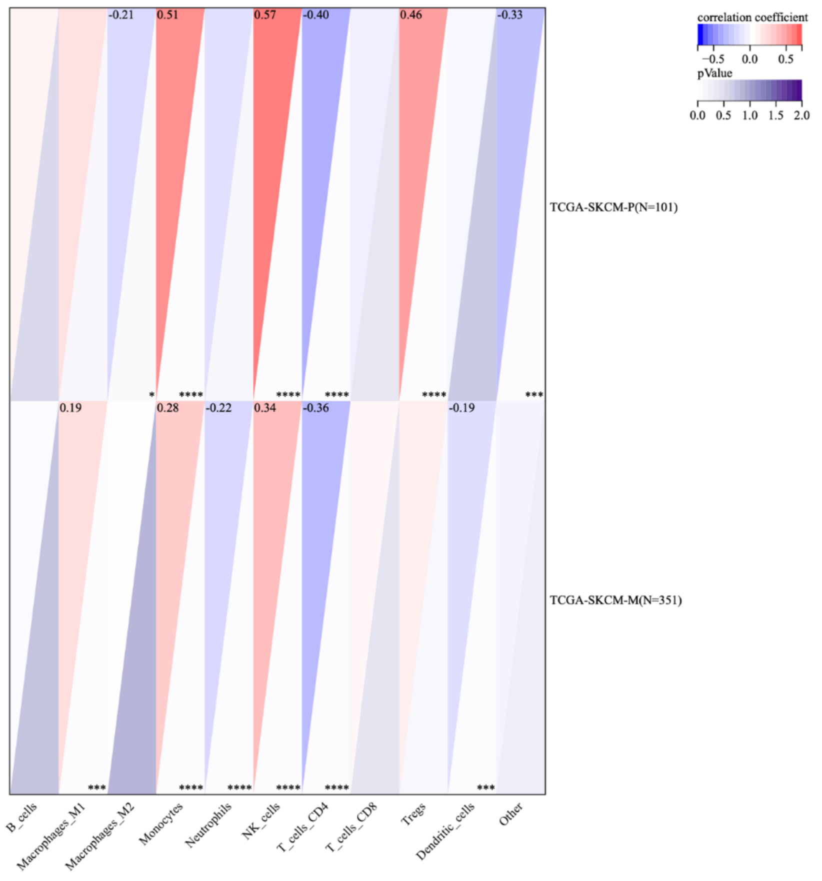

STC1

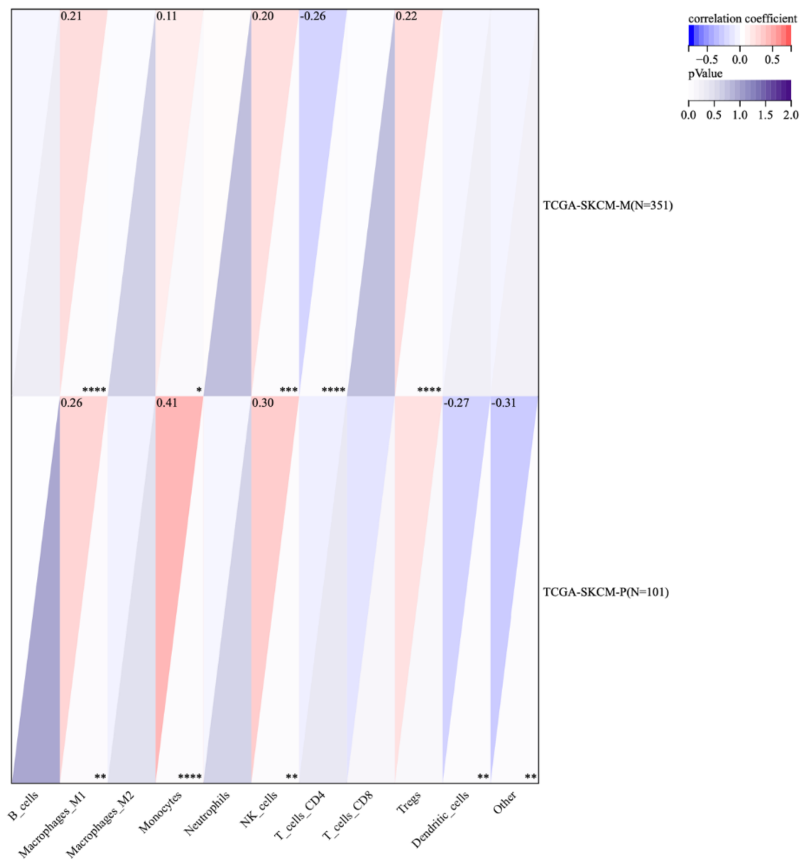
correlation coefficient
−0.5 0.0 0.5
pValue
0.0 0.5 1.0 1.5 2.0
0.21
0.11
0.20
-0.26
0.22
****
*
***
****
****
0.26
0.41
0.30
-0.27
-0.31
**
****
**
**
**
TCGA-SKCM-M(N=351)
TCGA-SKCM-P(N=101)
B_cells
Macrophages_M1
Macrophages_M2
Monocytes
Neutrophils
NK_cells
T_cells_CD4
T_cells_CD8
Tregs
Dendritic_cells
Other

THBD

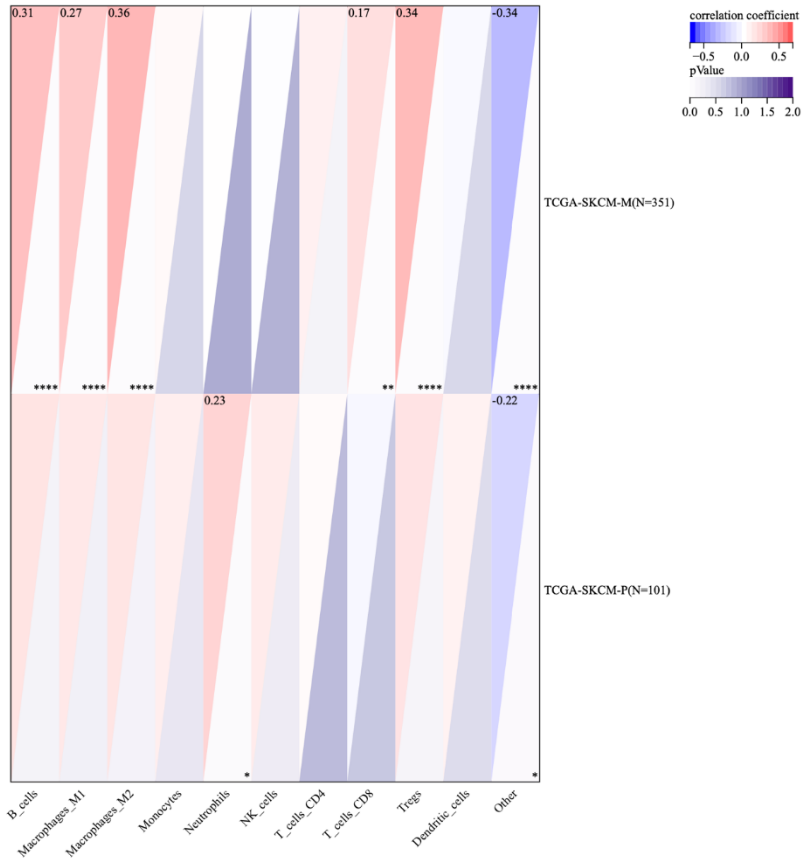
correlation coefficient
−0.5 0.0 0.5
pValue
0.0 0.5 1.0 1.5 2.0
0.31
0.27
0.36
0.17
0.34
-0.34
****
****
****
**
****
****
0.23
-0.22
*
*
TCGA-SKCM-M(N=351)
TCGA-SKCM-P(N=101)
B_cells
Macrophages_M1
Macrophages_M2
Monocytes
Neutrophils
NK_cells
T_cells_CD4
T_cells_CD8
Tregs
Dendritic_cells
Other

TIMP1

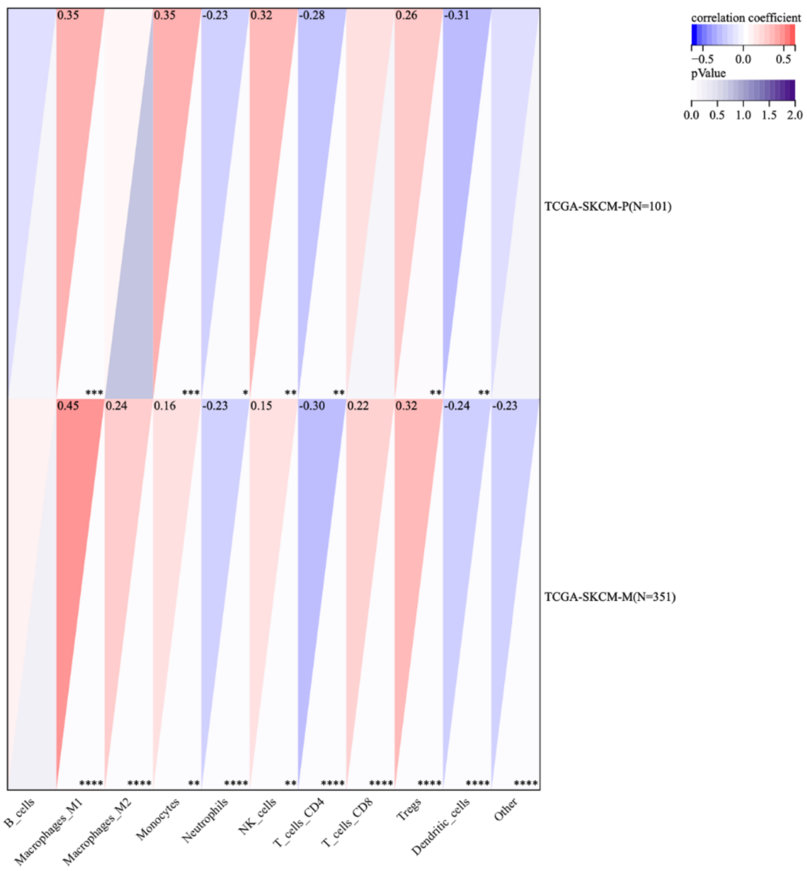

TNFRSF21

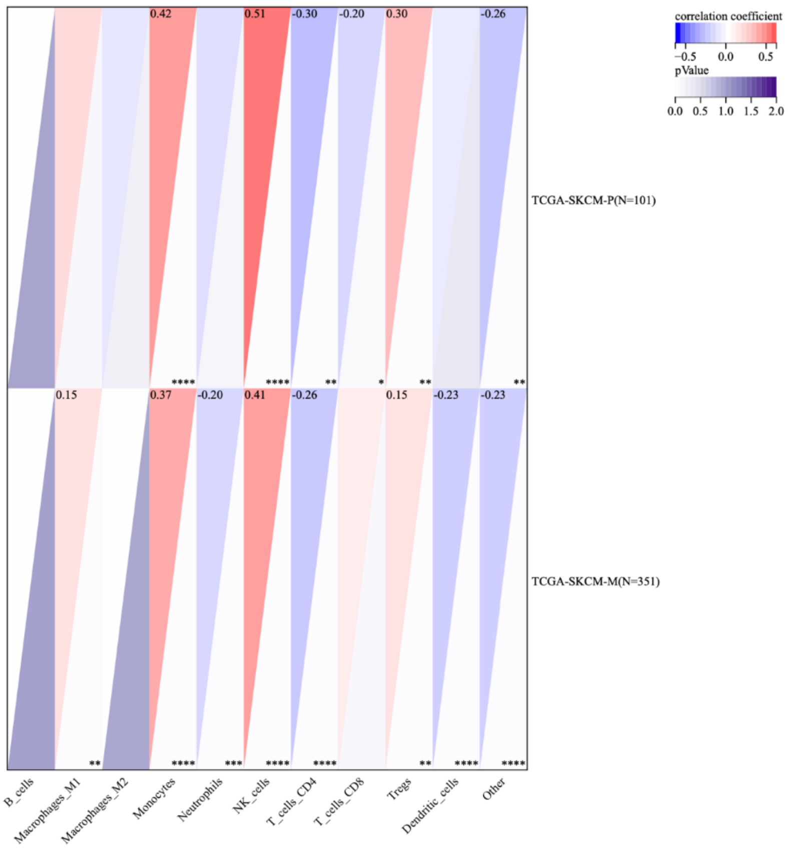

VAV2

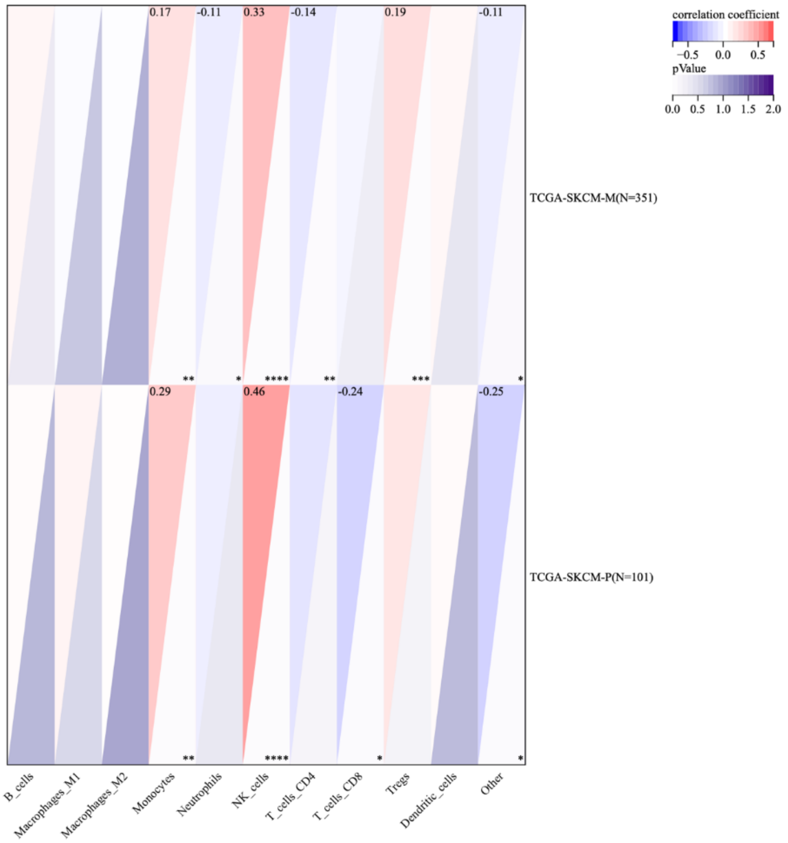

correlation coefficient
−0.5 0.0 0.5
pValue
0.0 0.5 1.0 1.5 2.0
TCGA-SKCM-M(N=351)
TCGA-SKCM-P(N=101)
0.17
-0.11
0.33
-0.14
0.19
-0.11
0.29
0.46
-0.24
-0.25
B_cells
Macrophages_M1
Macrophages_M2
Monocytes
Neutrophils
NK_cells
T_cells_CD4
T_cells_CD8
Tregs
Dendritic_cells
Other

VCAN

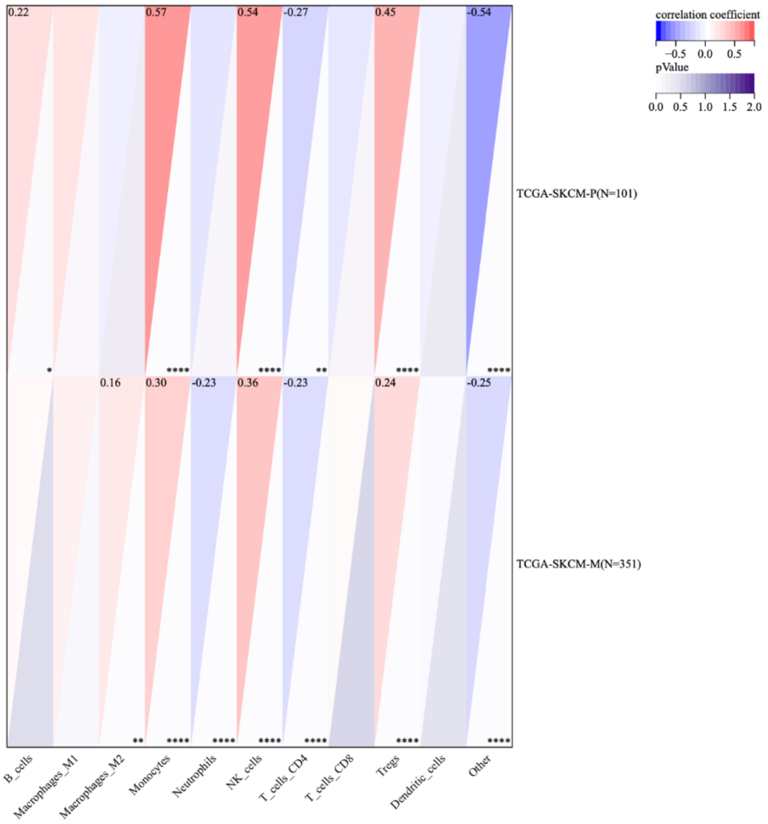

correlation coefficient
−0.5 0.0 0.5
pValue
0.0 0.5 1.0 1.5 2.0
TCGA-SKCM-P(N=101)
TCGA-SKCM-M(N=351)
0.22
0.57
0.54
-0.27
0.45
-0.54
0.16
0.30
-0.23
0.36
-0.23
0.24
-0.25
B_cells
Macrophages_M1
Macrophages_M2
Monocytes
Neutrophils
NK_cells
T_cells_CD4
T_cells_CD8
Tregs
Dendritic_cells
Other

VEGFA

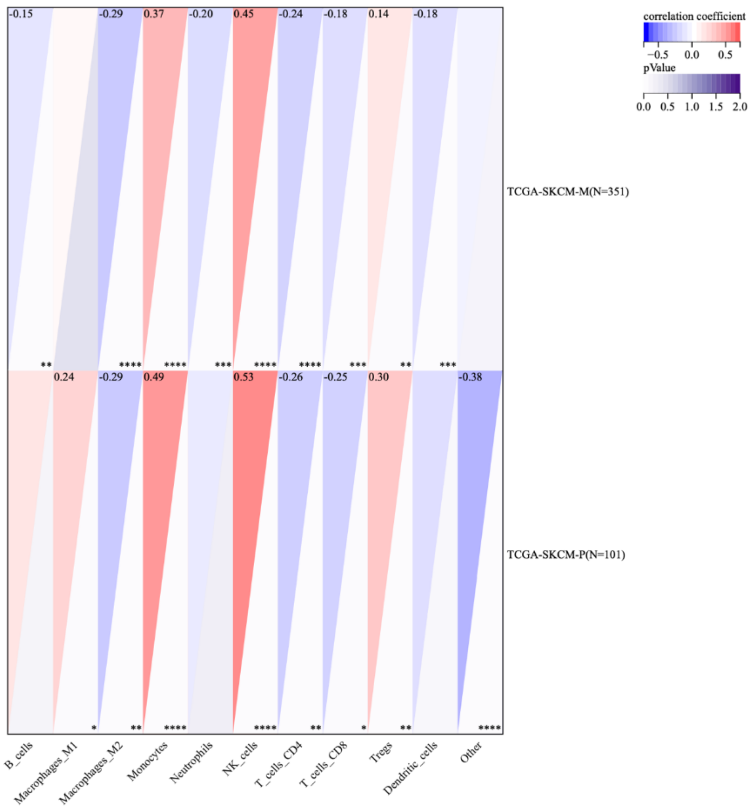

VTN

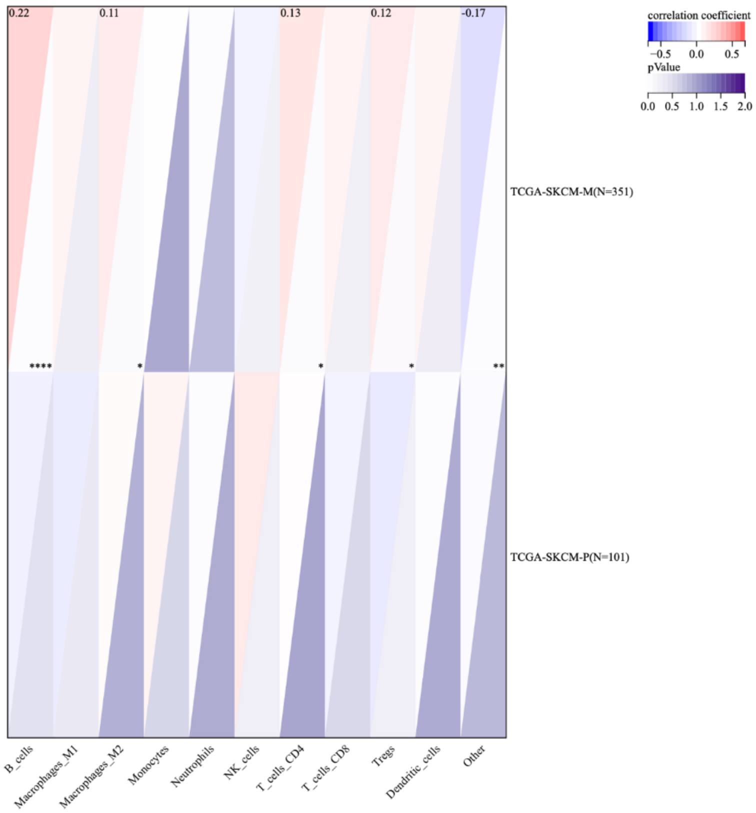

Figure S10. Spearman's correlations between the expression of ARGs and the infiltration in different immune cells in primary and metastatic skin melanoma, using QUANTISEQ.

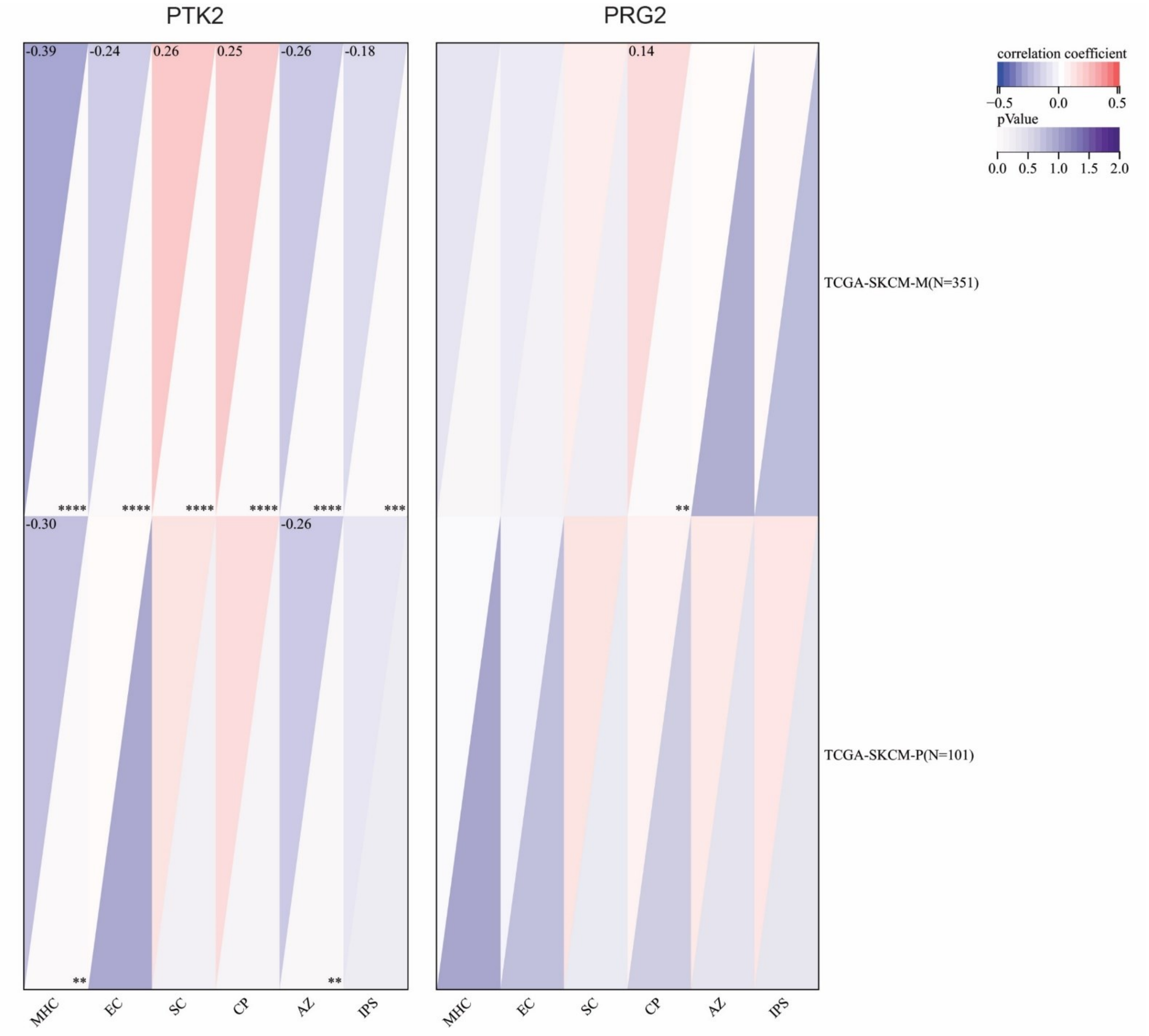

Figure S11 Spearman's correlation analysis between the expression of *PTK2* (or *PRG2*) and immune cell populations in primary (TCGA-SCKM-P) and metastatic skin melanoma (TCGA-SCKM-M), respectively, using Immune cell profiling (IPS). Asterisks denote statistical significance: *, p<0.05; ****, p<0.001.

APOH

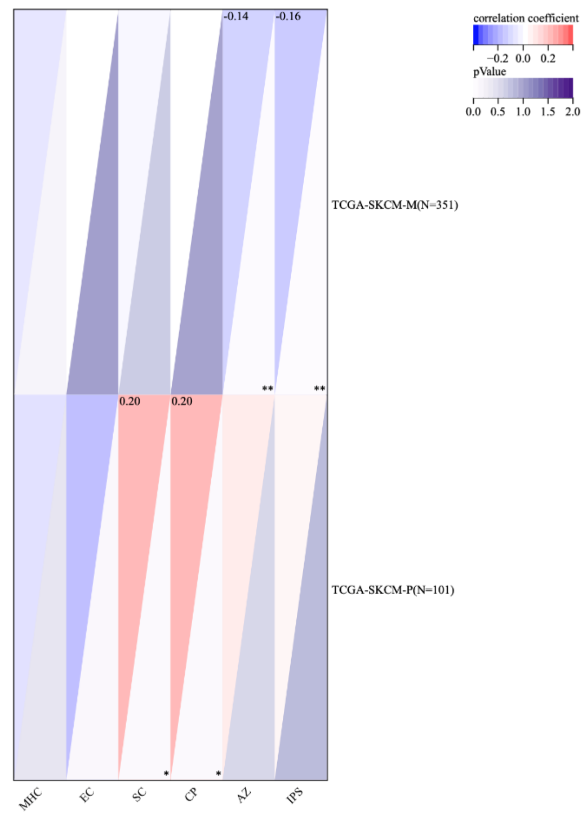

APP

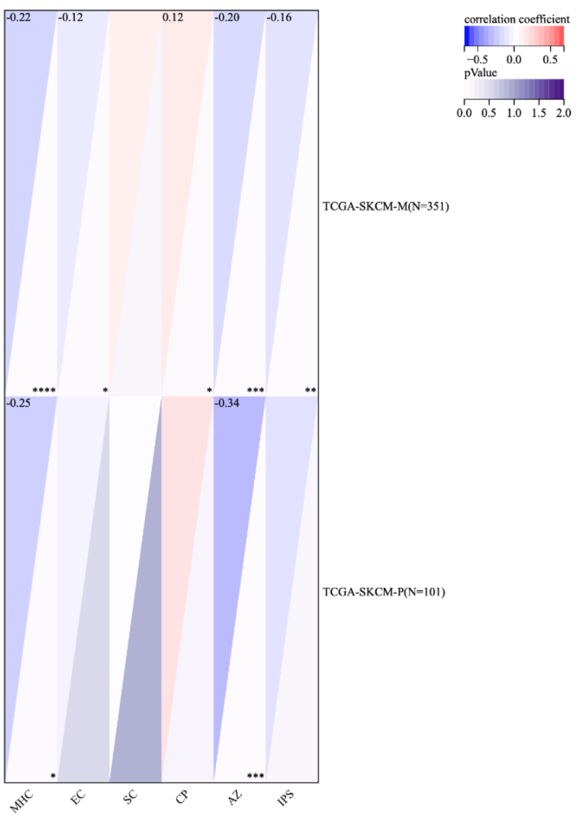

CCND2

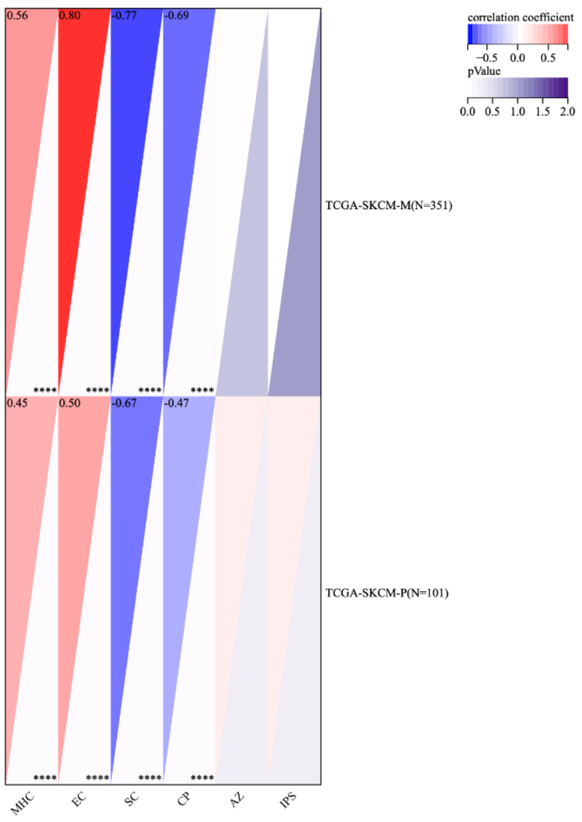

COL3A1

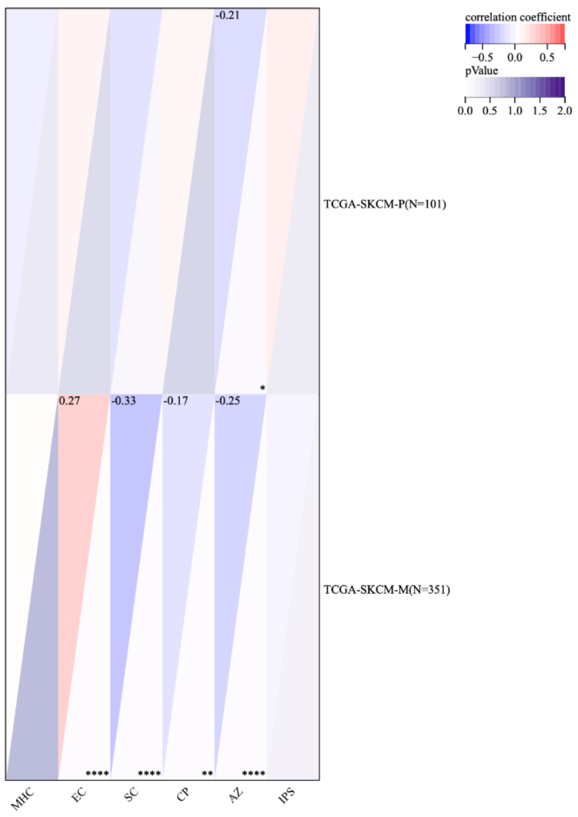

COL5A2

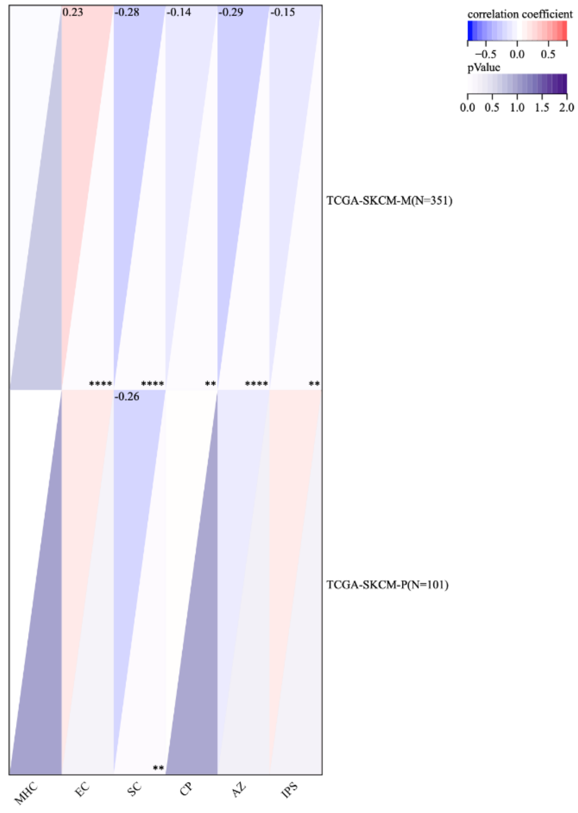

CXCL6

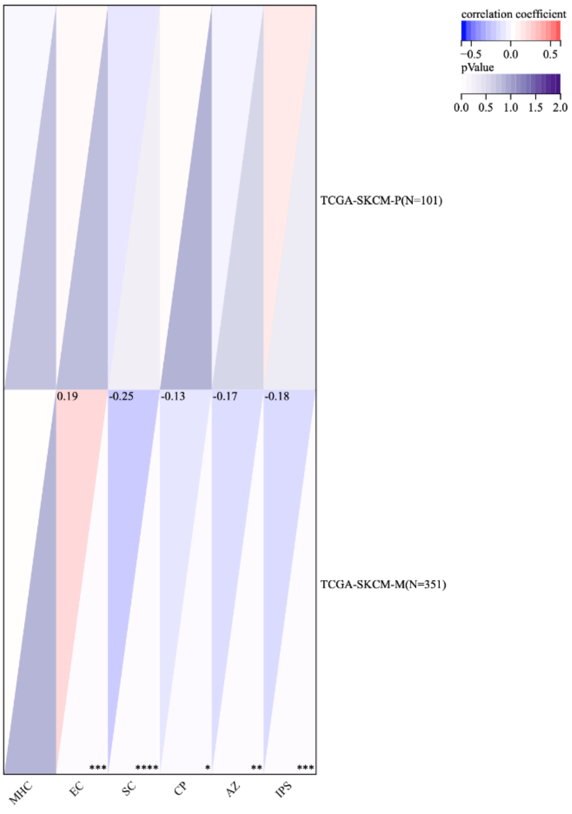

FGFR1

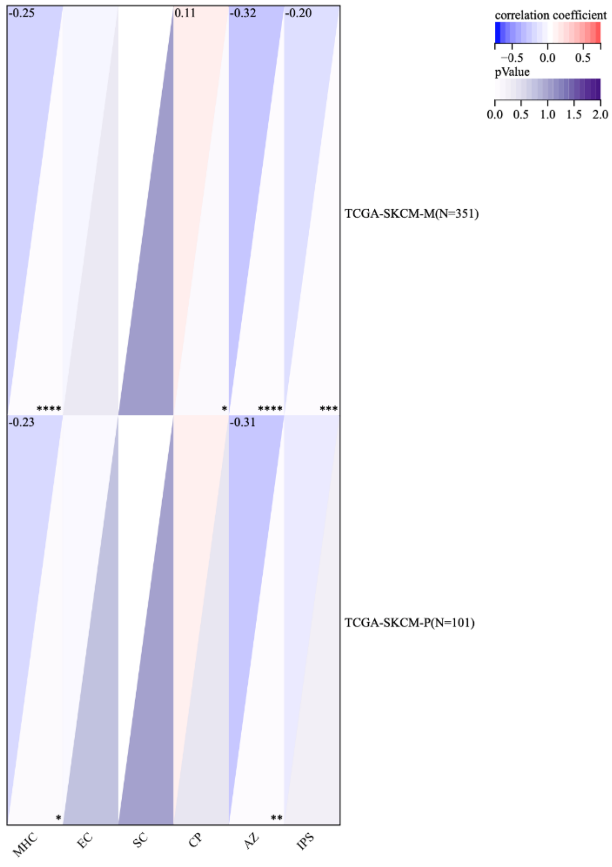

FSTL1

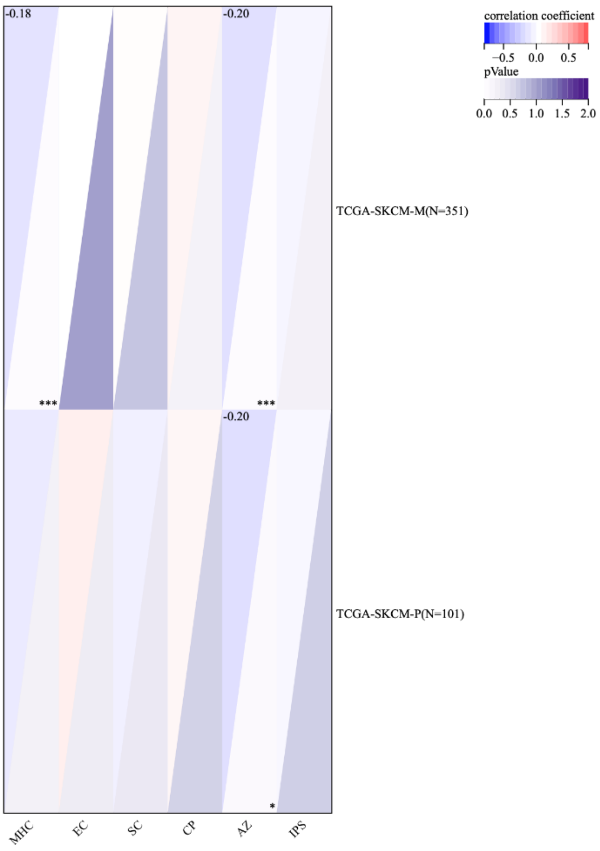

ITGAV

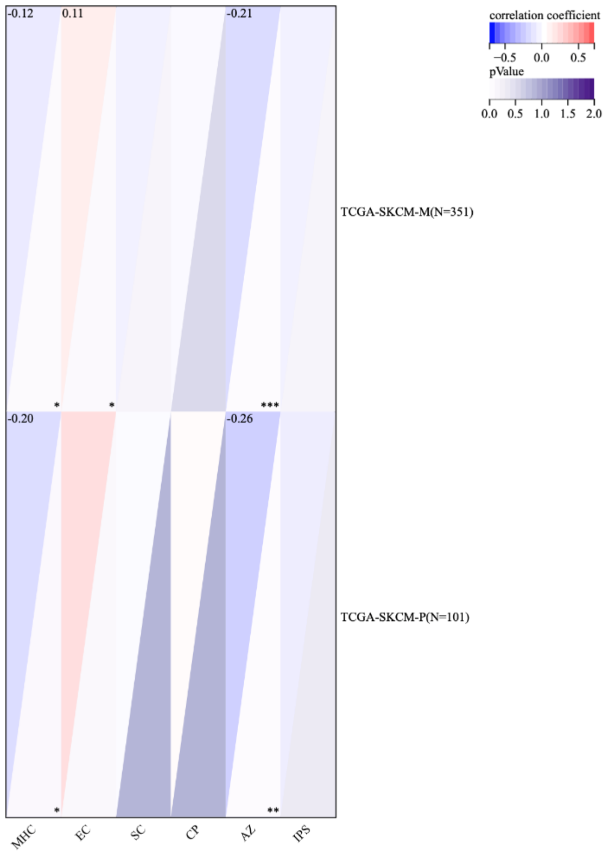

JAG1

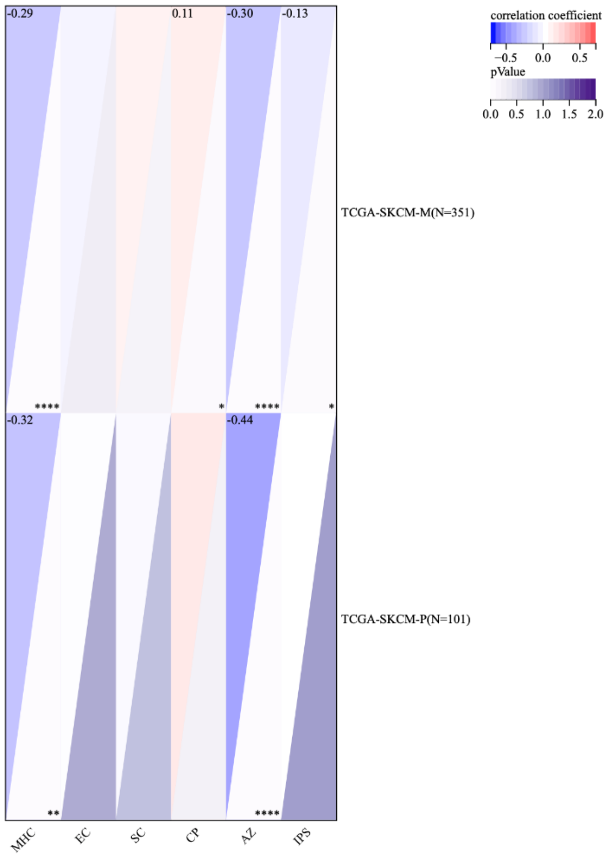

JAG2

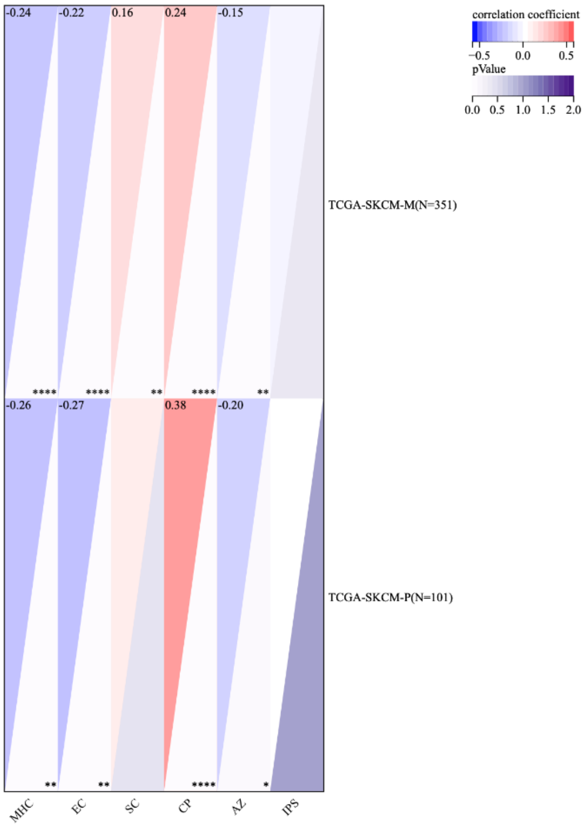

KCNJ8

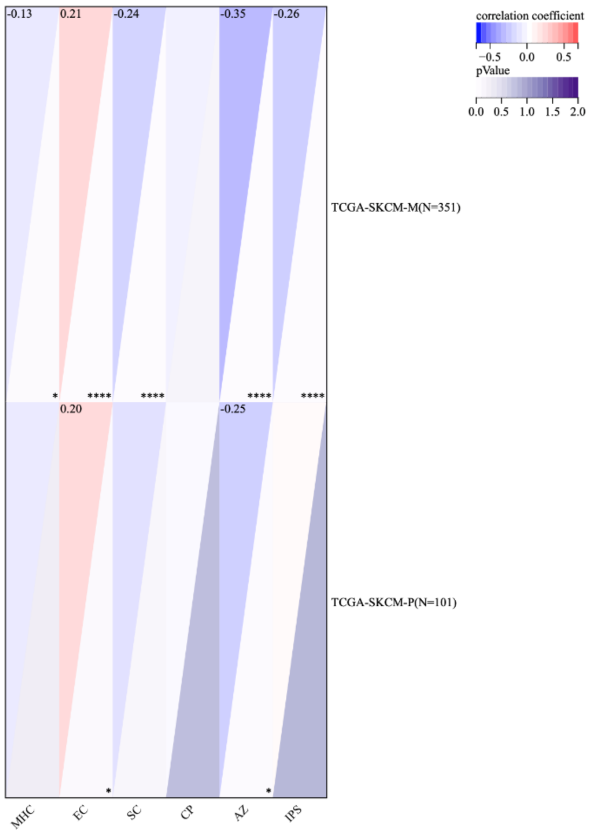

LPL

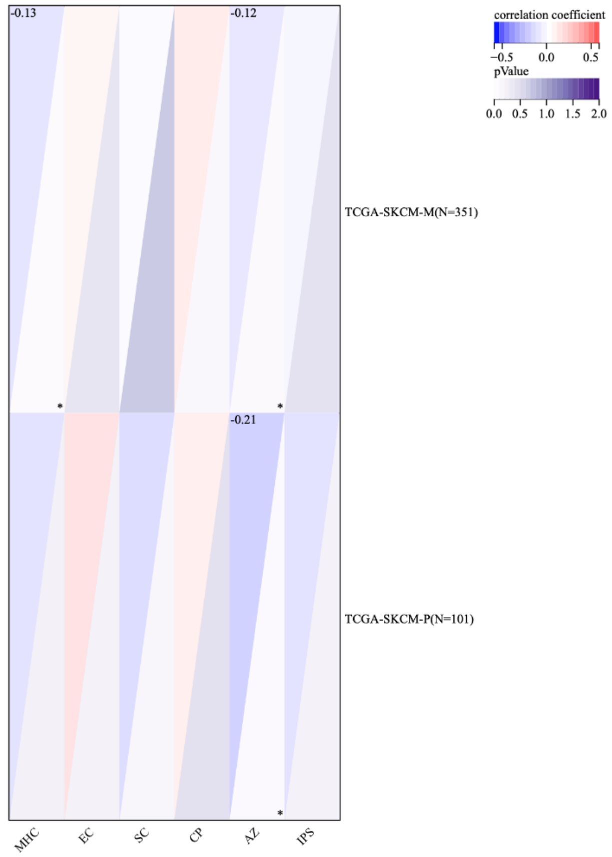

LRPAP1

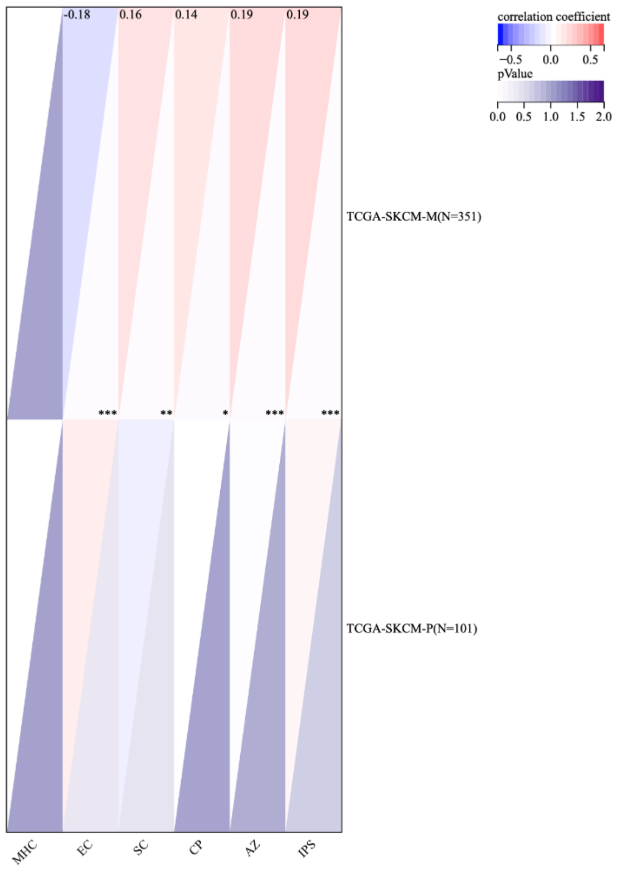

LUM

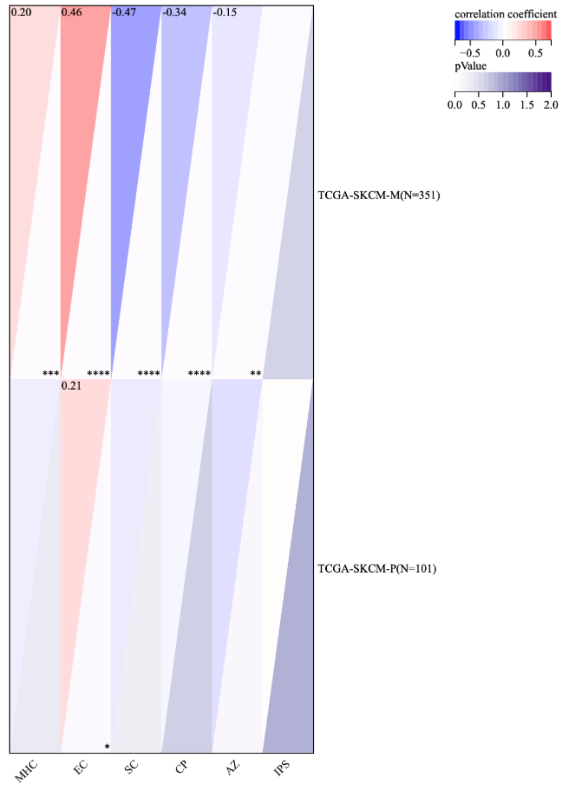

MSX1

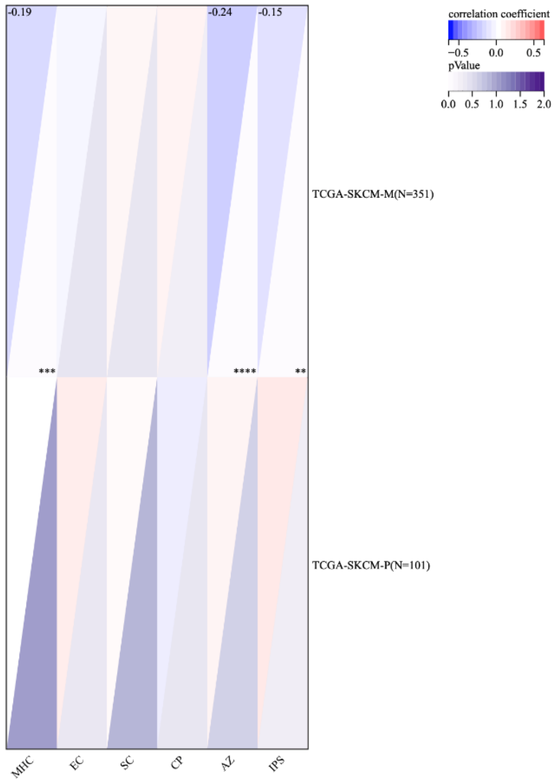

NRP1

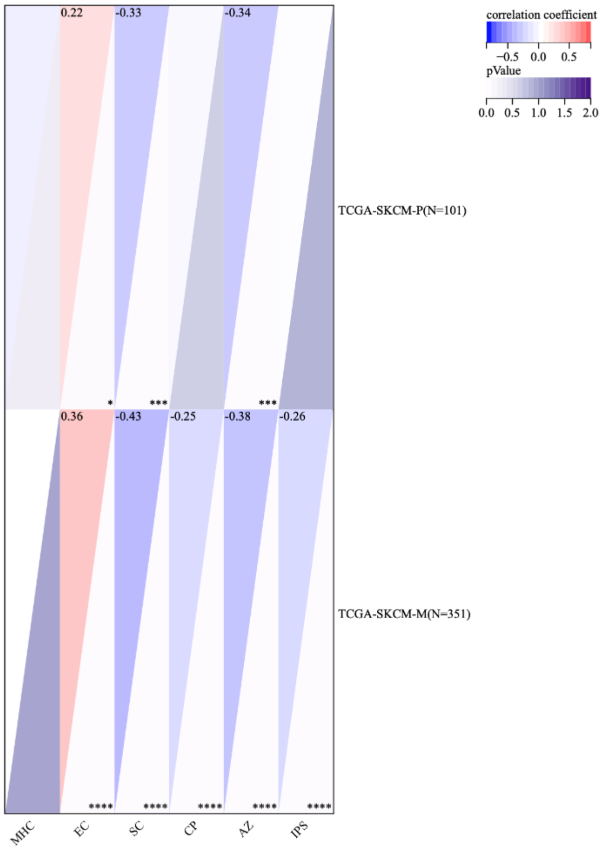

OLR1

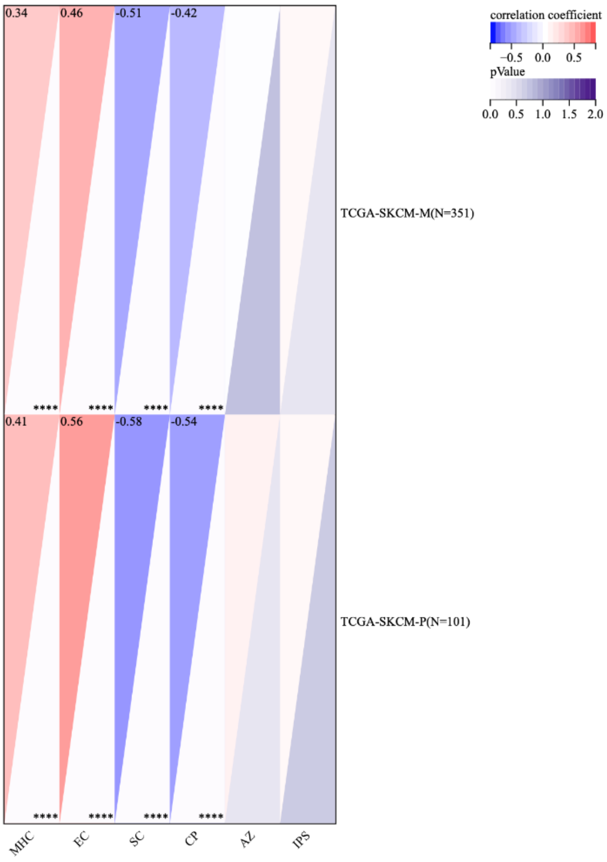

PDGFA

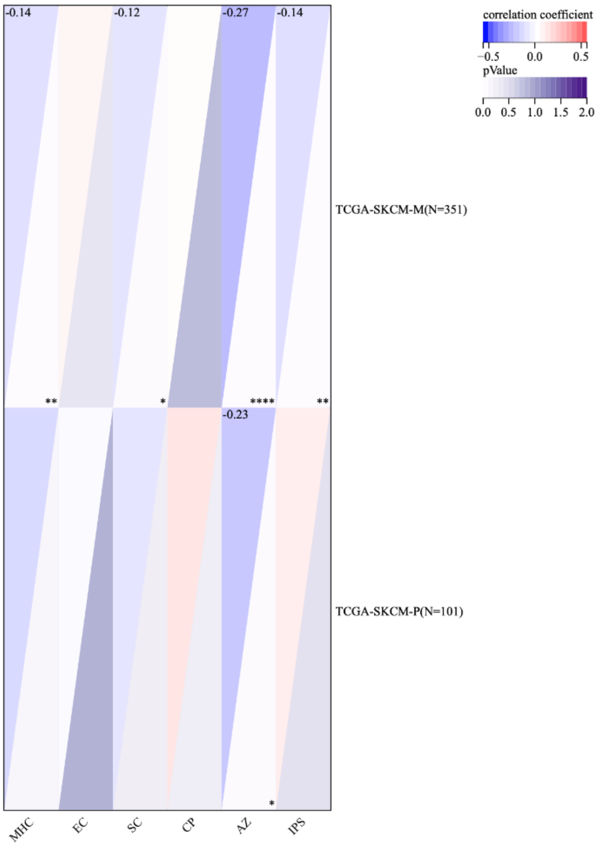

PF4

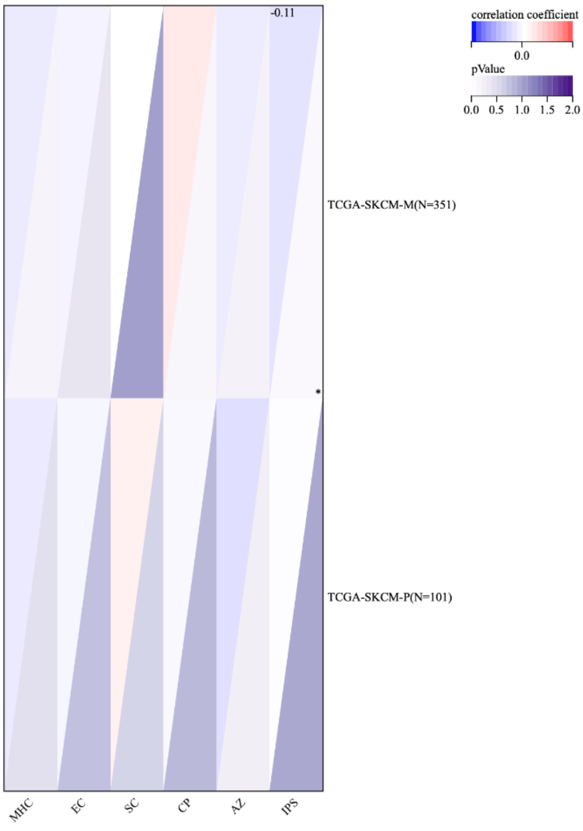

PGLYRP1

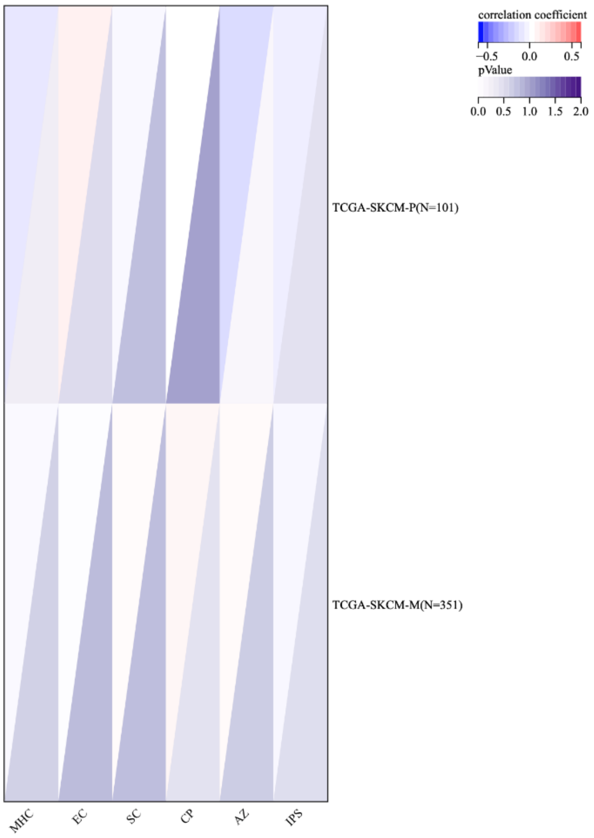

POSTN

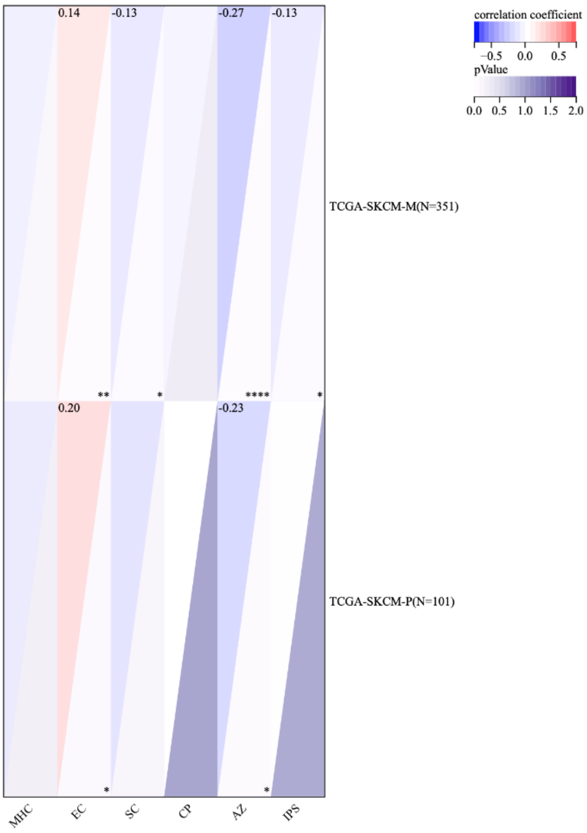

PRG2

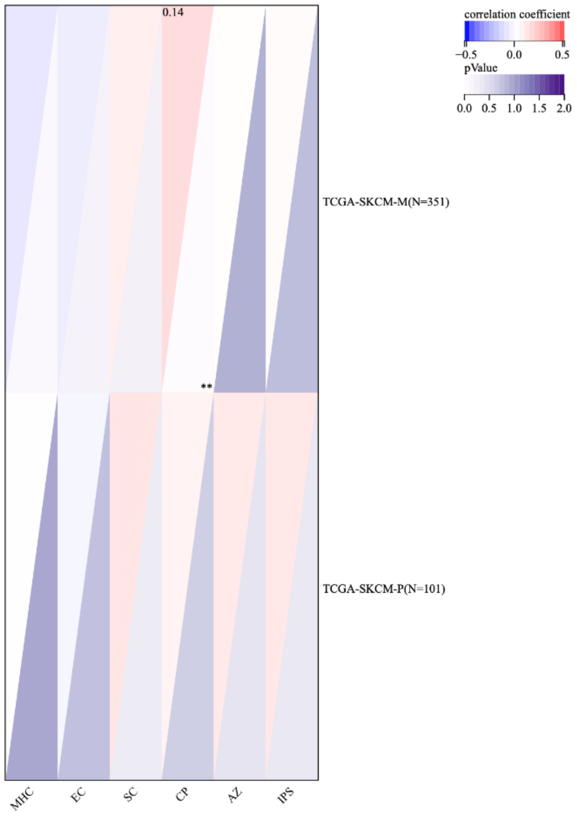

PTK2

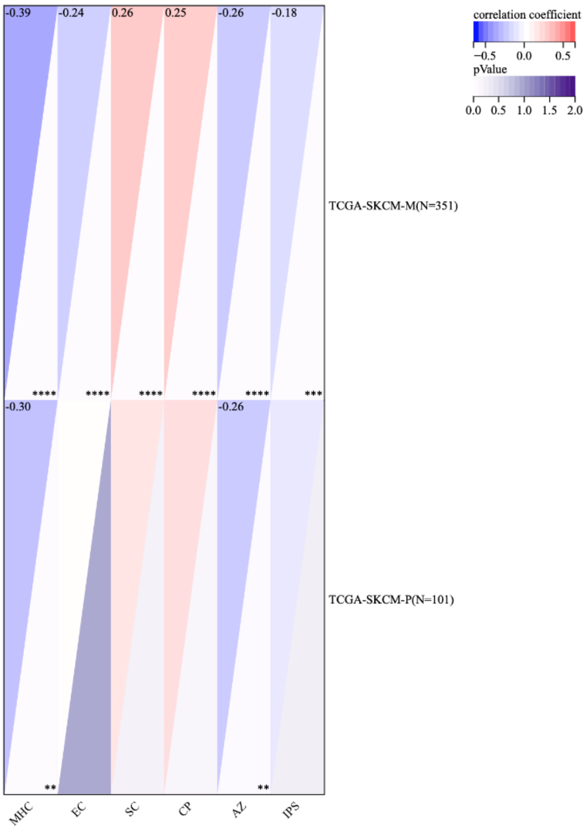

S100A4

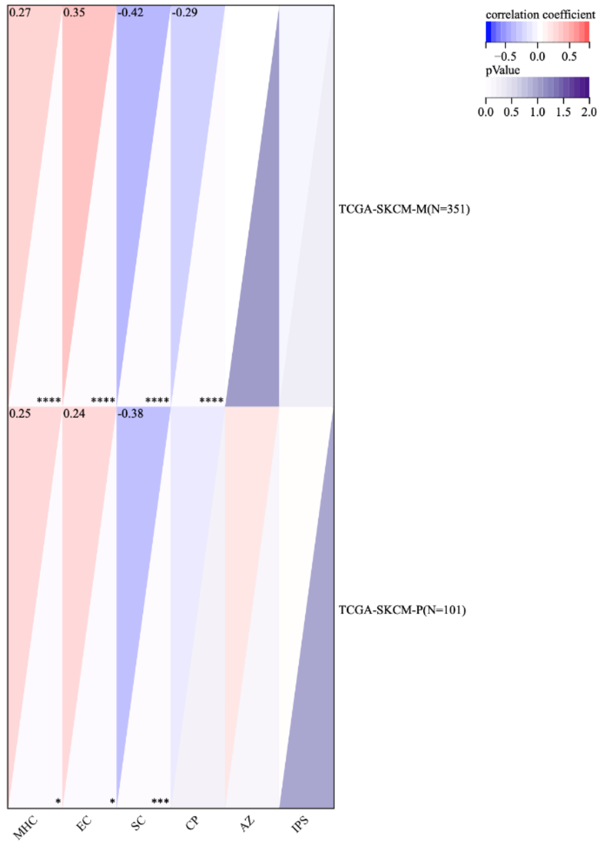

SERPINA5

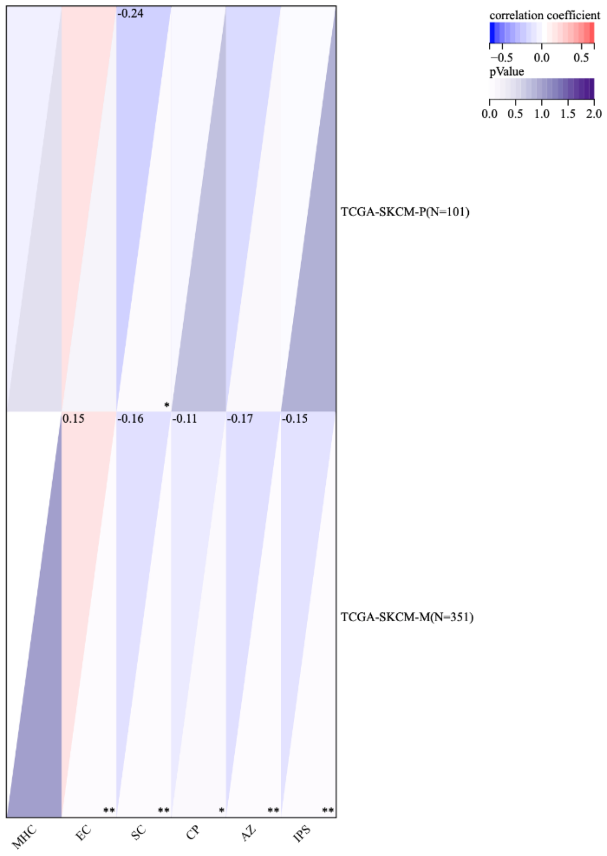

SLCO2A1

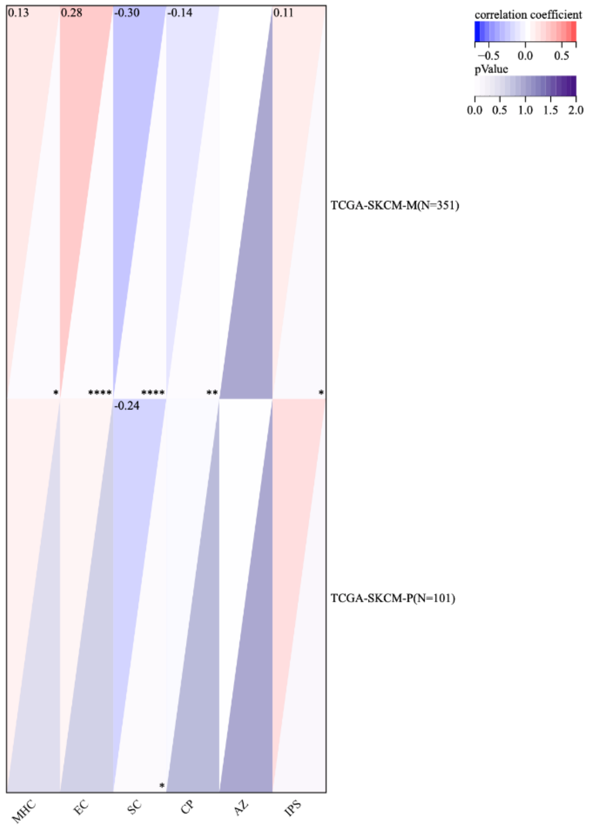

SPP1

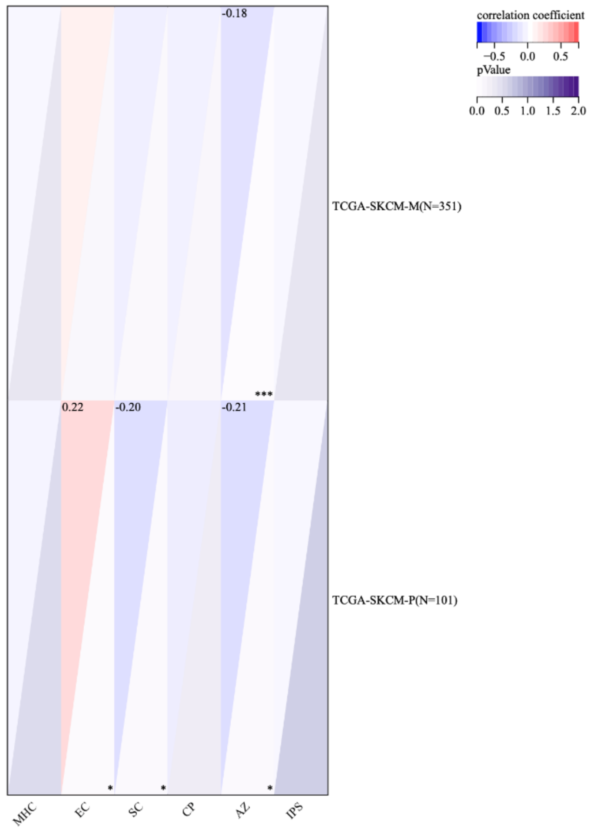

STC1

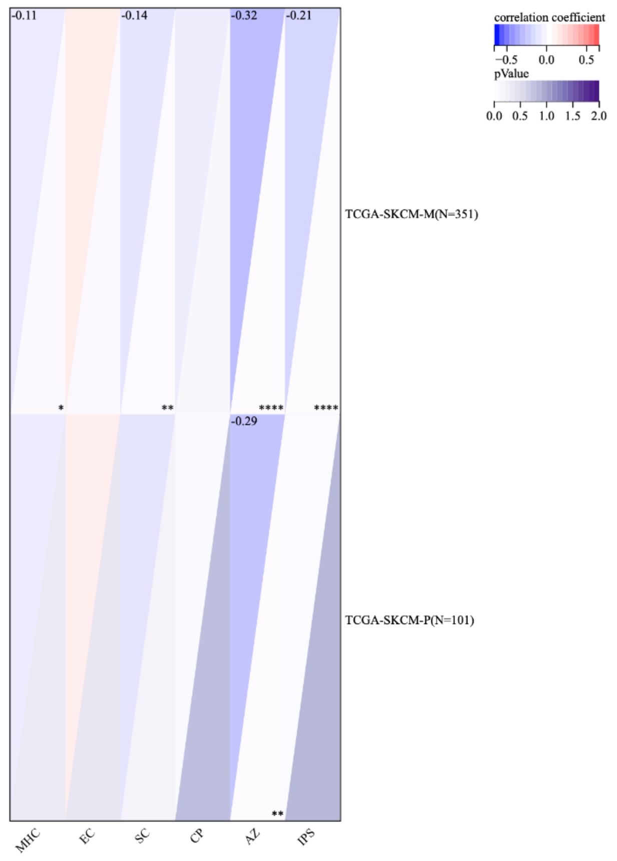

THBD

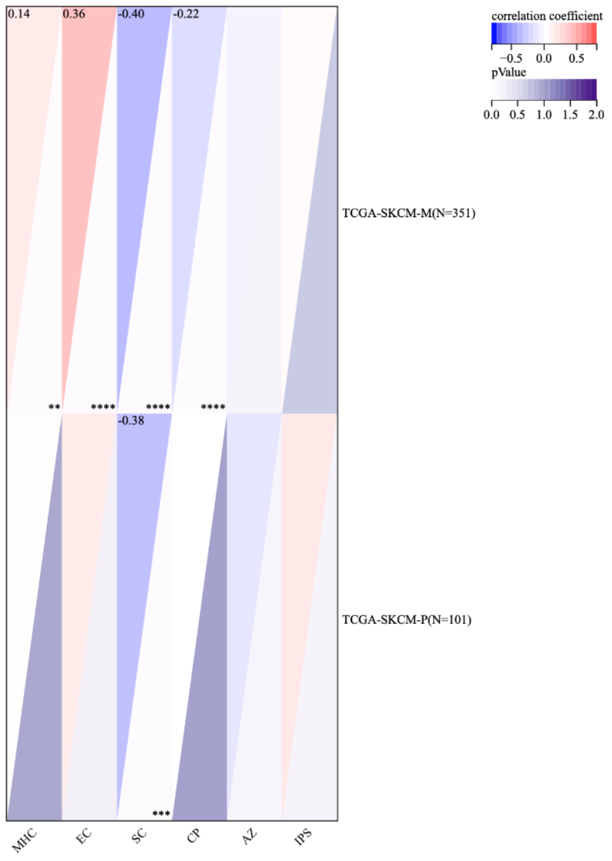

TIMP1

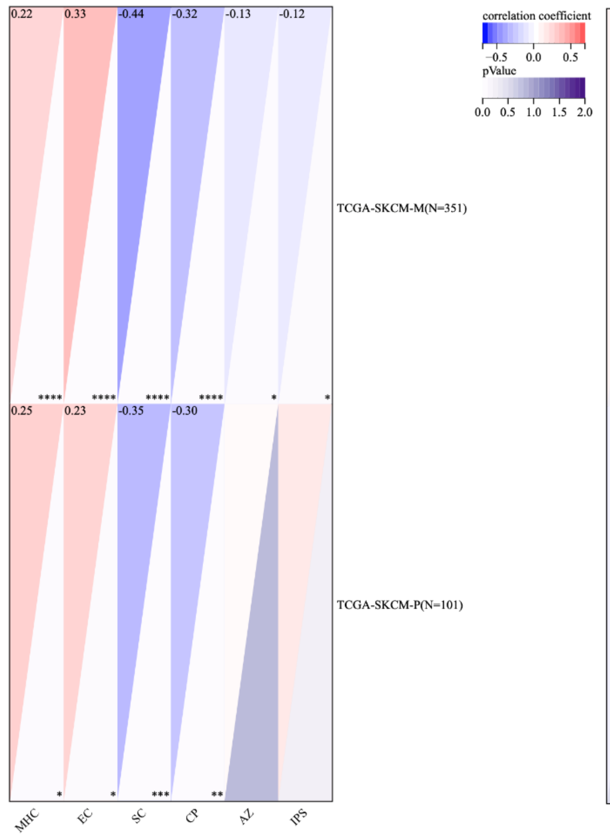

TNFRSF21

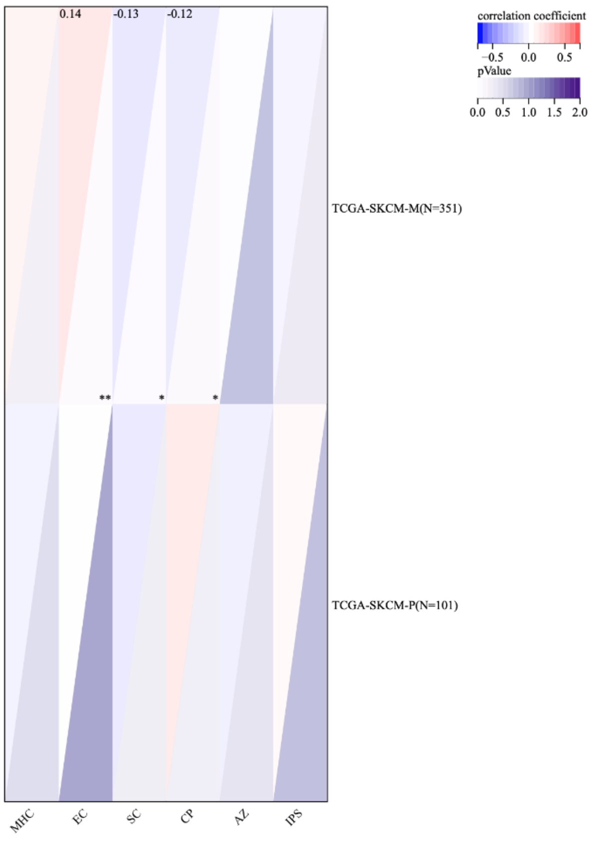

VAV2

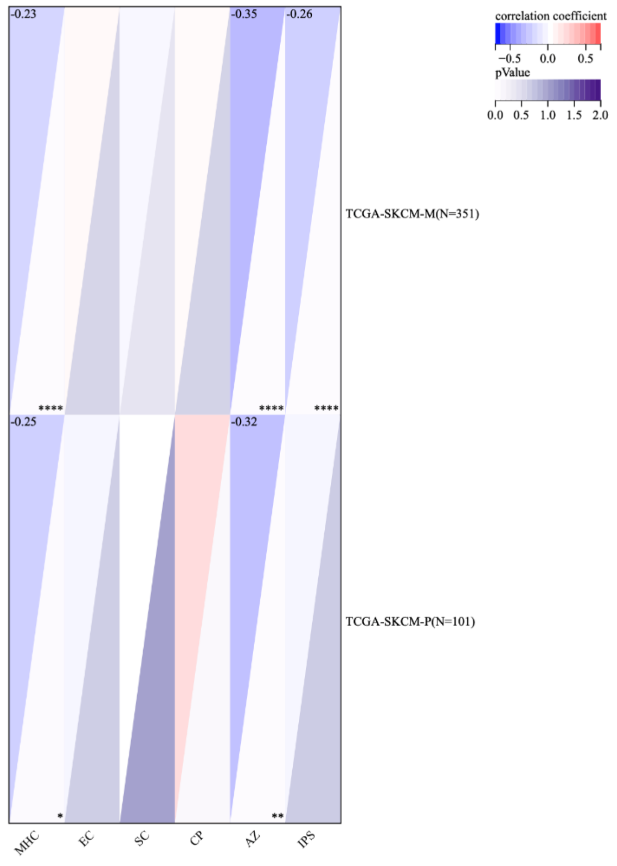

VCAN

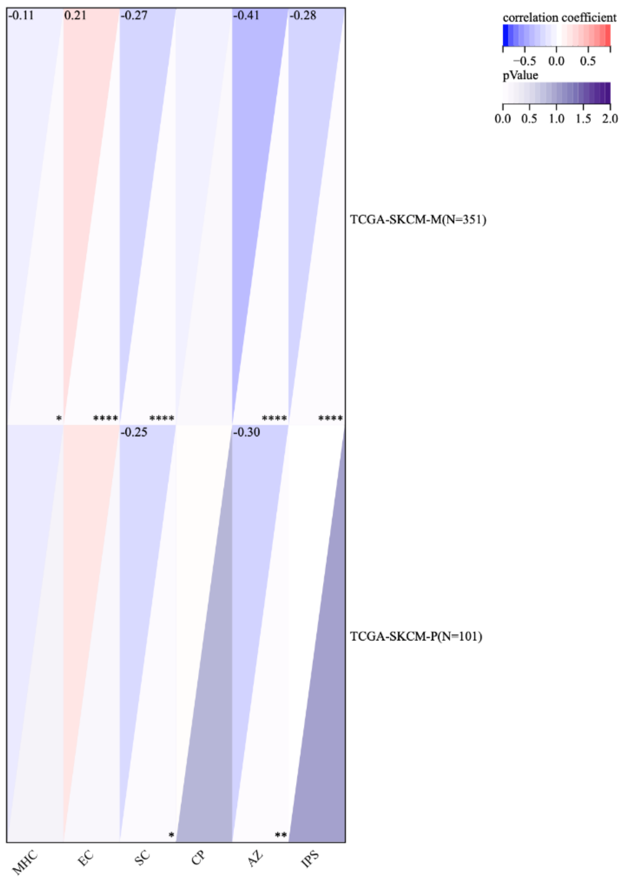

VEGFA

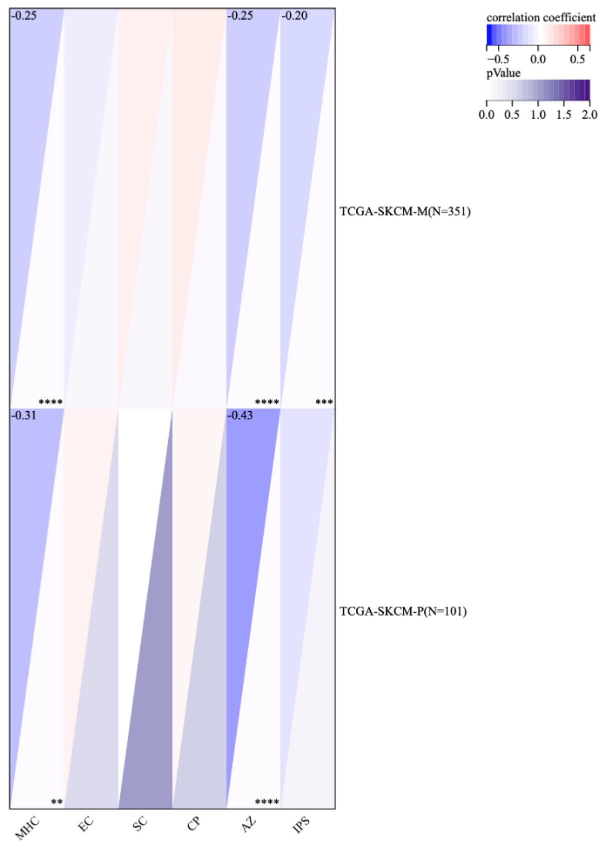

VTN

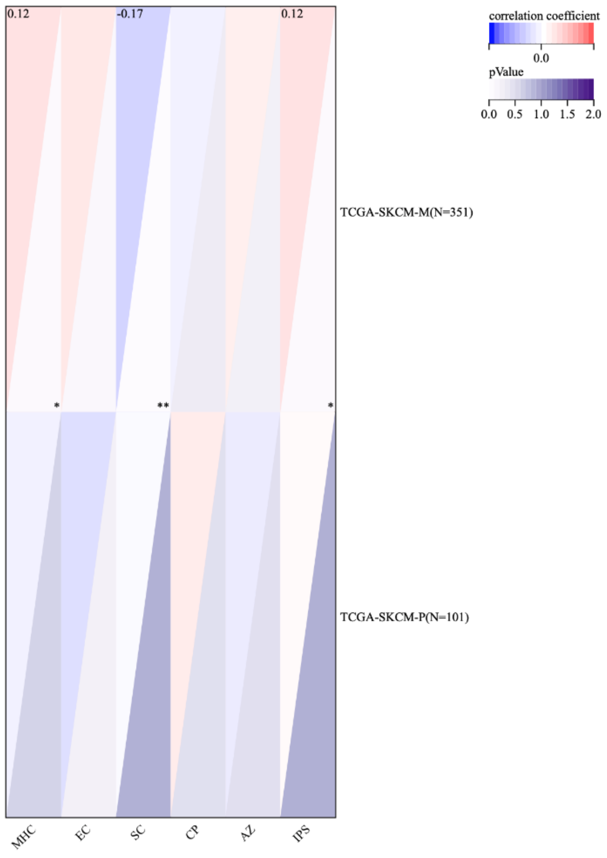

Figure S12. Spearman's correlations between the expression of ARGs and the infiltration in different immune cells in primary and metastatic skin melanoma, using IPS. Asterisks denote statistical significance: *, $p<0.05$; ****, $p<0.001$.
